# Male- and female-specific reproductive risk factors across the lifespan for dementia or cognitive decline: a systematic review and meta-analysis

**DOI:** 10.1186/s12916-023-03159-0

**Published:** 2023-11-23

**Authors:** Shuang-Ling Han, De-Chun Liu, Chen-Chen Tan, Lan Tan, Wei Xu

**Affiliations:** 1 Department of Neurology, Qingdao Municipal Hospital Group, Qingdao University, Donghai Middle Road, No.5, Qingdao, 266000 China; 2 Medical College, Qingdao University, Qingdao, 266000 China; 3 Department of Obstetrics, Qingdao Municipal Hospital Group, Qingdao, 266000 China

**Keywords:** Obstetrics, Dementia, Cognitive decline, Meta-analysis, Systematic review

## Abstract

**Background:**

Sex difference exists in the prevalence of dementia and cognitive decline. The impacts of sex-specific reproductive risk factors across the lifespan on the risk of dementia or cognitive decline are still unclear. Herein, we conducted this systemic review and meta-analysis to finely depict the longitudinal associations between sex-specific reproductive factors and dementia or cognitive decline.

**Methods:**

PubMed, EMBASE, and Cochrane Library were searched up to January 2023. Studies focused on the associations of female- and male-specific reproductive factors with dementia or cognitive decline were included. Multivariable-adjusted effects were pooled via the random effect models. Evidence credibility was scored by the GRADE system. The study protocol was pre-registered in PROSPERO and the registration number is CRD42021278732.

**Results:**

A total of 94 studies were identified for evidence synthesis, comprising 9,839,964 females and 3,436,520 males. Among the identified studies, 63 of them were included in the meta-analysis. According to the results, seven female-specific reproductive factors including late menarche (risk increase by 15%), nulliparous (11%), grand parity (32%), bilateral oophorectomy (8%), short reproductive period (14%), early menopause (22%), increased estradiol level (46%), and two male-specific reproductive factors, androgen deprivation therapy (18%), and serum sex hormone–binding globulin (22%) were associated with an elevated risk of dementia or cognitive decline.

**Conclusions:**

These findings potentially reflect sex hormone-driven discrepancy in the occurrence of dementia and could help build sex-based precise strategies for preventing dementia.

**Supplementary Information:**

The online version contains supplementary material available at 10.1186/s12916-023-03159-0.

## Background

Strong evidence shows a sex discrepancy in the incidence of dementia and cognitive decline, and this discrepancy varies with age. A recently conducted study with 29,850 participants from 21 cohorts across 6 continents found that women had a higher incidence of dementia compared to men, especially in low- and lower-middle-income countries [1]. The estimation of the global prevalence of dementia published in the Lancet found more women diagnosed than men in 2019, with this disparity expected to persist until 2050 [2]. The lifetime risk of Alzheimer’s disease (AD) in females was nearly double that of men, as assessed by the Framingham Heart Study [3]. Cognitive decline was more severe in women, and the shift from mild cognitive impairment to dementia occurred more quickly [4]. Sex-specific biochemical processes may partly explain these disparities.

Investigating the correlations between sex-specific reproductive characteristics and dementia can provide more convincing insights into sex-related differences in dementia. The life of a woman can be divided into four stages: puberty, latency, peri-menopause, and menopause. The timing of the endocrine transition phases like menarche and menopause, both female-specific and age-related, is crucial for the separation standard. Furthermore, females undergo special biological processes, such as pregnancy and breastfeeding. However, whether these female-specific factors can further influence cognition remains debatable. A cohort study of 4 million participants found that later menarche increased the risk of dementia, while later menopause and longer reproductive period decreased the risk [5]. However, another study contradicted these findings, reporting no associations between menstrual, reproductive, or menopausal factors and incident risk of dementia [6].

Male-specific reproductive factors are less distinct across the lifespan than female-specific factors, and the separation of male phases based on reproductive factors has not been clearly defined. However, emerging studies focusing on the relationship between male-specific hormone-related factors, such as androgen deprivation therapy (ADT) and dementia have yielded contradictory results [7, 8]. Herein, we conducted this systematic review and meta-analysis to thoroughly examine the longitudinal relationships between sex-specific reproductive risk factors and dementia or cognitive decline in order to provide insight into the sex discrepancy.

## Methods

### Search strategy and selection criteria

We followed the updated Preferred Reporting Items for Systematic Reviews and Meta-Analyses (PRISMA 2020) statement’s instructions [9]. Systematic searches were conducted through PubMed, EMBASE, and Cochrane Library principally up to January 1st, 2023, for studies focusing on the associations of female- and male-specific factors with dementia or cognitive decline. As there is already extensive literature on studies of hormone replacement treatment (HRT) in females, this paper will not discuss that topic [10]. Leveraging prior research [11] and guidance from experienced gynecologists and andrologists, we identified female-specific reproductive risk factors using the following search strategy: menarche, placental bed disorders, menstrual cycle, polycystic ovarian syndrome, reproductive period, menopause, climacteric symptoms, reproductive history, estradiol, sex hormone–binding globulin, pregnancy, gravidity, parity, breastfeeding, intrauterine growth retardation, preterm delivery, stillbirth, induced abortion, gestational diabetes, ectopic pregnancy, hyperemesis gravidarum, gestational hypertension, ovarian hyperstimulation, in vitro fertilization, oophorectomy, hysterectomy, subfertility, cesarean section, natural labor, dystocia, fetal death, multiple pregnancies, postpartum hemorrhage, amniotic fluid embolism, puerperal infection, postpartum depression, hyperprolactinemia, amenorrhea, premature ovarian failure, hypothalamus-pituitary-ovary axis, test tube baby, premature rupture of membrane, intrahepatic cholestasis of pregnancy. The following strategy was used for male-specific reproductive risk factors: erectile dysfunction, testosterone, dihydrotestosterone, androstanediol glucuronide, androgen, androgen deprivation therapy, orchiectomy, haplotype Y chromosome cryptorchidism, prostatic hyperplasia, cryptorchidism, varicocele. The literature search strategy included the following terms for outcomes: Alzheimer, dementia, cognitive, and cognition. The main outcome was dementia or cognitive decline. When multiple outcome types were reported for a factor, findings were categorized into the subgroups cognitive decline, dementia, and AD.

The inclusion criteria were as follows: (1) Studies were required to be cohort studies, however, for female reproductive factors (menstrual factors, parity, and gynecological operations), besides cohort studies, case–control studies were also included given the expectation of modest recall bias; (2) The measurement of the outcomes had to be described in detail, with cognitive decline assessed using standard and full-scaled cognitive tests, and dementia or AD diagnosed using objective and globalized diagnostic criteria such as Diagnostic and Statistical Manual of Mental Disorders criteria, International Classification of Diseases codes, and National Institute of Neurological and Communicative Disorders and Stroke and the Alzheimer’s Disease and Related Disorders Association criteria; and (3) For dose–response analysis, the pooled related factors needed to be separated into at least three levels, with specific or calculable person-years and case numbers within each level.

Two researchers independently screened the included studies. If a discrepancy arose, the third author was asked to determine whether to include or exclude the study. When two or more studies originated from the same database, the study with the biggest sample size and/or most detailed information was retained. Additionally, we combed through the bibliographies of qualified studies to avoid overlooking any potentially pertinent studies.

### Data extraction

Predesigned templates were used to extract data from each article, including first author, publication year, study design, population resource, cognitive status at the baseline, mean age, gender composition, follow-up duration, attrition rate, total sample size for analysis, incident case, type of outcome, outcome measurement, type of exposed factor, measurement of exposed factor, adjusted confounders, and risk estimates. If any required data was not reported in the publication, we contacted the authors to obtain it. Two authors with extensive experience extracted the data, and any disagreements were resolved with assistance from a third reviewer.

### Assessment of study quality

The Newcastle–Ottawa Scale (NOS) was used to evaluate potential bias. In order to more accurately measure potential bias in studies, a modified version of the NOS was utilized [12] (Additional file 1 Appendix A and Appendix B). The NOS can fully evaluate a single study, incorporating various criteria such as representativeness, comparability, objectivity, and reliability (Additional file 1: Appendix C).

### Statistical analyses

Above all, the risk estimates and 95% confidence intervals (CIs) for a series of risk factors were acquired for further analysis. When odds ratios (ORs) were provided in some articles instead of relative risks (RRs) or hazard ratios (HRs), we used the following algorithm to convert ORs to RRs [13]:

$$RR\;adjusted=OR\;adjusted/\lbrack\left(1-P_0\right)+\;\left(P_{0\;}\ast\;OR\;adjusted\right)\rbrack$$

*P*_0_ represents the incidence of dementia or cognitive decline in the non-exposed group. If *P*_0_ cannot be calculated, the overall incidence rate of the entire sample can be used instead.

To begin, we specified definitions of exposures to facilitate comparison of pooled data across studies: (a) early menarche was defined as age at menarche ≤ 13 years; (b) late menarche was ≥ 16 years; (c) early menopause was ≤ 45 years; (d) late menopause was ≥ 54 years; (e) short reproductive period was ≤ 34 years; (f) long reproductive period was ≥ 38 years; (g) early childbearing was ≤ 20 years; and (h) late childbearing was ≥ 30 years. The fixed model combined risk estimates of the same category within a study, while the random model pooled estimates across studies [13]. Heterogeneity was evaluated by the Q test and quantified by the *I*^2^ metric [14]. For factors with ≥ 10 studies, subgroup analysis and meta-regression were conducted.

Dose–response analysis for eligible components was undertaken using the inverse variance weighted least squares regression with cluster robust error variances (REMR model) [15]. For studies that did not use the lowest category as the reference group, we reassigned the reference group and recalculated the effect sizes using Orsini’s method [12]. When a range was provided, the midpoint represented the average exposure level. For open-ended categories, the exposure level was set to the boundary limit plus/minus the interval length of adjacent groups [16]. Figures and analyses were performed using GraphPad Prism 9.0 and Stata Version 12.0.

### Evaluation of evidence certainty

#### GRADE scores

Five domains were used to assess the credibility of the meta-analysis: “risk of bias [17],” “inconsistency [18],” “imprecision [19],” “indirectness [20],” and “publication bias [21].” The certainty of each domain was classified as “0 (probably high), − 1 (probably moderate), or − 2 (probably low).” The Grading of Recommendations Assessment, Development, and Evaluation (GRADE) approach evaluated the overall credibility of the meta-analysis [22] (website of the GRADE: https://community.cochrane.org/help/tools-and-software/gradepro-gdt) (Additional file 1: Appendix D).

#### Systematic review index

Unlike traditional studies which neglected research not suitable for meta-analysis, we introduced a new parameter called “index S.” Index S was calculated using the following formula:

$$Index\;S\;=\left[\left(NOS\;score\;\left[study\_1\right]\;/9\right)\ast P\;+\;\left(NOS\;score\;\left[study\_2\right]\;/9\right)\ast P+...+\;\left((NOS\;score\;\left[study\_N\right]/9\right)\right]/N.$$

*N* denoted the total number of studies included in the systematic review. To better describe the results, we calculated both “index S_for_” and “index S_against_.” Index S_for_ reflected the number of high-quality studies agreeing with the meta-analysis results, while index S_against_ reflected those disagreeing. When calculating index S_for_, *P* was 0 if the study results were inconsistent with the meta-analysis, and 1 if consistent. The calculation of index S_against_ was reversed. Based on this, we introduced the concept of “index S divergence,” calculated as index S_for_ minus index S_against_. A higher divergence score indicated greater support for the meta-analysis result [23].

## Results

### Literature search results

**Fig. 1:**
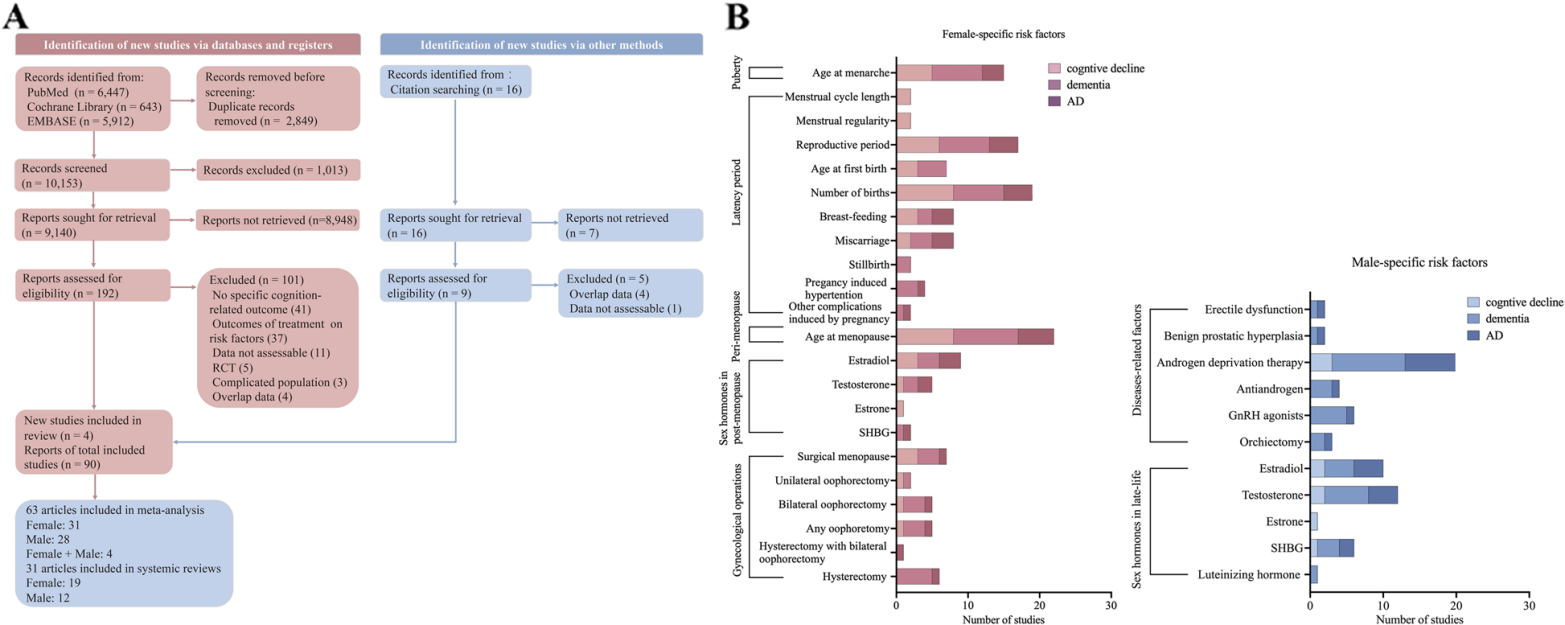
Flowchart of identifying included studies and characteristics of the factors. A total of 94 articles were finally recognized after screening 10,153 studies according to standard procedures. Among them, 63 articles were included in the meta-analysis, while 31 were in systematic reviews (**A**). Factors identified in females were classified into 5 parts according to different stages across the lifespan, (1) puberty-related factors; (2) latency period-related factors; (3) peri-menopause-related factors; (4) sex hormones in post-menopause; and (5) gynecological operations across the lifespan. Male-specific risk factors are sorted into 2 parts, (1) male-specific disease or disease-related factors and (2) sex hormones in late-life. Three outcomes were concluded: cognitive decline, dementia, and AD (**B**). Abbreviations: AD: Alzheimer’s disease, SHBG: sex hormone–binding globulin

Figure 1A depicts the process of selecting literature. After evaluating 10,153 publications, a total of 94 studies were identified, with 9,839,964 females and 3,436,520 males. The included studies consisted of 82 cohorts and 12 case–control studies (all case–control studies were included in analyses of female reproductive factors). Of these, 68 of them were from the community and 26 were from the hospital. Basic study information was provided in Additional file 1: Appendix E. Additional file 1: Appendix F summarizes the outcome measurements used in the included studies. Additional file 1: Appendix G contains the risk estimates from each study for each sex-specific reproductive factor.

Female-specific reproductive factors were classified into 5 categories according to life stages: (1) puberty-related factors, (2) latency period-related factors, (3) peri-menopause-related factors, (4) sex hormones in post-menopause, and (5) gynecological operations across the lifespan. Male-specific reproductive factors were sorted into 2 categories: (1) male-specific diseases and related factors and (2) late-life sex hormones. Figure 1B summarizes the number of included studies investigating each risk factor in relation to three outcomes: cognitive decline, dementia, and AD (Fig. 1).

### Female-specific reproductive risk factors for dementia or cognitive decline

**Fig. 2:**
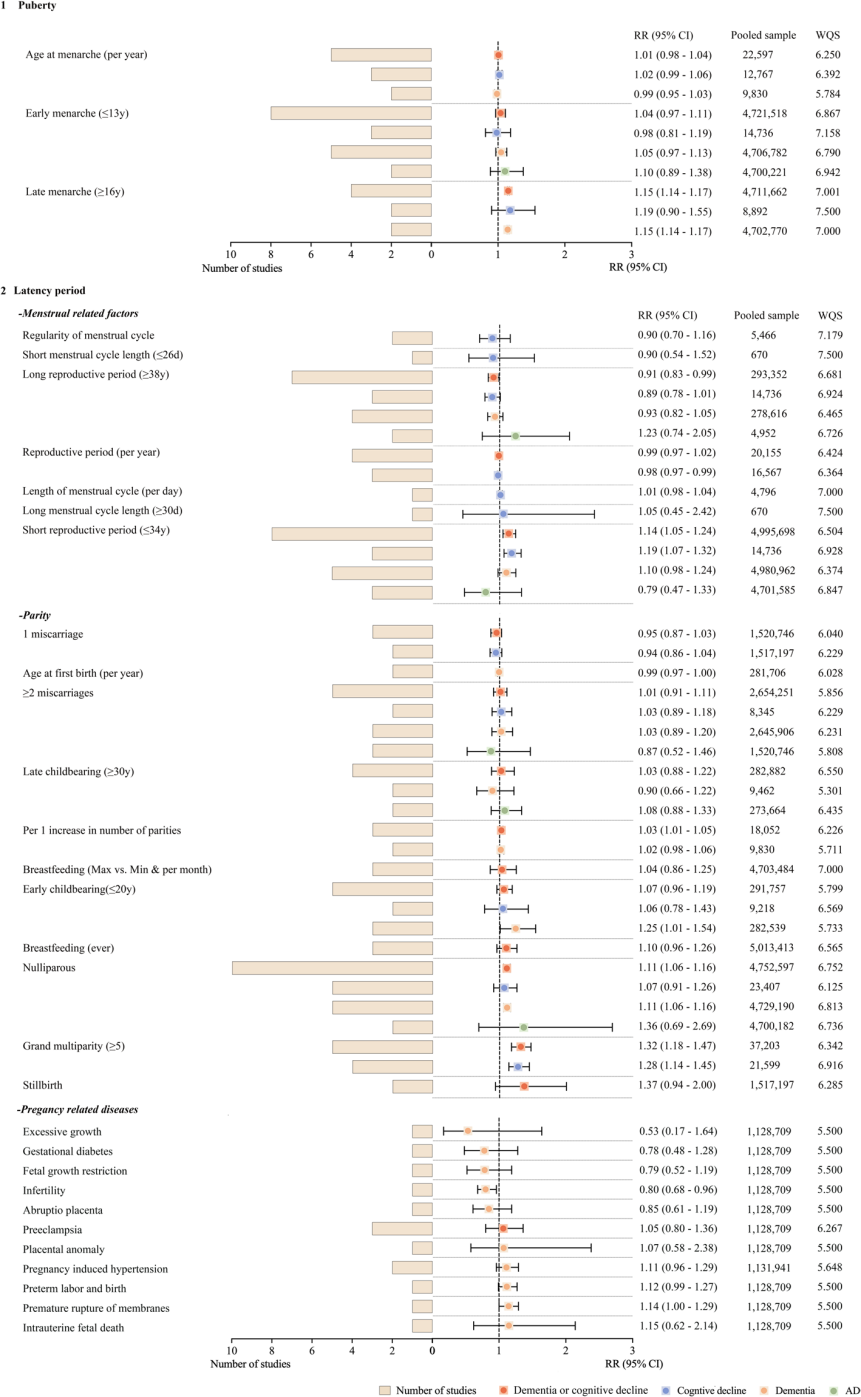
Associations of puberty and latency with risk of dementia or cognitive decline. Factors included in puberty were menarche-related (1), while factors included in the latency were further classified into 3 parts, named (1) menstrual-related factors, (2) parity-related factors, and (3) pregnancy-related diseases (2). They are listed in the order of RR values on the main outcome from least to most. The patchwork patterns by circle and square represent RR, and the horizontal lines across the spot represent 95% CI. Abbreviations: CI: confidence interval, RR: relative risk, WQS: weighted-NOS score

In puberty, late menarche could elevate the risk of dementia or cognitive decline by nearly 15% (RR = 1.15, 95% CI = 1.14 to 1.47, pooled studies = 4, *I*^2^ = 0%). In the latency period, the pooled RRs were 1.14 (95% CI = 1.05 to 1.24, pooled studies = 8, *I*^2^ = 72.3%) for the short reproductive period and 0.91 (95% CI = 0.83 to 0.99, pooled studies = 7, *I*^2^ = 0%) for the long reproductive period. For parity, increased risks were seen for both nulliparous (RR = 1.11, 95% CI = 1.06 to 1.16, pooled studies = 10, *I*^2^ = 0%) and grand parity (≥ 5) (RR = 1.28, 95% CI = 1.15 to 1.44, pooled studies = 4, *I*^2^ = 0%), with a 3% increase per parity (95% CI = 1.15 to 1.44, pooled studies = 3, *I*^2^ = 0%). One study reported a decreased risk of dementia with infertility (RR = 0.80, 95% CI = 0.68 to 0.96) (Fig. 2).

**Fig. 3:**
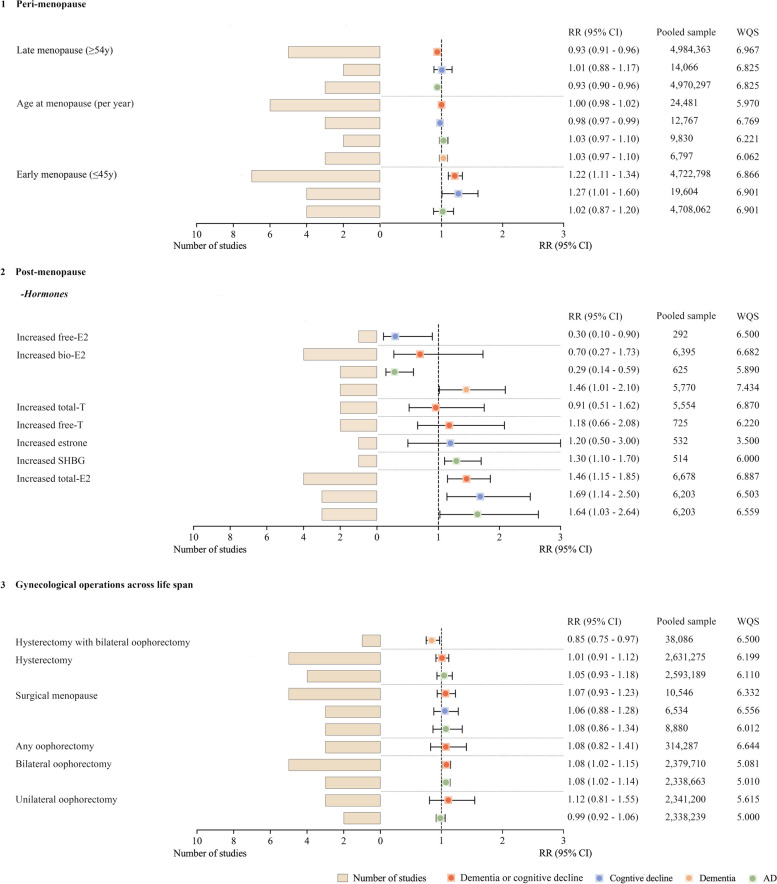
Associations of peri-menopause, post-menopause, and gynecological operations with risk of dementia or cognitive decline. Factors included in peri-menopause were menopause-related (**1**), and factors included in post-menopause were sex hormones in the elderly female (**2**). A total of 5 factors were included in the gynecological operations (**3**). All factors are listed in the order of RR values on the main outcome from least to most. The patchwork patterns by circle and square represent RR, and the horizontal lines across the spot represent 95% CI. Abbreviations: CI: confidence interval, E2: estradiol, RR: relative risk, SHBG: sex hormone–binding globulin, T: testosterone, WQS: weighted-NOS score

In the peri-menopause period, early menopause was associated with a 22% higher risk of dementia or cognitive decline (RR = 1.22, 95% CI = 1.11 to 1.34, pooled studies = 7, *I*^2^ = 68.5%), while late menopause was protective, lowering risk by 7% (RR = 0.93, 95% CI = 0.91 to 0.96, pooled studies = 5, *I*^2^ = 0%). Dose–response analysis showed an inverse linear relationship between age at menopause and dementia/cognitive decline (*p* < 0.0005) (Additional file 1: Appendix H). For postmenopausal hormone levels, pooled RR was 1.46 (95% CI = 1.15 to 1.85, pooled studies = 4, *I*^2^ = 0%) for increased total estradiol. Findings from an individual study suggested that increased free estradiol levels could reduce the risk of cognitive decline (RR = 0.30, 95% CI = 0.10 to 0.90). Higher sex hormone–binding globulin (SHBG) levels increased the risk of dementia in one study (RR = 1.30, 95% CI = 1.10 to 1.70). For gynecological surgery, five studies on bilateral oophorectomy gave a pooled adjusted RR of 1.08 (95% CI = 1.02 to 1.15, *I*^2^ = 0), while one study on hysterectomy with bilateral oophorectomy had a RR of 0.85 (95% CI = 0.75 to 0.97) (Fig. 3).

### Male-specific reproductive risk factors for dementia or cognitive decline

The pooled RRs for dementia or cognitive decline with ADT were 1.18 (95% CI = 1.08 to 1.29, pooled studies = 13, *I*^2^ = 94.5%) compared to prostate cancer patients not receiving ADT, and 1.17 (95% CI = 1.09 to 1.25, pooled studies = 2, *I*^2^ = 68.7%) versus those without prostate cancer. By ADT type, RRs versus prostate cancer controls were 2.03 for antiandrogens (95% CI = 1.76 to 2.35, pooled study = 1) and 1.47 for orchidectomy (95% CI = 1.11 to 1.95, pooled study = 1). Versus healthy controls, RRs were 1.60 for orchidectomy (95% CI = 1.32 to 1.93, pooled study = 1) and 1.15 for gonadotropin-releasing hormone (GnRH) agonists (95% CI = 1.07 to 1.23, pooled study = 1). Subgroup analysis was conducted by race, follow-up time, study quality, and sample size (Additional file 1: Appendix I). Meta-regression analysis was performed on the pooled sample sizes, mean follow-up years, years of publication, and mean ages, but the degree of heterogeneity among the aforementioned factors could not be distinguished. Dose–response analysis showed an increased risk of dementia or cognitive decline with longer ADT duration (Additional file 1: Appendix H).

**Fig. 4:**
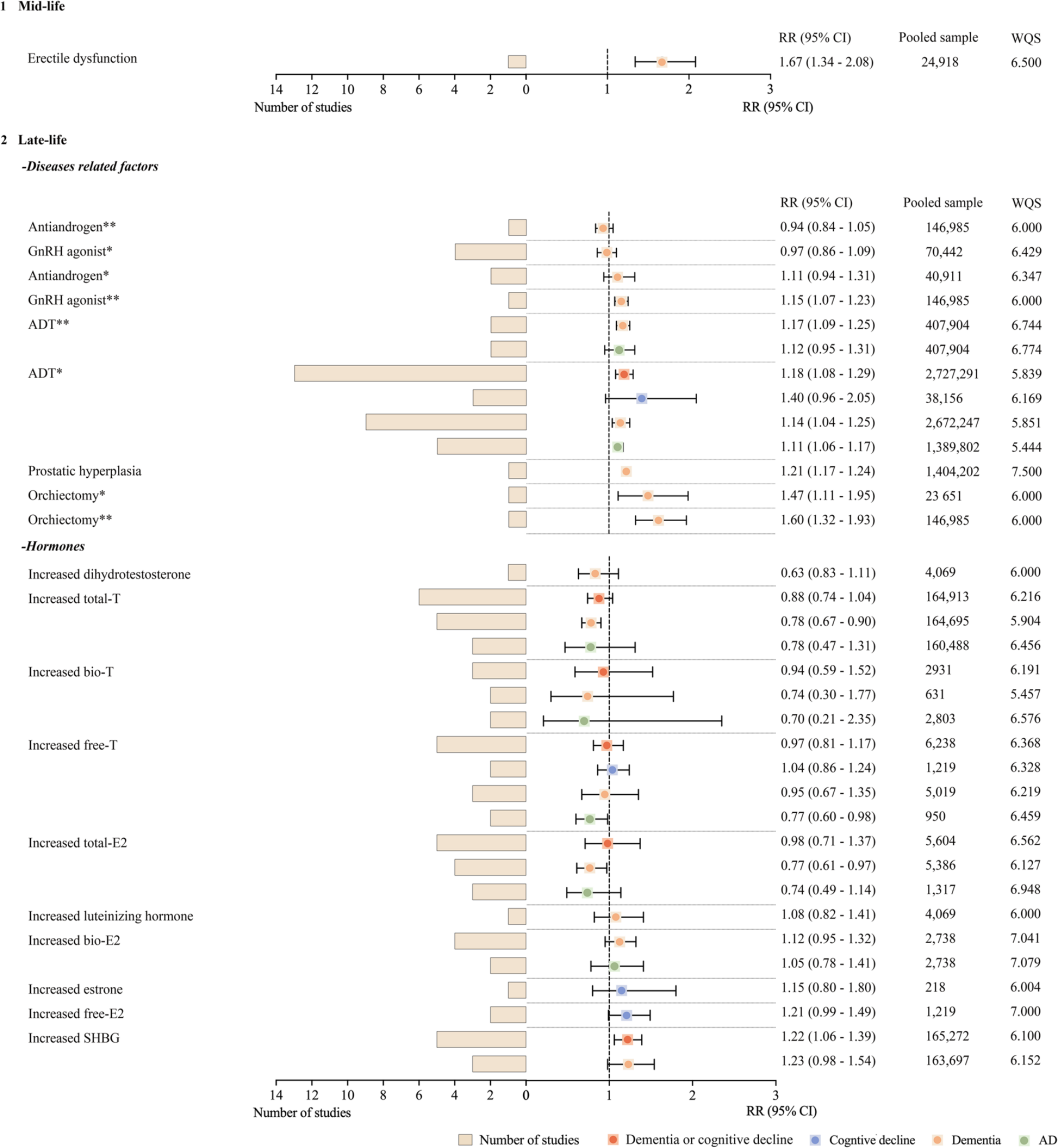
Male-specific risk factors of dementia or cognitive decline. The factor included in the middle-aged was ED (**1**), and factors included in the elderly were sorted into disease-related factors and sex hormones in the elderly (**2**). They are listed in the order of RR values on the main outcome from least to most. The patchwork patterns by circle and square represent RR, and the horizontal lines across the spot represent 95% CI. Abbreviations: ADT: androgen deprivation therapy, CI: confidence interval, E2: estradiol, GnRH: gonadotropin-releasing hormone, RR: relative risk, SHBG: sex hormone–binding globulin, T: testosterone, WQS: weighted-NOS score. * Refers to the results compared to prostate cancer patients without ADT, ** refers to the results compared to normal healthy people without prostate cancer

Erectile dysfunction (ED) (RR = 1.67, 95% CI = 1.34 to 2.08, pooled study = 1) and benign prostatic hyperplasia (BPH) (RR = 1.21, 95% CI = 1.17 to 1.24, pooled study = 1) were associated with increased risk of dementia. Additionally, higher SHBG levels were linked to an elevated risk of dementia or cognitive decline (RR = 1.22, 95% CI = 1.06 to 1.39, pooled studies = 5, *I*^2^ = 0%) (Fig. 4).

### Evidence-based rating of the results

Based on GRADE scores, all characteristics included in the meta-analysis were rated as a certainty of “extremely low” (Additional file 1: Appendix J). The low level of certainty was mainly driven by low scores on risk of bias, with 58% of studies graded as high concern. In addition, the limited indirectness and public of bias also contributed to the low GRADE scores.

### Systematic reviews

According to the results of index S divergence, 40% of female-specific reproductive factors had divergence > 50%. Ranging from − 44 to 72%, parity was the most controversial factor (index S divergence =  − 0.04). For male-specific reproductive factors, 28.5% had divergence > 50%, with ADT being the most disputed result (index S divergence =  − 0.02) (Additional file 1: Appendix K and Appendix L).

## Discussion

**Fig. 5:**
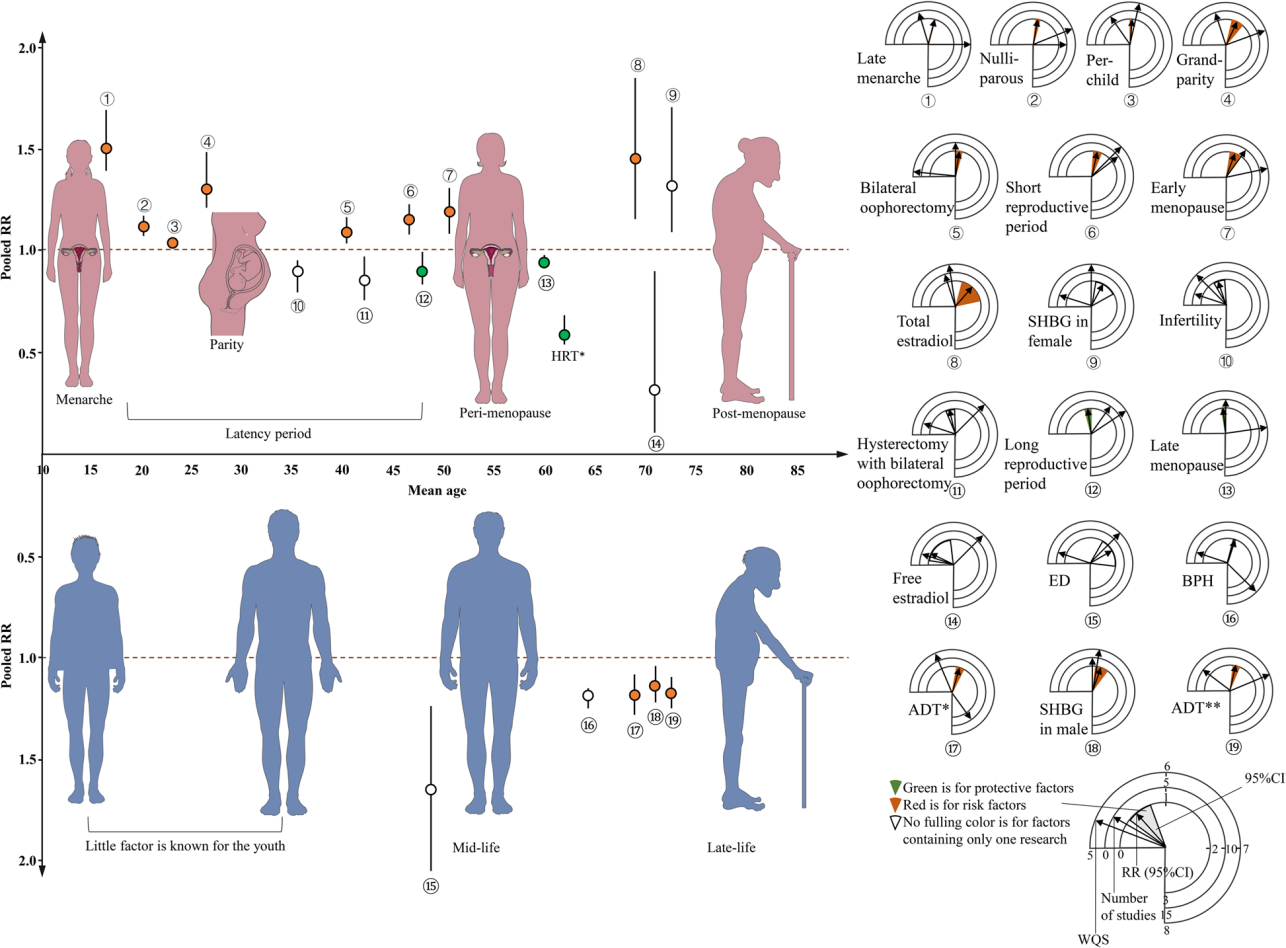
Summary findings of sex-specific risk factors across the lifespan for dementia or cognitive decline. Across the lifespan in females, meta-analyses indicated 8 risk factors across life stages associated with increased dementia or cognitive decline: late menarche, nulliparous, per child increase in parity, grand parity, bilateral oophorectomy, short reproductive period, early menopause, and higher total estradiol. Three protective factors were identified: long reproductive period, late menopause, and HRT. Individual studies also found higher SHBG increased risk, while infertility, oophorectomy with hysterectomy, and higher free estradiol decreased risk. Across the lifespan in males, meta-analyses showed ADT and higher SHBG increased dementia/cognitive decline risk. ED and BPH also elevated risk based on individual studies. The *X*-axis stands for the specific age at which the factor occurred or the average age of pooled studies. The *Y*-axis stands for the value of RRs. The colored spots represent the pooled RR from meta-analyses, while the uncolored spots indicate the RR from individual studies. Lines across the spot or rhombus represent the 95% CI. On the right edge of the figure are 19 clock plots. Each clock plot contains three concentric 3/4 circles, the innermost circle covers from − 1 to 2, it reflects RR and 95% CI, the arrow points at RR, and the colored range represents the pooled 95% CI from meta-analyses of more than 2 studies. Red ranges denote the factor as a risk, while green ranges indicate a protective effect. Unfilled ranges signify 95% CIs from individual studies. The middle circle represents the number of studies included for each factor, covering a range of 0–15. The outmost circle represents weigher NOS scores with a range of 5–8. The results will be more reliable when the arrows of the outer 2 circles point further to the right part. The results will be more significant when the arrows of the innermost circle point further to either the left (protective) or right (risk) part. Abbreviations: ADT: androgen deprivation therapy, BPH: benign prostate hyperplasia, CI: confidence interval, ED: erectile dysfunction, HRT: hormone replacement therapy, PCa, prostate cancer, RR: relative risk, SHBG: sex hormone–binding globulin, WQS: weighted-NOS score. * The result of HRT was indicated by E S LeBlanc

This systematic review and meta-analysis identified sex-specific reproductive factors associated with dementia or cognitive decline. In females, an increased risk of dementia or cognitive decline was associated with late menarche, nulliparity, per-child increase in parity, short reproductive period, early menopause, bilateral oophorectomy, and higher total estradiol levels. In males, increased risk was associated with ADT and higher SHBG levels (Fig. 5). Based on these findings, we hypothesized that sex-specific reproductive risk factors could lead to dementia or cognitive decline in a hormone-motivated way.

Our findings suggest estrogen may be beneficial for cognition. First, results for menstrual factors showed an increased risk of dementia or cognitive decline with late menarche, short reproductive period, and early menopause—reflecting low lifetime estrogen exposure. This aligns with previous research by Fu demonstrating a lower risk of dementia with later menopause and a longer reproductive period [24]. However, Georgakis found later menopause and longer reproductive period did not affect the risk of dementia, only slowing the progression of cognitive impairment [25]. This discrepancy could be due to differing category definitions. For example, we defined “late menopause” as ≥ 54 years, while Fu used ≥ 45 years. Notably, there was no uniform classification in the Georgakis study.

Second, our results indicated grand multiparity (≥ 5) and even each additional birth may impair cognition. Compared to nulliparous women, parous women have approximately 22% lower estrogen levels and shorter menstrual cycle lengths [26], reflecting reduced cumulative estrogen exposure.

Third, bilateral oophorectomy can cause a sudden drop in endogenous estrogen, which we found to be associated with an increased risk of dementia or cognitive decline [27]. This aligns with prior research showing a higher risk of dementia in those undergoing bilateral oophorectomy before 45 years [28]. Further, an earlier age at bilateral oophorectomy was linked to a greater risk of dementia [27].

Fourth, one study found that increased serum-free estradiol was associated with reduced risk of cognitive decline, while higher SHBG (which binds and decreases free estradiol) increased risk. This supports the findings above. However, we found higher total estradiol was associated with an increased risk of dementia or cognitive decline, aligning with one study concluding higher total estradiol independently predicts incident dementia [29]. Furthermore, they hypothesized that higher estradiol levels could contribute to atherothrombosis, thereby increasing the risk of dementia in a vascular-dependent manner.

Estrogen plays an important role in the nervous system and benefits cognition. It protects the brain from cognitive decline through several mechanisms, including promoting neurogenesis and reducing β-amyloid (Aβ) and hyperphosphorylated tau, thereby slowing cognitive deterioration [30]. Additionally, a recent study found physiological estradiol levels inhibited the synthesis of complement component 3 (C3) nitrite, suggesting estradiol may protect women from C3 protein S-nitros(yl)ation (SNO) effects. However, as estrogen levels decrease after menopause, SNO levels at C3 increase, resulting in heightened synaptic phagocytosis, synapse loss, and subsequent cognitive decline [31]. Although estrogen benefits cognition and HRT was found to ward off cognition deterioration and associated with larger brain volume in women with AD risk [32], HRT is still not recommended for the primary prevention of chronic conditions in postmenopausal women, according to the US Preventive Services Task Force (USPSTF) [33].

Similar to the influence of estrogen on female cognition, androgens appear to play a comparable role in males. Our findings suggest ADT, which suppresses androgen levels to treat prostate cancer, may increase the risk of dementia or cognitive decline. These findings are consistent with previous study [34]. We also identified two studies using healthy controls without prostate cancer as the comparison, which showed similar results except for different subtype findings. The S divergence reflects controversy on this topic. We posited the divergence may stem from systematic reviews mostly examining “cognitive decline” while meta-analyses focused on “dementia” as the outcome. Further analysis is needed to elucidate the differences.

Moreover, we also found ED associated with an increased risk of dementia versus healthy males, which was coordinated with another cross-sectional study [35]. ED is closely tied to low androgen levels [36]. In addition, higher SHBG levels were found to elevate the risk of dementia in men, consistent with a previous study [37]. Increased serum SHBG levels can decrease bioactive androgen levels. Like estrogen, androgens benefit male cognition by promoting neuronal growth and axonal regeneration, and modulating Aβ accumulation [38]. Additionally, decreased androgen could affect cognition by modifying the hypothalamic connection via the hypothalamus-pituitary–gonadal axis [39].

Our study has several notable strengths compared to previous studies. First, the key strength of our work is the intensive literature search, screening, and extraction. This study is the first systematic review and meta-analysis focusing on the associations between sex-specific reproductive risk factors and dementia or cognitive decline. Second, risk factors were carefully characterized to unify results across diverse studies with precision. Third, the index S divergence concept was introduced to assess heterogeneity more credibly. Fourth, dose–response relationships were explored for multiple factors. Finally, a multifaceted approach rated the strength of the evidence.

This study has some limitations to note. First, while a full range of sex-specific reproductive factors was identified, the number and heterogeneity of studies varied across risk factors. Second, dementia subtypes could not be thoroughly examined due to insufficient studies. Third, included studies encompassed retrospective and case–control designs in addition to prospective cohorts. Further large-scale longitudinal studies are needed to provide more definitive evidence.

## Conclusions

In summary, this systematic review and meta-analysis comprehensively examined associations between sex-specific reproductive factors and dementia or cognitive decline. The findings support hormone-related mechanisms underlying links between reproductive factors and cognition. Further high-quality research is needed to elucidate the role of individual risk factors and enable precise, personalized approaches to dementia prevention.

### Supplementary Information

**Additional file 1: Appendix A.** Newcastle–Ottawa quality assessment scale-cohort studies. **Appendix B.** Newcastle–Ottawa quality assessment scale-case control studies. **Appendix C.** Quality assessment according to the Newcastle–Ottawa Scale of cohort studies and case–control studies. **Appendix D.** Grading approaches used to assess the credibility of meta-analysis. **Appendix E.** Characteristics of included studies. **Appendix F.** Measurement of the outcomes on included studies. **Appendix G.** Characteristics of studies with cognitive decline, dementia, and AD. **Appendix H.** Results of dose–response analysis between certain factors and relative risks of dementia or cognitive decline. **Appendix I.** Results of subgroup analysis on ADT. **Appendix J.** Detailed GRADE score on credibility of the meta-analysis on dementia or cognitive decline. **Appendix K.** Results of systemic review. **Appendix L.** Results of index S divergence.

## Data Availability

Not applicable.

## Abbreviations

AD: Alzheimer’s disease
ADT: Androgen deprivation therapy
Aβ: β-Amyloid
BPH: Benign prostatic hyperplasia
C3: Complement component 3
CIs: Confidence intervals
ED: Erectile dysfunction
GnRH: Gonadotropin-releasing hormone
HRs: Hazard ratios
HRT: Hormone replacement treatment
NOS: Newcastle-Ottawa Scale
ORs: Odds ratios
RRs: Relative risks
SHBG: Sex hormone–binding globulin
