# Supplementary material for: Male- and female-specific reproductive risk factors across the lifespan for dementia or cognitive decline: a systematic review and meta-analysis
Source: BMC Med. 2023 Nov 23;21:457. doi: 10.1186/s12916-023-03159-0 (PMC10666320; doi:10.1186/s12916-023-03159-0)

Male- and female-specific reproductive risk factors across the lifespan for dementia or cognitive decline: A systematic review and meta-analysis

Appendix A Newcastle-Ottawa quality assessment scale-cohort studies…..……………………………………………..…...…………………………1

Appendix B Newcastle-Ottawa quality assessment scale-case control studies…….……………………………………………………………..….…4

Appendix C Quality assessment according to the Newcastle-Ottawa Scale of cohort studies and case-control studies. ……………………………………...……6

Appendix D Grading approaches used to assess the credibility of meta-analysis.……………………………………...……………………………...10

Appendix E Characteristics of included studies. ………………………………………………...……………………………………………..12

Appendix F Measurement of the outcomes on included studies…………………………………………………………………………………...30

Appendix G Characteristics of studies with cognitive decline, dementia, and AD…………………………………………...................................35

1.Characteristics of studies with cognitive decline, dementia, and AD by menstrual and parity ……………………………………………………...35

a. Characteristics of studies with cognitive decline, dementia, and AD by age at menarche…………………………………………………………35

b. Characteristics of studies with cognitive decline, dementia, and AD by length of menstrual cycle………………………………………………..38

c. Characteristics of studies with cognitive decline, dementia, and AD by menstrual regularity………………………………………………………… 39

d. Characteristics of studies with cognitive decline, dementia, and AD by age at menopause………………………………………………………… 40

e. Characteristics of studies with cognitive decline, dementia, and AD by reproductive period……………………………………………………… 46

f. Characteristics of studies with cognitive decline, dementia, and AD by age at first birth…………………………………………………………….49

g. Characteristics of studies with cognitive decline, dementia, and AD by parity………………………………………………………………….……51

h. Characteristics of studies with cognitive decline, dementia, and AD by miscarriage and stillbirth……………………………………………… 55

i. Characteristics of studies with cognitive decline, dementia, and AD by breastfeeding…………………………………………………………….57

2.Characteristics of studies with cognitive decline, dementia, and AD by gynecologic surgery……………………………………………………...59

a. Characteristics of studies with cognitive decline, dementia, and AD by surgical menopause………………………………………………………59

b. Characteristics of studies with cognitive decline, dementia, and AD by oophorectomy, hysterectomy, and hysterectomy with bilateral oophorectomy….60

3.Characteristics of studies with cognitive decline, dementia, and AD by pregnancy-related diseases……………………………………….……..63

4.Characteristics of studies with cognitive decline, dementia, and AD by sex hormones in female……………………………………………………64

a. Characteristics of studies with cognitive decline, dementia, and AD by estradiol in female…………………………………………………………64

b. Characteristics of studies with cognitive decline, dementia, and AD by testosterone in female……………………………………………………67

c. Characteristics of studies with cognitive decline, dementia, and AD by estrone and SHBG in female……………………………………..……………...69

5.Characteristics of studies with cognitive decline, dementia, and AD by male-specific diseases……………………………………………………………70

6.Characteristics of studies with cognitive decline, dementia, and AD by sex hormones in male………………………………………………………………76

a. Characteristics of studies with cognitive decline, dementia, and AD by estradiol in male……………………………………………………………....76

b. Characteristics of studies with cognitive decline, dementia, and AD by testosterone in male……………………………………………………………79

c. Characteristics of studies with cognitive decline, dementia, and AD by estrone, SHBG, LH, dihydrotestosterone in male………………………………...83

Appendix H Results of dose-response analysis between certain factors and relative risks of dementia or cognitive decline……………………………………….85

Appendix I Results of subgroup analysis on ADT…………..…………………………………………………………………………………………………...…87

1. Subgroup analysis between races…………………………………………………………...………………………………………………………………….87

2. Subgroup analysis between mean follow-up years…...……………………………………...……...………………...……………………………………..88

3. Subgroup analysis between sample sizes…………………………………………………...……………………………………………………………..89

4. Subgroup analysis between NOS scores……………...……………………………………...……...……………………………………………...………..90

Appendix J Detailed GRADE score on credibility of the meta-analysis on dementia or cognitive decline…………………………………………………………91

Appendix K Results of systemic reviews……………………………………………………………………………………………………95

1. Results of systemic reviews in female-specific reproductive factors……………………………………………………………95

2. Results of systemic reviews in male-specific reproductive factors………………………………………………………………99

Appendix L Results of index S divergence………………………………………………………………………………………………101

1. Results of index S divergence in female-specific reproductive factors…………………………………………………………101

2. Results of index S divergence in male-specific reproductive factors……………………………………………………………102

Appendix A Newcastle-Ottawa quality assessment scale-cohort studies.

Risk of bias	Questions	Scores	Standards
Selection (generalizability, assessment bias and potential reverse causality)	Q1. representativeness of the exposed cohort	☆	a) randomly selected or<br>b) database covering very large population or<br>c) participation rate (PR) is ≥ 90% or<br>d) reported there is no difference in important characteristics between those who agreed to participate and those who did not
		0.5☆	PR varies from 70% to 90% with no reporting of significant difference in important characteristics between those who agreed to participate and those who did not
		0	Selected group of users e.g. nurses, volunteers or no description
	Q2. selection of the non-exposed cohort	☆	Drawn from the same community as the exposed cohort
		0	Drawn from a different source or no description of the derivation of the non-exposed cohort
	Q3. ascertainment of exposure	☆	Questionnaire or interview based on self-report to series questions or database
		0.5☆	Self-report to simple question with potential recall bias
		0	No description
	Q4. demonstration that outcome of interest was not present at baseline	☆	Cognitively intact for outcome as dementia or MCI; Free of dementia for population with MCI at baseline
		0.5☆	Free of dementia (cognitively intact & cognitive impairment no dementia (CIND)) for outcome of dementia
		0	No description
Confounding bias	Q5. comparability of cohorts on the basis of the design or analysis	☆☆	Except for age, sex, and education, the analysis still controls forat least another two domains of AD risk factors, including *APOE4*, pre-existing disease, lifestyle, medical exposure, biochemical exposure, occupation, diet, etc.
		☆	Controls for age, sex and education
		0	No description
Outcome (assessment	Q6. assessment of outcome	☆	Independent or blind assessment

bias and attrition bias)		0.5☆	Record linkage (e.g. identified through ICD codes on database records or claim data)
		0	Self-report or no description
	Q7. follow-up long enough for outcomes to occur? #	☆	The average or max duration reached the lower 95% CI.
		0	The average or max duration did not reach the lower 95% CI.
	Q8. adequacy of follow up of cohorts*	☆	Attrition rate ≤ 5%
		0.5☆	5% ≤ Attrition rate ≤ 20%
		0	Attrition rate > 20% and no description of those lost or no description

A maximum of two stars can be given for Comparability. In the involving version, an assignment of a half point (0.5) is permitted.

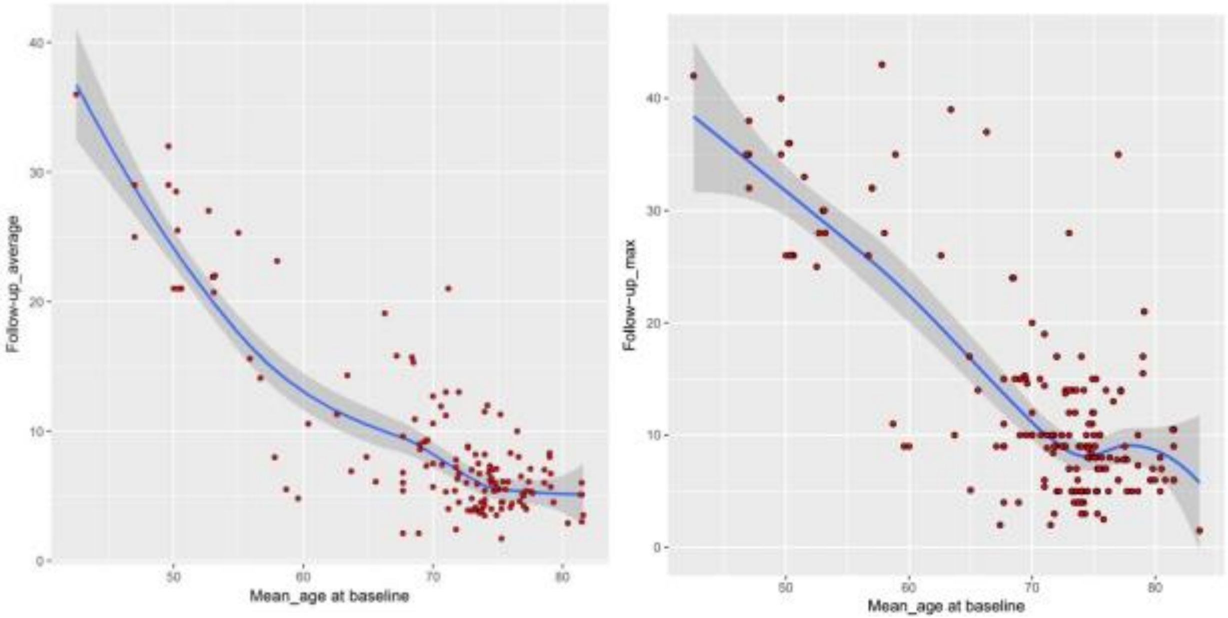

#Figure: It is obviously absurd to define a common period for population with diverse age range at baseline. A presumable negative correlation was reasonably supposed to exist between so-called adequate follow-up period and average age of population at baseline. Here, we will draw the nonlinear regression line with its 95% confidence

interval (CI) for the association between the mean/max follow-up duration and mean age of population at baseline for AD cohorts (unpublished data). We will predefine that the follow-up is adequate if the average or max duration reach the lower 95% CI.

*It has been indicated that a rate of loss < 5% probably leads to little bias, whereas a rate of loss that is greater than 20% potentially poses serious threats to validity.

Appendix B Newcastle-Ottawa quality assessment scale-case control studies.

Risk of bias	Questions	Scores	Standards
**Selection**	Q1. Is the case definition adequate?	☆	Yes, with independent validation
		0.5☆	Yes, eg record linkage or based on self-reports
		0	No description
	Q2. Representativeness of the cases	☆	Consecutive or obviously representative series of cases
		0	Potential for selection biases or not stated
	Q3. Selection of Controls	☆	Community controls
		0.5☆	Hospital controls
		0	No description
	Q4. Definition of Controls	☆	No history of disease (endpoint)
		0	No description of source
**Comparability**	Q5. Comparability of cases and controls on the basis of the design or analysis	☆	Study controls for _______________(Select the most important factor.) *
		☆	Study controls for any additional factor *
		0	No description
**Exposure**	Q6. Ascertainment of exposure	☆	a) secure record (eg surgical records)<br>b) structured interview where blind to case/control status
		0.5☆	a) interview not blinded to case/control status<br>b) medical record only
		0	Self-report or no description
	Q7. Same method of ascertainment for cases and controls	☆	Yes
		0	No
	Q8. Non-Response rate	☆	Same rate for both groups
		0	Non respondents described

Rate different and no designation

Appendix C Quality assessment according to the Newcastle-Ottawa Scale of cohort studies and case-control studies.

Author, Year	Q1	Q2	Q3	Q4	Q5	Q6	Q7	Q8	SUM
Mirjam I. Geerlings, 2002	1	1	1	0.5	2	1	1	0	7.5
Majon Muller, 2008	0	1	1	1	2	1	1	0	7
Ellika Andolf, 2020	1	1	1	0	2	0.5	0	0	5.5
Chun-Ming Yang, 2015	1	1	1	1	2	0.5	0	0	6.5
Vanessa Sánchez-Martínez, 2021	0	1	1	0	0	1	0.5	0	3.5
M Nelander, 2017	1	1	1	1	1	0.5	1	0	6.5
Yoko Shimizu, 2019	0	1	1	1	2	1	1	0.5	7.5
Kristine Yaffe, 2010	0	1	1	0.5	2	1	1	0	6.5
W.A. Rocca, 2007	1	1	1	1	1	1	1	0	7
C.N. Lessov-Schlaggar, 2005	0	1	1	0	1	1	1	0	5
Kohei Okamoto, 2015	0	1	1	0	0	1	0.5	0	3.5
Martin J. Prince, 2018	1	1	0.5	0.5	1	1	0	0.5	5.5
Elizabeth Barrette-Conner, 1999	0	1	1	0	2	1	0	0	5
S. D. Chung, 2016	1	1	1	0.5	1	0.5	1	0	6
Kevin T. Nead, 2016	1	1	1	0.5	0	0.5	1	0	5
Jung Eun Yoo, 2020	1	1	1	0.5	2	0.5	1	0	7
Hyesue Jang, 2018	1	1	1	1	0	0.5	1	0	5.5
Natalie L. Rasgon, 2005	1	1	1	1	1	0	1	0	6
Li-Ting Kao, 2017	1	1	1	0.5	2	0.5	1	0	7
Kevin T. Nead, 2016	1	1	1	0.5	2	0.5	1	0	7
Myungsun Shim, 2020	1	1	1	0.5	2	0.5	0	0	6
David Robinson, 2019	1	1	1	0.5	1	0.5	1	0	6
Joanne Ryan, 2009	0	1	1	0.5	1	1	0	0	4.5

Ineke R. Postma, 2016	0	1	1	0	0	1	1	0	4
Bulent Gunlusoy, 2016	0	1	1	1	0	1	1	0	5
Sanna L. Read, 2017	0	1	1	0	2	1	1	0	6
S.D. Moffat, 2004	0	1	1	0.5	2	1	1	0	6.5
Emily W. Harville, 2019	0	1	0.5	0	2	1	0	0	4.5
Majon Muller, 2010	0	1	1	0.5	2	1	0	0.5	6
Farzin Khosrow-Khavar, 2016	1	1	1	0.5	2	0.5	1	0	7
Gail A. Laughlin, 2010	0.5	1	1	0	1	1	1	0	5.5
Juan Morote, 2017	0	1	1	0	0	1	0	0	3
Xingyue Song, 2020	1	1	1	0.5	2	1	1	0	7.5
Alain K. Koyama, 2016	0	1	1	0	2	1	1	0	6
Leung-Wing Chu, 2010	0	1	1	0.5	2	1	0	0.5	6
Molly Fox, 2018	1	1	1	1	1	1	1	0	7
Supriya Gupta Mohile, 2010	0	1	1	0.5	0	1	0	0	3.5
Ellika G Andolf, 2007	1	1	1	0.5	2	1	1	0	7.5
Jenna Najar, 2020	1	1	1	0.5	2	0.5	1	0	7
Kristine Yaffe, 1998	0	1	1	0	0	1	0	0.5	3.5
Wen-Kuan Huang, 2020	1	1	1	0.5	1	0.5	1	0	6
Saima Basit, 2018	1	1	1	0.5	2	0.5	1	0	7
Mette Norgaard, 2021	1	1	1	1	2	0.5	1	0	7.5
Hung-Tse Chou, 2021	1	1	0.5	1	0	0.5	1	0	5
J Ryan, 2014	1	1	0.5	0.5	2	1	1	0	7
fu-Dong Li, 2020	1	1	0.5	1	0	0.5	1	0	5
Laure Carcaillon, 2014	1	1	1	0.5	2	1	0	0.5	7
Bum Sik Tae, 2018	1	1	1	1	0	0.5	1	0	5.5

Bushra Imtiaz, 2014	1	1	1	1	1	1	1	0	7
K. Yaffe, 2006	0	1	1	0	2	1	0	0.5	5.5
Thien Kieu Thi Phung, 2010	1	1	1	0.5	0	0.5	1	0	5
Julie A. Fields, 2017	0	1	0.5	0.5	0	0.5	1	0	3.5
Keiko Kurita, 2016	0	1	1	0	2	1	1	0	6
Sri Suravarapu, 2006	0	1	1	0.5	1	0.5	1	0	5
Ravishankar Jayadevappa, 2018	1	1	1	0.5	2	0.5	1	0	7
Paola Gilsanz, 2019	1	1	1	0.5	2	0.5	1	0	7
Onder Cinar, 2021	0	1	1	1	0	1	0	0.5	4.5
Joanne Ryan, 2012	0	1	1	0	1	1	1	0.5	5.5
Seo Hyon Baik, 2017	1	1	1	0.5	0	0.5	1	0	5
Mirjam I. Geerlings, 2001	0.5	1	0.5	0.5	2	0.5	1	0.5	6.5
Jong Bin Bae,2020	1	1	1	0	2	0.5	0	0	5.5
Michelle M. Mielke, 2016	0	1	1	0	2	1	0	0	5
L.-F. Low, 2005	1	1	1	1	0	1	1	0	6
Michelle Heys, 2010	1	1	1	0	2	0	0		5
Erin S. LeBlanc, 2010	0	1	1	1	2	1	0	0	6
Yasin Ceylan, 2019	0	1	1	1	0	1	1	0	5
Mirjam I. Geerlings, 2006	0.5	1	1	0.5	2	1	1	0	7
Laure Carcaillon, 2013	0	1	1	0.5	2	1	0	0.5	6
Shabbir M.H. Alibhai, 2016	0	1	1	1	1	1	1	0	6
Jihun Kang, 2020	1	1	1	0.5	2	0.5	1	0.5	7.5
Saima Basit, 2019	1	1	1	0.5	1	0.5	1	0	6
Riley Bove, 2014	0	1	1	0.5	1	0.5	1	0.5	5.5
A. Paganini-Hill, 2020	0	1	1	0.5	1	1	1	0	5.5

Sujarwoto Sujarwoto, 2019	1	1	1	1	0	1	1	0	6
Robert N. McLay, 2003	0.5	1	1	0	2	1	0	0	5.5
Carolien N.H. Abheiden, 2015	0	1	0.5	0	2	1	0	0	4.5
Jong Won Kim, 2021	1	1	1	1	1	0	1	0	6
Andrew H, 2018	1	1	1	0.5	0	0.5	0	0	4
Chiara Zucchella, 2012	0	1	1	0.5	2	0.5	1	0	6
Giovanni Ravaglia, 2007	1	1	0	1	1	1	1	0	6
Fu-Dong Li, 2015	0	1	1	0.5	2	1	0	0.5	6
Jessica Gong, 2022	1	1	1	1	0	1	1	0.5	6.5
Mirjam I. Geerlings, 2002	1	1	1	0.5	2	1	1	0	7.5
Changyong Yu, 2022	1	1	1	1	0	0	1	0	5
Margot J Overman, 2022	0	1	1	0	2	1	1	0.5	6.5
Alison Gemmill, 2022	1	1	0.5	0.5	0	1	1	0	5
N. Araújo, 2022	0	1	1	0	1	1	1	0	5
Joseph L. Saenz, 2021	0	1	0.5	0	2	1	1	0	5.5
Jui-Ming Liu, 2021	1	1	1	0.5	0	0.5	1	1	6
Onder Cinar, 2021	0	1	1	0	0	1	0	0	3
Lina Bergman, 2021	0	1	1	1	0	1	0	1	5
Hector Alonso-Quiñones, 2021	0	1	1	1	2	1	1	1	8
Karl H. Tully, 2019	0	1	1	0.5	0	0.5	1	1	5
Peter E. Lonergan, 2022	0	1	1	0.5	0	0.5	1	0	4
C. S. Uldbjerg, 2022	0	1	1	0.5	0	0.5	1	0	4

Appendix D Grading approaches used to assess the credibility of meta-analysis.

1. Risk of bias:

First, we calculated the weighted quality score (WQS)=QS (study 1) × weight% (study 1) + QS (study 2) × weight% (study 2) + ……QS (study n) × weight% (study n)

Note: "QS" means NOS score (total score= 9); "Weight" means weight value in the random model

WQS	Risk of bias
[7.5-9]	0 (probably low)
[6.5-7.5)	-1 (probably moderate)
<6.5	-2 (probably high)

2. **Inconsistency**

Estimate the heterogeneity by calculating the $\mathbf{I^2}$, p value, and τ^2.

$\mathbf{I^2}$	Heterogeneity level
<40%	0 (probably low)
40-60%	-1(probably moderate)
60-100%	-2 (probably high)

3. Imprecision

Calculate the RR & 95% confidence interval (CI) and 95% prediction interval (PI):

- The CI in a random-effects model contains highly probable values for the summary (mean)effect but gives no information on the range of true effects that are likely to be seen in other settings which convey what range of intervention effects are likely to be seen in other individuals.
- A 95%PI estimates where the true effects are to be expected for 95% of similar (exchangeable) studies that might be conducted in the future. In the absence of between-study heterogeneity, the prediction interval coincides with the respective CI. However, in case of heterogeneity, a prediction interval covers a wider range than a CI.

95%CI	95%PI	Rating
Not containing RR=1	Not containing RR=1	0
Containing RR=1 & not containing RR=0.75 or 1.25	Containing RR=1 & not containing RR=0.75 or 1.25	0
Not containing RR=1	Containing RR=1	-1
Containing RR=1 & not containing RR=0.75 or 1.25	Containing RR=1 & RR=0.75 or 1.25	-1

Containing RR=1 & 0.75 or 1.25	Containing RR = 1 & 0.75 or 1.25 or PI not available	-2

Note: RR=0.75 or 1.25 is representative of the rough cutoff of evident benefits or harm.

Further, this item is downgraded when the sample size is too small.

95%CI	Rating
Not containing RR=1 & not containing RR=0.75 or 1.25	0
Containing RR=1 & not containing RR=0.75 or 1.25	0
Not containing RR=1 & containing RR=0.75 or 1.25	-1
Containing RR=1 & 0.75 or 1.25	-2

4. Publication bias

Number of studies included	P value	Rating
>=10	Non-significant	0
<10	Non-significant	-1
>=10 or <10	Significant	-2

5. Indirectness

Outcome	Rating
Dementia or AD	0
Dementia or cognitive decline	-1
Cognition	-2

Appendix E Characteristics of included studies.

Author, year	Study design	Population source	Mean age (years)	Observation (years)	Sex	Incident cases	Outcome	Description of risk factor	Adjustment factor
Mirjam I. Geerlings, 2002	cohort	community	>55	6.3 (0–9)	female (53%), male (47%)	185	dementia AD	estradiol	women: adjusted for age, education, body mass index, smoking status, type of menopause, age at natural menopause, and ever use of hormonal replacement therapy. men: adjusted for age, education, body mass index, smoking status, and alcohol intake.
Majon Muller, 2008	cohort	community	77.4	4	male	44	cognitive decline	testosterone estradiol estrone	adjusted for age, BMI, cholesterol, intima media thickness, diabetes, hypertension, smoking, APOE-genotype, and socioeconomic status.
Ellika Andolf, 2020	cohort	community	＞25	＞20	female	ACD:6,881 AD:2,161	ACD	abruptio placenta placental anomaly recurrent miscarriages fetal growth restriction (NPR) excessive growth pregnancy induced hypertension preeclampsia preterm labor and birth PPROM gestational diabetes intrauterine fetal death infertility	adjusted for maternal education, parity, childlessness, origin, child composition, and the composite variable any cardiovascular disease (presence of either heart failure, heart disease, atherosclerosis, stroke, SLE, diabetes or hypertension).
Chun-Ming	cohort	community	NA	7	male	AD:328	AD	erectile dysfunction	adjusted for age, diabetes mellitus, hypertension,

Yang, 2015						Non-AD:94			coronary heart disease, chronic kidney disease, hyperlipidemia, stroke, depression, anxiety, thyroid disorder, geographic area, and income.
Vanessa Sánchez-Martínez, 2021	cohort	hospital	70.8	1	male	NA	cognitive decline	ADT	NA
M Nelander, 2017	cohort	community	71	8	female	197	dementia	hypertensive complication in pregnancy	adjusted for education, smoking and BMI.
Yoko Shimizu, 2019	cohort	community	72.9	10	female	227	cognitive decline	age at menarche (years)<br>menstrual regularity<br>menstrual cycle length (days)<br>age at menopause (years)<br>surgical menopause<br>reproductive period (years)<br>age at first birth (years)<br>number of births<br>breastfeeding	adjusted for age at examination (continuous), BMI (< 21.8, 21.8–24.1, ≥24.1 kg/m^2), educational background (junior high school, high school or higher), smoking (never, ever), leisure-time physical exercise (less than one day/week, one day/week or more), and past medical history (none or any of hypertension, diabetes mellitus, or depression).
Kristine Yaffe, 2010	cohort	community	＞70	6	female	37	cognitive decline	testosterone<br>estradiol	adjusted for age, education, body-mass index, current oestrogen use, surgical menopause, and baseline modified MMSE score.
W.A. Rocca, 2007	cohort	community	＞40	＞25	female	248	cognitive decline<br>dementia	unilateral oophorectomy<br>concurrent hysterectomy with hysterectomy<br>without hysterectomy<br>bilateral oophorectomy	adjusted for education (＜9; 9–12; 13–16; ＞16 years), history of depression (ever vs never), and type of interview (direct vs proxy).

C.N. Lessov-Schlaggar, 2005	cohort	community	63.1	10-16	male	NA	cognitive decline	testosterone<br>estradiol<br>estrone<br>SHBG	adjusted for age, education, and depressive symptomatology.
Kohei Okamoto, 2015	cohort	hospital	67.5	1	male	NA	cognitive decline	LH-RH agonist	NA
Martin J. Prince, 2018	cohort	community	72.0	3	female	692	dementia	age at menarche (per year)<br>age at menopause (per year)<br>reproductive period (per year)<br>parity (per child)<br>nulliparity<br>age at birth of first child	adjusted for age, education, assets, and marital status.
Elizabeth Barrette-Conner, 1999	cohort	community	70.2	4	male	NA	cognitive decline	total T<br>bio-T<br>total-E2<br>bio-E2	adjusted for age, education, alcohol use, BMI, and smoking.
S. D. Chung, 2016	cohort	community	72.2	5	male	NA	AD	ADT	adjusted for patients' age, geographical location, monthly income, and urbanization level.
Kevin T. Nead, 2016	cohort	hospital	70.9: 66.7	2.7 (1.0–5.4)	male	NA	AD	ADT	NA
Jung Eun Yoo, 2020	cohort	community	61.2	5.74	female	ACD: 212,227, AD: 162,901,	ACD	age at menarche (years)<br>age at menopause (years)<br>duration of fertility (years)<br>parity<br>nulliparity	adjusted for age at menarche, age at menopause, parity, duration of breast feeding, duration of HRT, duration of OC use, alcohol consumption, smoking, regular exercise, income, body mass index, hypertension, diabetes mellitus, dyslipidemia, and

								duration of breast feeding (months)	cancer; adjusted for duration of fertility, parity, duration of breast feeding, duration of HRT, duration of OC use, alcohol consumption, smoking, regular exercise, income, body mass index, hypertension, diabetes mellitus, dyslipidemia, and cancer.
Hyesue Jang, 2018	case-control	community	＞70	NA	female	MCI: 896<br>AD: 118	cognitive decline; AD	No. of completed pregnancy with childbirth<br>No. of incomplete pregnancy without childbirth<br>age at menopause<br>length of reproductive period<br>breastfed	adjusted for age; total years of education; socioeconomic status; employment; history of diabetes mellitus, hypertension, and hyperlipidemia; Geriatric Depression Scale Short Form score; age at menopause; and length of reproductive period.
Natalie L. Rasgon, 2005	case-control	community	74.7:71.9	NA	female	NA	cognitive decline	age at menarche, years<br>age at menopause, years<br>reproductive period, years<br>parity	adjusted for age and education.
Li-Ting Kao, 2017	cohort	community	74.2:69.3	5	male	incidence rate 2.12%	dementia	ADT	adjusted for geographical location, monthly income, urbanization level, and age.
Kevin T. Nead, 2016	cohort	hospital	66.9	3.4 (1.0-7.2)	male	314	dementia	ADT	adjusted for age; race/ethnicity; smoking status; anticoagulant, antiplatelet, antihypertensive, and statin therapy; and history of cardiovascular disease, type 1 or 2 diabetes, stroke, or malignant neoplasm.
Myungsun Shim, 2020	cohort	community	72.8: 66.8	4	male	354	dementia	GnRHa	adjusted for age at diagnosis, residence (urban vs. suburban/rural), socioeconomic status (National health insurance vs. Medicaid), prior medication, prior antiandrogen use, past medical history, and

									comorbidity index for the nationwide cohort. For hospital validation cohort, alcohol consumption, smoking status, body mass index, and prostate specific antigen were added for adjustment.
David Robinson, 2019	cohort	hospital	76	4.3 (2.8–6.3)	male	9072	ACD	no prostate cancer<br>watchful waiting.<br>antiandrogen<br>GnRH agonists<br>orchidectomy	adjusted for CCI, educational level and civil status.
Joanne Ryan, 2009	cohort	community	72.8	4	female	NA	cognitive decline	age at first menses<br>age at menopause<br>reproductive years<br>surgical menopause<br>nulliparous<br>number of children<br>age at birth of first child	adjusted for age, educational level and baseline cognitive test score.
Ineke R. Postma, 2016	cohort	hospital	39: 40	6	female	NA	cognitive decline	(pre)eclampsia	NA
Bulent Gunlusoy, 2016	cohort	hospital	67.12:66.84	1	male	NA	cognitive decline	ADT	NA
Sanna L. Read, 2017	cohort	community	65.2	8	female	NA	cognitive decline	number of children	adjusted for age, education, occupational status, tenure status, wealth, limiting long-term illness, physical activity, smoking, depressive symptoms, control, partner, face-to-face contact, activities.
S.D. Moffat,	cohort	community	66.3	19.1(4-37)	male	AD:54,	AD	testosterone and SHBG	adjusted for age, years of education, ever smoke,

2004						dementia:68	dementia		body mass index diabetes, any cancer diagnoses, hormone supplement.
Emily W. Harville, 2019	cohort	community	47.7	NA	female	NA	cognitive decline	nulliparous<br>any fertility difficulties<br>birth <16 years<br>birth<18 years<br>low birthweight<br>preterm birth<br>gestational diabetes<br>hypertensive disorders<br>miscarriage<br>ever breastfed<br>total lifetime breastfed	adjusted for age, menopausal status, race, smoking, education, vocabulary, BMI, last known marital status, depressive symptoms.
Majon Muller, 2010	cohort	community	77.4	5.2 (3.1)	female (70.3%); male (29.7%)	dementia: 146. AD:91	dementia AD	SHBG	adjusted for age (timescale), ethnic group, education, APOE genotype, smoking, BMI, cholesterol, insulin, hypertension, diabetes.
Farzin Khosrow-Khavar, 2016	cohort	hospital	70.7	4.3 (3.6)	male	799	dementia AD	ADT type<br>GnRH agonists<br>oral antiandrogens<br>GnRH agonists and oral antiandrogens<br>other types or combinations<br>duration of ADT use, months	adjusted for alcohol-related disorders; smoking status; body mass index; modified Charlson comorbidity index; prostate-specific antigen; history of head injury; use of antidepressants, antipsychotics, benzodiazepines, and hypnotics; and year of prostate cancer diagnosis.
Gail A. Laughlin,	cohort	community	69	8.3	female	NA	cognitive decline	oestrone	adjusted for age, education and mean CFT score.

2010									
Juan Morote, 2017	cohort	hospital	71.2	0.5	male	NA	cognitive decline	LHRH	NA
Xingyue Song, 2020	cohort	community	53.4	16-23	female	1332	cognitive decline	age at menarche<br>age at first livebirth<br>age at menopause<br>duration of reproductive period	adjusted for age at MMSE measurement, year of baseline interview, dialect group, marital status, and education level, smoking status, tea intake, coffee intake, sleep duration, physical activity, body mass index, total energy intake, alternate Mediterranean dietary pattern score, baseline history of hypertension, diabetes, cardiovascular disease, cancer, age at menarche, number of children, use of oral contraceptives, age at menopause and use of hormone replacement therapy
Alain K. Koyama, 2016	cohort	hospital	60.1	22.6±0.4	female	NA	cognitive decline	estrone<br>estrone sulfate<br>estradiol<br>androstenedione<br>testosterone<br>DHEA	adjusted for age, occupation, BMI, alcohol use, physical activity, age at menopause, depression status, antidepressant use.
Leung-Wing Chu, 2010	cohort	community	72.7	1	male	10	AD	serum testosterone level	adjusted for age, education, BMI, fasting plasma glucose, and serum HDL-C levels.
Molly Fox, 2018	case-control	community	77:86	NA	female	NA	AD	age at menarche<br>age at menopause<br>age at natural menopause<br>reproductive span<br>age at first birth	adjusted for age at first birth, reproductive span, and history of breastfeeding, marriages, and occupation.

								parity, continuous<br>cumulative months pregnant<br>breastfeeding duration<br>hysterectomy<br>bilateral oophorectomy<br>parity<br>parous<br>miscarriages<br>medical abortions	
Supriya Gupta Mohile, 2010	cohort	hospital	71	0.5	male	NA	cognitive decline	at least 6 months of ADT	NA
Ellika G Andolf, 2007	cohort	community	26.1	35	female	608	dementia.	hypertension without proteinuria<br>hypertension and proteinuria<br>preeclampsia<br>preeclampsia + eclampsia	adjusted for mother's age at birth, mother's attained educational level in 1985, marital status and origin (Nordic/non-Nordic) any history of cardiovascular disease later in life (diabetes, atherosclerosis, stroke, ischemic heart disease, heart failure and hypertension).
Jenna Najar, 2020	cohort	community	Q1:50.1<br>Q2:53.0<br>Q3:52.7<br>Q4:62.3	26.8±10.2	female	dementia: 291.<br>AD：146	dementia<br>AD	reproductive period (years)<br>breastfeeding (months)<br>pregnancies (number)<br>age at menarche<br>age at menopause	adjusted for reproductive period, number of pregnancies, months of breastfeeding, birth year, exogenous estrogen, physical activity, WHR, hypertension, ischemic heart disease, and psychological stress.<br>adjusted for age at menarche, age at menopause, number of pregnancies, months of breastfeeding, birth year, psychological stress, and hypertension.

Kristine Yaffe, 1998	cohort	community	71.9	5	female	NA	cognitive decline	estrone<br>estradiol	adjusted for age.
Wen-Kuan Huang, 2020	cohort	community	73	3.46 (1.92-5.51)	male	1525	dementia<br>AD	no ADT<br>GnRH agonist<br>orchiectomy<br>antiandrogen monotherapy	adjusted for urbanization, monthly income, enrollee category, comorbidities, benzodiaepine, and anti-cholinergic use, and number of clinical visits.
Saima Basit, 2018	cohort	community	＜45	21.1 (11.3-23.4)	female	dementia: 1728, AD:676.	dementia<br>AD	pre-eclampsia	adjusted for maternal birth year, parity, region of most recent delivery, cardiovascular disease, stroke, chronic kidney disease, hypertension, and diabetes.
Mette Norgaard, 2021	cohort	community	67.5	6.9 (3.6-11.6) and 6.4 (3.4-10.8)	male	ACD: 28652<br>AD:13549	dementia<br>AD	BPH	adjusted for cardiovascular disease, diabetes mellitus, hyperlipidemia, hypertension, obesity, chronic obstructive pulmonary disease, use of antihypertensive drugs, use of NSAIDs, income in the year before index date, and employment status in the year before index date, and renal failure (time-varying).
Hung-Tse Chou, 2021	case-control	community	63.7	NA	female	75	cognitive decline	age of menarche (year)<br>reproductive period (years)<br>irregular menstrual cycle<br>birth history<br>birth times<br>breast feeding history<br>breastfeeding period (months)	adjusted for age, cerebrovascular disease, education level, living alone, BMI, total cholesterol, LDL-C, menarche age, reproductive period, breath times and breastfeeding period.
J Ryan, 2014	cohort	community	Age at menopause: >50 73.9	7	female	3739	cognitive decline<br>dementia	age at menopause (years)<br>surgical menopause	adjusted for baseline cognitive function, recruitment center, age, education level, physical limitations, chronic illness, depression, use of HT at the

			46–50 74.2<br>41–45 74.6<br>≤40 74.1						menopause and current HT use, also adjusted for APOE-e4.
fu-Dong Li, 2020	case-control	community	69.2	NA	female	1053	cognitive decline	mean breastfeeding duration	adjusted for age, race, education level, marital status, family income, body mass index, physical activity, hypertension, Activities of Daily Living Scale scores, and patient health questionnaire-9 scores, plus reproductive period, age at first birth, parity, incomplete pregnancy, and oral contraceptives use.
Laure Carcaillon, 2014	cohort	community	Noncases: 74.4<br>ACD: 78.7	4	female	dementia ： 132.<br>AD:90.	dementia<br>AD	testosterone<br>estradiol	adjusted for age, center, education, APOE e4, depressive symptoms, waist-to-hip ratio, Mini-Mental State Examination score at baseline, hypercholesterolemia, and history of myocardial infarction and stroke.
Bum Sik Tae, 2018	cohort	hospital	71.2:65.9	4.09±2.33	male	4,743	cognitive decline	no ADT<br>ADT < 12 mo<br>ADT ≥ 12 mo	NA
Bushra Imtiaz, 2014	case-control	community	81	NA	female	NA	AD	oophorectomy<br>hysterectomy<br>hysterectomy with bilateral oophorectomy	adjusted for use and duration of HT and modified Charlson comorbidity index.
K. Yaffe, 2006	cohort	community	75.2	2	female (45.2%)<br>male (54.8%)	NA	cognitive decline	testosterone<br>estradiol	adjusted for age, race, education, study site, baseline cognitive function, hypertension, and weight,
Thien Kieu	cohort	community	＞40	29	female	95,239	dementia	hysterectomy	adjusted for hysterectomy, unilateral oophorectomy,

Thi Phung, 2010								unilateral oophorectomy<br>bilateral oophorectomy<br>no hysterectomy	bilateral oophorectomy, and indication for operation.
Julie A. Fields, 2017	cohort	hospital	59.2:59.6	34.9 (32.0-47.2); 34.5 (32.0-46.4)	female	13	dementia or cognitive decline	pre-eclampsia	NA
Keiko Kurita, 2016	cohort	community	60.7	2.7	female	NA	cognitive decline	oophorectomy	adjusted for trial, age, education, race/ethnicity, body mass index, treatment indicator, and the baseline cognitive domain score.
Sri Suravarapu, 2006	cohort	community	72.7	10.25 (9.07-10.68)	male	21	dementia	testosterone	adjusted for age and BMI.
Ravishankar Jayadevappa, 2018	cohort	hospital	76.0:74.3	8.3 (4.7)	male	ADT:16755; dementia: 27974	dementia; AD	1-4 ADT doses<br>5-8 ADT doses<br>>8 ADT doses<br>No ADT	adjusted for age at diagnosis, race/ethnicity, geographic area, marital status, comorbidity score, cancer stage, and socioeconomic status.
Paola Gilsanz, 2019	cohort	community	51.1	9.1 (6.3) (0.003-21.7)	female	2,577	dementia	menarche, y<br>menopause, y<br>reproductive span, y<br>hysterectomy	adjusted for dementia associated with age at menarche, age at menopause, duration of reproductive span, and hysterectomies.
Onder Cinar, 2021	cohort	hospital	69.08	0.5	male	NA	cognitive decline	ADT	NA
Joanne Ryan, 2012	cohort	community	60	2	female	NA	cognitive decline	estrone<br>testosterone<br>estradiol	adjusted for age, education, depressive symptoms, age at menopause and baseline cognitive score.

Seo Hyon Baik, 2017	cohort	hospital	75.21	5.5	male	AD:109,815<br>dementia: 223,765	AD<br>dementia	ADT	adjusted for other cancer therapies and other covariates.
Mirjam I. Geerlings, 2001	cohort	community	Reproductive Period, y<br>＜34 71.7<br>34-36 70.3<br>37-39 70.0<br>＞39 68.7<br>Artificial 68.3	6.3<br>(0.0-9.4)	female	dementia: 199<br>AD:159	dementia<br>AD	reproductive period, y<br>artificial menopause<br>per y increase in reproductive period<br>age at menarche, y<br>age at menopause, y	adjusted for age, education, smoking status, alcohol intake, body mass index, hormone replacement therapy, number of children, and apolipoprotein E genotype.
Jong Bin Bae,2020	cohort	community	73.1	NA	female	NA	dementia	0 (nulliparity)<br>≥ 5 (grand multiparity)<br>5 to 9<br>≥ 10	adjusted for age, educational level, hypertension, diabetes mellitus, and cohort as covariates.
Michelle M. Mielke, 2016	cohort	community	＞57	NA	female	NA	cognitive decline	normotensive pregnancy<br>hypertensive pregnancy	adjusted for age, race, education, body mass index, smoking, hypertension, family history of hypertension, with hypertension duration, which was assessed for women with current hypertension.
L.-F. Low, 2005	case-control	community	62.5	NA	female	NA	cognitive decline	reproductive period	adjusted for age, education and verbal intelligence, health and mood variables, lifestyle variables, and other variables that impact on lifetime estrogen exposure.
Michelle Heys, 2010	cohort	community	60.4	NA	female	NA	cognitive decline	reproductive period (years)<br>age of first pregnancy (years)<br>parity	adjusted for age (continuous), education, parental possessions, occupation, and physical activity.

								duration of breast feeding per child (years)	
Erin S. LeBlanc, 2010	cohort	community	73	4.5	male	NA	cognitive decline	testosterone<br>estradiol<br>SHBG	adjusted for age group, education level (complete high school or less vs. complete college or more), race, self-rated health, and alcohol consumption.
Yasin Ceylan, 2019	cohort	hospital	67.27:66.65	1	male	NA	cognitive decline	ADT	NA
Mirjam I. Geerlings, 2006	cohort	community	77.4	6.1 (4.4–7.8)	male	dementia: 233<br>AD:134	dementia<br>AD	testosterone<br>estradiol	adjusted for age (timescale), years of education, Cognitive Abilities Screening Instrument (CASI) score at baseline, smoking habits, alcohol intake, body mass index, physical activity, metabolic syndrome, diabetes, and depression.
Laure Carcaillon, 2013	cohort	community	74.5	3.1±1.0	male	dementia: 105<br>AD:62	dementia<br>AD	testosterone<br>estradiol	adjusted for age, center, educational level, body mass index, apolipoprotein E ε4 level, Mini-Mental State Examination score at baseline, diabetes mellitus, hypertension, hypercholesterolemia, tobacco status, history of coronary heart disease, and stroke.
Shabbir M.H. Alibhai, 2016	cohort	hospital	68.9	3	male	NA	cognitive decline	ADT	adjusted for age and education.
Jihun Kang, 2020	cohort	community	68.5:67.8	5.2	male	dementia: 26,590<br>AD:19,763	dementia<br>AD	ADT	adjusted for age, income, Charlson comorbidity index, diabetes mellitus, hypertension, dyslipidemia, aspirin use, statin use, smoking status, BMI, blood glucose, systolic blood pressure, and total cholesterol.
Saima Basit, 2019	cohort	community	<42	21.6 (11.6–23.4)	female	dementia: 2188	dementia<br>AD	stillbirth<br>miscarriages	adjusted for birth year (5-year intervals), parity (1, 2, !3), age, cardiovascular disease, hypertension, and

						AD:860			diabetes. (dementia)<br>adjusted for birth year (5-year intervals), parity (1, 2, ≥3), and age (the underlying time scale in the Cox model) (AD and VaD)
Riley Bove, 2014	cohort	community	78	18	female	592	AD	surgical menopause	adjusted for age at enrollment, education (years), smoking (pack-years), and study (ROS vs MAP).
A. Paganini-Hill, 2020	cohort	community	93.2	3.4	female	209	dementia	age at menarche<br>number of children<br>age at first child<br>no child<br>surgical menopause without bilateral oophorectomy<br>bilateral oophorectomy<br>age at last menstrual period<br>reproductive years	adjusted for education.
Sujarwoto, 2019	case-control	community	68.14	NA	female	NA	cognitive decline	premature menopause<br>early onset menarche<br>number of biological children<br>number of stillbirths<br>number of miscarriages	adjusted to account for other confounding factors (i.e. age, marital status, education, household expenditure, number of cigarettes consumed per day, grams of tobacco consumed per day, obesity, mental depression, and living in urban areas).
Robert N. McLay, 2003	cohort	community	63.4	12.8	female	NA	cognitive decline	menopause<br>childbirth	adjusted for age, race, education, MMSE score at wave 1, childbirth, and age at menopause.
Carolien N.H. Abheiden, 2015	case-control	hospital	67.1:64.2	NA	female	NA	AD	hypertensive disorders of pregnancy<br>pregnancy-induced hypertension<br>preeclampsia	adjusted for age and BMI.

								eclampsia	
Jong Won Kim, 2021	cohort	community	72.72:64.11	ADT (3.24±2.07)<br>Non-ADT (3.50±2.28)	male	NA	dementia<br>AD	ADT use<br>duration of ADT (y)	adjusted for age.
Andrew H, 2018	cohort	community	＞75	10.5 (9.4-12.2)	male	499	dementia	testosterone<br>estradiol<br>dihydrotestosterone<br>LH<br>SHBG	adjusted for age, baseline cognitive function, depression, body mass index, hypertension, cardiovascular disease, and total plasma homocysteine.
Chiara Zucchella, 2012	case-control	hospital	77.6: 76.7	NA	female	NA	AD	age at puberty<br>number of pregnancies<br>abortion<br>contraceptive therapy<br>age at menopause<br>reproductive life span	NA
Giovanni Ravaglia, 2007	cohort	community	women:74<br>men:73	women: 3.9±0.7<br>men: 3.8±0.8	Female (53.5%); male (46.5%)	dementia: 110<br>AD:69	dementia<br>AD	LH	adjusted for age, age at menopause, education, apolipoprotein E 4 genotype, smoking status, and body mass index, stroke, cardiovascular disease, diabetes, hyperhomocysteinemia, serum folate, serum vitamin B12, and serum creatinine.
Fu-Dong Li, 2015	case-control	community	69.8	NA	female	919	cognitive decline	reproductive period<br>regularity of menstrual cycle<br>length of menstrual cycle<br>number of full-term pregnancies<br>number of incomplete pregnancies	adjusted for potential confounders (age, race, education, marital status, economic status, smoking, alcohol drinking, exercise, hypertension, and depressive symptom).

Jessica Gong, 2022	cohort	community	56.3	11.8	female	273,240	dementia	age at menarche<br>ever been pregnant<br>number of live births<br>parous versus not<br>age at first live birth<br>number of miscarriages<br>number of stillbirths<br>number of abortions<br>reproductive period<br>age at natural menopause<br>hysterectomy versus not<br>oophorectomy versus not	adjusted for age, Townsend index, ethnicity, smoking status, systolic blood pressure, BMI, diabetes, total cholesterol, antihypertensive drugs, and lipid lowering drugs.
Changyong Yu, 2022	case-control	community	87.16	NA	female	2,039	cognitive decline	parity	NA
Margot J Overman, 2022	cohort	community	60	4	male	2,736	dementia	testosterone<br>SHBG	adjusted for age, age left education, BMI, BDI score, smoking (non- vs current), alcohol consumption (<1 vs ≥ 1 day/week), psychotropic drug use (none vs any), center, PASE score, PPT walking speed, and co-morbidities (0, 1, or 2 comorbidities).
Alison Gemmill, 2022	cohort	community	62.08	13	female	8,875	dementia	age at first birth<br>parity	adjusted for race/ethnicity, birthplace, education, parental education, partnership status, smoking status, BMI, depressive symptomology, and medical comorbidities.
N. Araújo, 2022	cohort	hospital	68	1	male	366	cognitive decline	ADT	adjusted for age and education.

Joseph L. Saenz, 2021	cohort	community	>60	12	female	6,541	cognitive decline	parity	adjusted for fertility history and confounding variables (age, rural/urban, marital status, age at first marriage, parental education, own education, and chronic conditions), and employment, income, and wealth to determine whether fertility history-cognition associations were explained by economic mechanisms, and depressive symptoms and loneliness to determine whether fertility history-cognition associations were driven by psychosocial variables.
Jui-Ming Liu, 2021	cohort	community	70	>4	male	87,655	dementia	ADT	adjusted for age
Onder Cinar, 2021	cohort	hospital	69.08	0.5	male	48	cognitive decline	ADT	NA
Lina Bergman, 2021	cohort	hospital	25	0.5	female	153	cognitive decline	pre-eclampsia	NA
Hector Alonso-Quiñones, 2021	cohort	community	78	3.8	male	241	cognitive decline	ADT	adjust for education, APOE genotype, depression, and the Charlson comorbidity index.
Karl H. Tully, 2019	cohort	hospital	52	9.3	male	9,117	dementia	ADT	NA
Peter E. Lonergan, 2022	cohort	hospital	>50	7	male	13,570	dementia	ADT	NA

C. S. Uldbjerg, 2022	cohort	hospital	>60	13	female	24,851	dementia	oophorectomy		NA

Abbreviations: ACD: all cause dementia, AD: Alzheimer's disease, ADT: androgen deprivation therapy, APOE: apolipoprotein E, BMI: body mass index, BPH: benign prostate hyperplasia, CCI: Charlson comorbidity index, CFT: Complex Figure Test, DHEA: dehydroepiandrosterone, GnRHa: gonadotropin-releasing hormone agonist, HDL-C: high-density lipoprotein cholesterol, HRT: hormone replacement therapy, HT: hormone therapy, LDL-C: low-density lipoprotein cholesterol, LH: luteinizing hormone, LH-RH: luteinizing hormone releasing hormone, MCI: mild cognitive impairment, MMSE: mini-mental state examination, NSAIDs: non-steroidal anti-inflammatory drugs, OC: oral contraception, SHBG: sex hormone binding, SLE: systemic lupus erythematosus, VaD: vascular dementia, WHR waist-to-hip ratio.

Appendix F Measurement of the outcomes on included studies.

First author, year	Outcome	Outcome measurement
Mirjam I. Geerlings, 2002	dementia<br>AD	Dementia: DSM-III-R,<br>AD: NINCDS-ADRDA,<br>three-step diagnostic procedure
Majon Muller, 2008	cognitive decline	decline in MMSE-score of 4 points or more after 4 years of follow-up
Ellika Andolf, 2020	ACD	ICD-10
Chun-Ming Yang, 2015	AD	ICD 9-CM
Vanessa Sánchez-Martínez, 2021	cognitive decline	MMSE score<br>the Brief Scale for Cognitive Evaluation (BCog) score
M Nelander, 2017	dementia	ICD codes
Yoko Shimizu, 2019	cognitive decline	the Mini Mental State Examination (MMSE), the Wechsler Memory Scale Revised (WMS-R) Logical Memory I/II subtest, the clock drawing test, and the Clinical Dementia Rating (CDR) Scale
Kristine Yaffe, 2010	cognitive decline	a decline of 3 or more points on the mMMSE from baseline to the 6-year examination.
W.A. Rocca, 2007	dementia or cognitive decline	1) they scored ＜27 on the TICS-m at direct interview;<br>2) a proxy respondent reported a previous diagnosis of dementia, senility, or AD;<br>3) a proxy respondent reported impairment in activities of daily living (dressing, eating, or bathing) caused by cognitive problems.
C.N. Lessov-Schlaggar, 2005	cognitive decline	MMSE
Kohei Okamoto, 2015	cognitive decline	MMSE
Martin J. Prince, 2018	dementia	two cognitive tests;<br>the 32-item Community Screening Instrument for Dementia (CSI-D) COGSCORE and the modified Consortium to Establish a Registry for Alzheimer's Disease (CERAD) 10-word list learning task with delayed recall
Elizabeth Barrette-Conner, 1999	cognitive decline	MMSE
S. D. Chung, 2016	AD	ICD-9-CM code
Kevin T. Nead, 2016	AD	ICD-9 code
Jung Eun Yoo, 2020	ACD	ICD-10 codes
Hyesue Jang, 2018	cognitive decline;<br>AD	AD: NINCDS-ADRDA;<br>Cognitive decline: the consensus criteria from the International Working Group on Mild

		Cognitive Impairment.
Natalie L. Rasgon, 2005	cognitive decline	TELE, a telephone-based interview score ≤13.5 points (max 19)
Li-Ting Kao, 2017	dementia	ICD-9-CM codes
Kevin T. Nead, 2016	dementia	ICD-9 codes
Myungsun Shim, 2020	dementia	ICD-10 codes
David Robinson, 2019	ACD	ICD-10 codes
Joanne Ryan, 2009	cognitive decline	Global function, MMSE < -2
Ineke R. Postma, 2016	cognitive decline	CDR scale
Bulent Gunlusoy, 2016	cognitive decline	MoCA；FAB tests
Sanna L. Read, 2017	cognitive decline	16 neurocognitive tests
S.D. Moffat, 2004	AD<br>dementia	DSM-III-R,<br>NINCDS-ADRDA
Emily W. Harville, 2019	cognitive decline	Cognitive domains tests
Majon Muller, 2010	dementia<br>AD	Dementia: DSM-IV criteria and required evidence of cognitive deficit on the neuropsychological test battery as well as evidence of impairment in social or occupational function (Clinical Dementia Rating of1 or more).<br>AD: NINCDS-ADRDA criteria
Farzin Khosrow-Khavar, 2016	dementia<br>AD	Read codes
Gail A. Laughlin, 2010	cognitive decline	MMSE<br>Trails B<br>Category fluency
Juan Morote, 2017	cognitive decline	four domains:1) working memory,2) visual memory,3)visuospatial ability,4)nonverbal analytical reasoning
Xingyue Song, 2020	cognitive decline	Singapore Modified Mini-Mental State Examination (SMMMSE)<br>The cut-off points were 17/18 for subjects with no formal education, 20/21 for subjects with primary school education and 24/25 for those with secondary school or higher education.
Alain K. Koyama, 2016	cognitive decline	the Telephone Interview of Cognitive Status (TICS).
Leung-Wing Chu, 2010	AD	NINCDS-ADRDA criteria; the Chinese versions of Mini-Mental State Examination (MMSE), AD Assessment Scale-cognitive subscale (ADAS-cog), and Delayed 10-Word Recall Test (DWRT)
Molly Fox, 2018	AD	CDR scale
Supriya Gupta Mohile, 2010	cognitive decline	a standardized neuropsychological battery
Ellika G Andolf, 2007	dementia.	ICD 9

Jenna Najar, 2020	dementia<br>AD	DSM-III-R criteria<br>NINCDS-ADRDA
Kristine Yaffe, 1998	cognitive decline	MMSE, -12.5 points or more
Wen-Kuan Huang, 2020	dementia<br>AD	ICD-9-CM and ICD-10-CMcodes
Saima Basit, 2018	dementia<br>AD	ICD-10
Mette Norgaard, 2021	dementia<br>AD	ICD-10
Hung-Tse Chou, 2021	cognitive decline	A MMSE cut-off score of 24
J Ryan, 2014	cognitive decline dementia	MMSE decline ＞3<br>a three-step procedure,<br>double blind diagnosis based on ICD-IV
fu-Dong Li, 2020	cognitive decline	MMSE
Laure Carcaillon, 2014	dementia<br>AD	Dementia: A 2-step procedure: a neuropsychological examination; DSM-IV-R criteria for dementia,<br>AD: NINCDS-ADRDA
Bum Sik Tae, 2018	cognitive decline	ICD-10 codes
Bushra Imtiaz, 2014	AD	NINCDS-ADRDA and the DSM-IV
K. Yaffe, 2006	cognitive decline	3MS decline scoring ≥5 fewer points
Thien Kieu Thi Phung, 2010	dementia	ICD codes
Julie A. Fields, 2017	dementia or cognitive decline	Cognitive decline: mild impairment on two or more measures within a single domain, or mild impairment on one or more measure within at least two domains. dementia: cognitive deficits ≥2SD below the main in two or more domains.
Keiko Kurita, 2016	cognitive decline	The cognitive battery
Sri Suravarapu, 2006	dementia	Diagnostic and Statistical Manual of Mental Disorders, 4th ed
Ravishankar Jayadevappa, 2018	dementia;<br>AD	(International Classification of Diseases, Ninth Revision, Clinical Modification [ICD-9-CM] codes 290, 29420, 29411, 29282, 2912, and 29421) or Alzheimer disease (ICD-9-CM code 3310)
Paola Gilsanz, 2019	dementia	Electronic medical records using the following ICD10 diagnosis codes
Onder Cinar, 2021	cognitive decline	Neuropsychological tests
Joanne Ryan, 2012	cognitive decline	The neuropsychological test battery
Seo Hyon Baik, 2017	AD<br>dementia	CMS coding
Mirjam I. Geerlings, 2001	dementia<br>AD	dementia: DSM- III,<br>AD: NINCDS-ADRDA

Jong Bin Bae,2020	dementia	DSM-IV criteria
Michelle M. Mielke, 2016	cognitive decline	MMSE
L.-F. Low, 2005	cognitive decline	MMSE
Michelle Heys, 2010	cognitive decline	CERAD (Consortium to Establish a Registry for Alzheimer's Disease) test battery
Erin S. LeBlanc, 2010	cognitive decline	3MS (-10 as the cut point)
Yasin Ceylan, 2019	cognitive decline	A verbal learning test (immediate recall and 1-minute delay), the Mini-Mental State Examination (MMSE), digit span backwards, the Symbol-Digit Modalities Test and simple and choice reaction time tests
Mirjam I. Geerlings, 2006	dementia<br>AD	Dementia: DSM- III, DSM-IV criteria,<br>AD: NINCDS-ADRDA
Laure Carcaillon, 2013	dementia<br>AD	Dementia: DSM-IV criteria, CDR scale,<br>AD: NINCDS-ADRDA
Shabbir M.H. Alibhai, 2016	cognitive decline	14 neuropsychological tests
Jihun Kang, 2020	dementia<br>AD	ICD codes
Saima Basit, 2019	dementia<br>AD	ICD-10
Riley Bove, 2014	AD	AD: NINCDS-ADRDA criteria (a 2-step process)
A. Paganini-Hill, 2020	dementia	(1) neurological examination, (2) MMSE, (3) informant questionnaires, and (4) CASI-short<br>DSM-IV criteria
Sujarwoto Sujarwoto, 2019	cognitive decline	Telephone Survey of Cognitive Status (TICS)
Robert N. McLay, 2003	cognitive decline	MMSE
Carolien N.H. Abheiden, 2015	AD	Medical records
Jong Won Kim, 2021	dementia<br>AD	ICD-10 codes
Andrew H, 2018	dementia	Medical records
Chiara Zucchella, 2012	AD	the NINCDS-ADRDA criteria
Giovanni Ravaglia, 2007	dementia<br>AD	DSM-IV;<br>NINCDS-ADRDA;
Fu-Dong Li, 2015	cognitive decline	cut-off point to define cognitive impairment in China (MMSE Chinese Standard, MCS): 17/18 for illiteracy, 20/21 for people with primary education level, 24/25 for people with higher than primary education level.
Jessica Gong, 2022	dementia	ICD-10 codes
Changyong Yu, 2022	cognitive decline	MMSE
Margot J Overman, 2022	dementia	the Rey-Osterrieth Complex Figure Copy and

		Recall, the Camden Topographical Recognition Memory and the Digit Symbol Substitution Test.
Alison Gemmill, 2022	dementia	cognitive scale
N. Araújo, 2022	cognitive decline	MOCA
Joseph L. Saenz, 2021	cognitive decline	cognitive tasks
Jui-Ming Liu, 2021	dementia	ICD codes
Onder Cinar, 2021	cognitive decline	four neuropsychological tests, including the Symbol Digit Modalities Test (SDMT), the California Verbal Learning Test, second edition (CVLT-II); the Brief Visuospatial Memory Test—Revised (BVMT-R); and the Trail Making Test (TMT).
Lina Bergman, 2021	cognitive decline	MOCA
Hector Alonso-Quiñones, 2021	cognitive decline	the neuropsychological battery
Karl H. Tully, 2019	dementia	ICD-9 codes
Peter E. Lonergan, 2022	dementia	ICD codes
C. S. Uldbjerg, 2022	dementia	ICD codes

Abbreviations: CDR, the Clinical Dementia Rating scale; DSM: Diagnostic and Statistical Manual of Mental Disorders; ICD, International Classification of Diseases; MMSE, Mini-Mental State Examination; MoCA, Montreal cognitive assessment; NINCDS-ADRDA, National Institute of Neurological and Communicative Disorders and Stroke and the Alzheimer's Disease and Related Disorders Association criteria.

Appendix G Characteristics of studies with cognitive decline, dementia, and AD.

1. Characteristics of studies with cognitive decline, dementia, and AD by menstrual and parity.

   a) Characteristics of studies with cognitive decline, dementia, and AD by age at menarche.

**Author**	**Project**	**Year**	**Description of risk factor**	**RR/OR/HR**	**Adjusted risk**	**LCI**	**UCI**	**Outcome**	**design**	**Country**	**Sample**	**Case**	**Score**
Yoko Shimizu	JPHC Study	2019	Age at menarche, years					cognitive decline	cohort	Japan	670	196	7.5
			≤ 13 (ref.)	OR	1.00	1.00	1.00				238	63	
			14-15	OR	1.28	0.87	1.89				320	113	
			≥ 16	OR	1.70	0.96	2.99				92	46	
Hyesue Jang	KLOSCAD+ HELIAD	2018	Age at menopause	OR	0.99	0.95	1.04	cognitive decline	case-control	Korea	3,549	896	5.5
Natalie L. Rasgon	HARMONY	2005	Age at menarche, years					cognitive decline	case-control	US	1111/4733	1,111	6
			＜12	OR	1.21	0.85	173.00				234	42	
			12–14 (ref.)	OR	1.00	1.00	1.00				3,426	592	
			＞14	OR	1.19	1.03	1.38				1,816	372	
Joanne Ryan	ESPRIT	2009	Age at first Menses	OR	1.05	0.95	1.16	cognitive decline	cohort	France	996	NA	4.5
Xingyue Song	SCHS	2020	Age at menarche, years					cognitive decline	cohort	Singapore	8,222	1,332	7.5
			<13 (ref.)	OR	1.00	1.00	1.00				2,034	260	
			13–14	OR	1.02	0.86	1.22				3,046	459	
			15–16	OR	1.13	0.94	1.36				2,325	430	
			>16	OR	1.18	0.93	1.49				817	183	
			Per 1-year increase	OR	1.04	1.00	1.07						
Martin J. Prince	10/66 population-based study	2018	Age at menarche, years	HR	0.986	0.944	1.030	dementia	cohort	Cuba, Dominican Republic,	8466	692	5.5

										Puerto Rico Venezuela, Mexico, Peru, China			
Jung Eun Yoo	NCSP	2020	Age at menarche, years					dementia	cohort	Korea	4,696,633	212,227	7
			≤ 12	HR	1.07	1.01	1.14				63,275	1134	
			13-14 (ref.)	HR	1.00	1.00	1.00				680953	15339	
			15-16	HR	1.07	1.05	1.09				1879203	70707	
			≥ 17	HR	1.15	1.13	1.16				2,073,202	125047	
Paola Gilsanz	KPNC	2019	Menarche, years					dementia	cohort	US	6,137	2577	7
			<13.0	HR	0.97	0.88	1.07				1,516		
			≥13.0 (ref.)	HR	1.00	1.00	1.00				2,531		
			≤10	HR	0.89	0.63	1.26				95		
			11–12	HR	1.00	0.89	1.13				1,421		
			13 (average) (ref.)	HR	1.00	1.00	1.00				1,188		
			14–15	HR	1.03	0.91	1.17				1,085		
			16–17	HR	1.23	1.01	1.5				258		
Mirjam I. Geerlings	The Rotterdam Study	2001	Age at menarche, years					dementia	cohort	Netherlands	3588	199	5
			＞14 (ref.)	RR	1.00	1.00	1.00					61	
			14	RR	0.89	0.57	1.29					37	
			13	RR	1.00	0.67	1.5					40	
			＜12	RR	1.18	0.82	1.7					61	
Jenna Najar	PPSW	2020	Age at menarche	HR	0.99	0.91	1.09	dementia	cohort	Sweden	1364	291	7

A. Paganini-Hill	The 90+ Study	2020	Age at menarche, years					dementia	cohort	US	424	209	5.5
			≤12 (ref.)	HR	1.00	1.00	1.00				145	67	
			13	HR	1.22	0.87	1.72				130	68	
			14+	HR	0.85	0.61	1.19				149	74	
Jessica Gong	UK Biobank	2022	Age at menarche, years					dementia	cohort	UK	273,240		6.5
			<12	HR	1.20	1.08	1.34					385	
			12	HR	1.07	0.95	1.20					331	
			13 (ref.)	HR	1.00	1.00	1.00					372	
			14	HR	0.97	0.87	1.09					334	
			>14	HR	1.19	1.07	1.34					341	
Jung Eun Yoo	NCSP	2020	Age at menarche, years					AD	cohort	Korea	4,696,633	162,901	7
			≤ 12	HR	1.06	0.99	1.14				63,275	848	
			13-14 (ref.)	HR	1.00	1.00	1.00				680,953	11,663	
			15-16	HR	1.06	1.04	1.09				1,879,203	54,154	
			≥ 17	HR	1.14	1.12	1.16				2,073,202	96,236	
Mirjam I. Geerlings	The Rotterdam Study	2001	Age at menarche, years					AD	cohort	Netherlands	3588	155	6.5
			＞14 (ref.)	RR	1.00	1.00	1.00					52	
			14	RR	0.76	0.48	1.21					28	
			13	RR	0.89	0.56	1.4					30	
			＜12	RR	1.15	0.77	1.72					49	
Jenna Najar	PPSW	2020	Age at menarche	HR	1.01	0.89	1.15	AD	cohort	Sweden	1364	146	7

b) Characteristics of studies with cognitive decline, dementia, and AD by length of menstrual cycle.

Author	Project	Year	Description of risk factor	RR/OR/HR	Adjusted risk	LCI	UCI	Outcome	design	Country	Sample	Case	Score
Yoko Shimizu	JPHC Study	2019	Menstrual cycle length					cognitive decline	cohort	Japan	670	196	7.5
			≤ 26 (ref.)	OR	1.00	1.00	1.00				107	29	
			27-29	OR	1.16	0.69	1.95				264	90	
			≥ 30	OR	1.24	0.64	2.39				81	29	
Fu-Dong Li	ZPHS	2015	Length of menstrual cycle	OR	1.01	0.98	1.04	cognitive decline	case-control	CN	4,796	919	7

c) Characteristics of studies with cognitive decline, dementia, and AD by menstrual regularity.

Author	Project	Year	Description of risk factor	RR/OR/ HR	Adjusted risk	LCI	UCI	Outcome	design	Country	Sample	Case	Score
Yoko Shimizu	JPHC Study	2019	Menstrual regularity					cognitive decline	cohort	Japan	619	196	7.5
			Regular (ref.)	OR	1.00	1.00	1.00				494	161	
			Irregular	OR	1.13	0.74	1.73				125	44	
Fu-Dong Li	ZPHS	2015	Regularity of menstrual cycle (regular)	OR	0.89	0.64	1.23	cognitive decline	case-control	CN	4,796	919	7

d) Characteristics of studies with cognitive decline, dementia, and AD by age at menopause.

Author	Project	Year	Description of risk factor	RR/OR /HR	Adjusted risk	LCI	UCI	Outcome	design	Country	Sample	Case	Score
Yoko Shimizu	JPHC Study	2019	Age at menopause, years					cognitive decline	cohort	Japan	670	196	7.5
			≤ 44 (ref.)	OR	1.00	1.00	1.00				58	23	
			45-49	OR	0.97	0.52	1.79				192	76	
			≥ 50	OR	0.75	0.42	1.35				341	117	
Xingyue Song	SCHS	2020	Age at menopause, years					cognitive decline	cohort	Singapore	8222	1332	7.5
			<45	OR	1.67	1.32	2.11				508	131	
			45–49	OR	1.24	1.08	1.44				2291	419	
			50–54 (ref.)	OR	1.00	1.00	1.00				4361	600	
			54	OR	1.06	0.87	1.29				1062	182	
			Per 1-year increase	OR	0.97	0.96	0.99						
Joanne Ryan	ESPRIT	2009	Age at menopause	OR	0.99	0.96	1.02	cognitive decline	cohort	France	996	NA	4.5
J Ryan	TCS	2014	Age at menopause, years					cognitive decline	cohort	France	4868	NA	7
			After 50 (ref.)	HR	1.00	1.00	1.00				2005		
			46–50	HR	0.96	0.82	1.13				1871		
			41–45	HR	0.83	0.66	1.04				621		
			40 or before	HR	1.35	1.05	1.74				371		
Natalie L. Rasgon	HARMONY	2005	Age at menopause, years					cognitive decline	case-control	US	5844	1111	6
			＜40	OR	1.64	1.03	2.61				102	27	
			40–44	OR	1.39	1.08	1.78				438	102	
			45–49	OR	1.01	0.85	1.20				1414	259	
			50–54 (ref.)	OR	1.00	1.00	1.00				2602	471	

			＞54	OR	0.96	0.77	1.19				823	134	
Hyesue Jang	KLOSCAD+ HELIAD	2018	Age at menopause	OR	0.99	0.95	1.04	cognitive decline	case-control	Korea	3549	896	5.5
W.A. Rocca	MCOSOA	2007	Age at bilateral oophorectomy, years					cognitive decline	cohort	US	2961		7
			T1 (＜43)	HR	1.74	0.97	3.14					13	
			T2 (43–48)	HR	1.68	1.06	2.66					23	
			T3 (＞48)	HR	1.09	0.74	1.61					38	
			T1 +T2 (≤48)	HR	1.70	1.15	2.51					36	
Martin J. Prince	10/66 population-based cohort study	2018	Age at menopause, years	HR	1.00	0.99	1.01	dementia	cohort	Cuba, Dominican Republic, Puerto Rico, Venezuel, Mexico, Peru, China	8466	692	5.5
Jung Eun Yoo	NCSP	2020	Age at menopause, years					dementia	cohort	Korea	4,696,633	212,227	7
			< 40 (ref.)	HR	1.00	1.00	1.00				76635	6308	
			40-44	HR	0.96	0.93	0.98				248056	18440	
			45-49	HR	0.89	0.86	0.91				1218122	59452	
			50-54	HR	0.85	0.83	0.87				2601970	106193	
			≥ 55	HR	0.79	0.77	0.81				551850	21834	
Paola Gilsanz	KPNC	2019	Menopause, years (surgical)					dementia	cohort	US	6,137	2577	7
			<47.4 (mean)	HR	1.19	1.07	1.31				1,645		
			≥47.4 (ref.)	HR	1.00	1.00	1.00				2,402		
			≤30	HR	1.11	0.59	2.09				25		

			31–40	HR	1.20	0.98	1.45				342		
			41–45	HR	1.29	1.12	1.49				745		
			46–50	HR	0.99	0.88	1.11				1,891		
			51–55 (ref.)	HR	1.00	1.00	1.00				1,044		
Paola Gilsanz	KPNC	2019	Menopause, years (natural)					dementia	cohort	US	6,137	2577	7
			<48.5 (mean)	HR	1.15	1.03	1.28				1,239		
			≥48.5 (ref.)	HR	1.00	1.00	1.00				1,952		
			<47.4	HR	1.27	1.14	1.43				926		
			≥47.4	HR	1.00	1.00	1.00				2,265		
			≤30	HR	1.55	0.73	3.30				13		
			31–40	HR	1.26	0.99	1.60				165		
			41–45	HR	1.42	1.21	1.67				436		
			46–50	HR	1.01	0.90	1.14				1,533		
			51–55	HR	1.00	1.00	1.00				1,044		
Mirjam I. Geerlings	The Rotterdam Study	2001	Age at menopause, years					dementia	cohort	Netherlands	3588	199	6.5
			＜48 (ref.)	RR	1.00	1.00	1.00					32	
			48-49	RR	1.24	0.72	2.15					23	
			50-52	RR	1.95	1.28	2.96					75	
			＞52	RR	1.78	1.11	2.88					37	
Jenna Najar	PPSW	2020	Age at menopause	HR	1.07	1.04	1.10	dementia	cohort	Sweden	1364	291	7
J Ryan	TCS	2014	Age at menopause, years					dementia	cohort	France	4868	393	7
			After 50 (ref.)	HR	1.00	1.00	1.00				2005		
			46–50	HR	1.23	0.92	1.64				1871		
			41–45	HR	1.13	0.77	1.67				621		
			40 or before	HR	1.23	0.76	2.00				371		

A. Paganini-Hill	The 90+ Study	2020	Age at last menstrual period, years					dementia	cohort	US	424	209	5.5
			44 (ref.)	HR	1.00	1.00	1.00				99	43	
			45–54	HR	1.19	0.84	1.68				262	137	
			55+	HR	1.13	0.70	1.82				59	28	
Jessica Gong	UK Biobank	2022	Age at natural menopause, years					dementia	cohort	UK	273,240		6.5
			<47	HR	1.32	1.15	1.51					242	
			47 to 49	HR	1.07	0.91	1.26					161	
			50 (ref)	HR	1.00	0.86	1.17					180	
			51 to 52	HR	0.8	0.68	0.94					173	
			53 to 54	HR	0.76	0.62	0.93					107	
			>54	HR	0.93	0.8	1.08					193	
Jung Eun Yoo	NCSP	2020	Age at menopause, years					AD	cohort	Korea	4,696,633	162,901	7
			< 40 (ref.)	HR	1								
			40-44	HR	0.96	0.93	0.99						
			45-49	HR	0.88	0.86	0.91						
			50-54	HR	0.85	0.82	0.87						
			≥ 55	HR	0.79	0.77	0.82						
Mirjam I. Geerlings	The Rotterdam Study	2001	Age at menopause, years					AD	cohort	Netherlands	3588	155	6.5
			<48 (ref.)	RR	1.00	1.00	1.00					30	
			48-49	RR	0.90	0.48	1.68					16	
			50-52	RR	1.64	1.05	2.56					60	
			>52	RR	1.47	0.88	2.46					29	
Jenna Najar	PPSW	2020	Age at menopause	HR	1.07	1.02	1.12	AD	cohort	Sweden	1364	146	7
Riley Bove	POS, MAP	2014	Age at surgical menopause	HR	0.99	0.98	1.00	AD	cohort	US	1,884	592	5.5

Hyesue Jang	KLOSCAD+ HELIAD	2018	Age at menopause	OR	1.05	0.92	1.19	AD	case-control	Korea	3549	118	5.5

e) Characteristics of studies with cognitive decline, dementia, and AD by reproductive period.

Author	Project	Year	Description of risk factor	RR/OR/HR	Adjusted risk	LCI	UCI	Outcome	design	Country	Sample	Case	Score
Yoko Shimizu	JPHC Study	2019	Reproductive period, years					cognitive decline	cohort	Japan	670	196	7.5
			≤ 33 (ref.)	OR	1.00	1.00	1.00				171	73	
			34-37	OR	0.89	0.58	1.38				191	75	
			≥ 38	OR	0.62	0.40	0.96				210	63	
Xingyue Song	SCHS	2020	Reproductive period, years					cognitive decline	cohort	Singapore	8222	1332	7.5
			<35 years	OR	1.28	1.11	1.48				2193	477	
			35–39 years (ref.)	OR	1.00	1.00	1.00				4155	604	
			>39 years	OR	0.94	0.79	1.11				1874	251	
			Per 1-year increase	OR	0.97	0.96	0.98						
Joanne Ryan	ESPRIT	2009	Reproductive years	OR	0.98	0.96	1.01	cognitive decline	cohort	France	996	NA	4.5
Natalie L. Rasgon	HARMONY	2005	Reproductive period, years					cognitive decline	case-control	US	1111/4733	1111	6
			<35	OR	1.15	0.96	1.36				1407	276	
			35–39 (ref.)	OR	1.00	1.00	1.00				2310	399	
			>39	OR	0.82	0.66	1.00				1136	157	
Fu-Dong Li	ZPHS	2015	Reproductive period	OR	0.97	0.96	0.99	cognitive decline	case-control	CN	4,796	919	7
Hyesue Jang	KLOSCAD +HELIAD	2018	Reproductive period	OR	0.99	0.95	1.04	cognitive decline	case-control	Korea	3,549	896	5.5
Martin J. Prince	10/66 population-based	2018	Reproductive period	HR	1.00	0.99	1.02	dementia	cohort	Cuba, Dominican Republic, Puerto	8466	692	5.5

	cohort study									Rico, Venezuela, Mexico, Peru, China			
Jung Eun Yoo	NCSP	2020	Duration of fertility, years					dementia	cohort	Korea	4,696,633	212,227	7
			<30 (ref.)	HR	1.00	1.00	1.00				584182	45,408	
			30-34	HR	0.93	0.92	0.94				1831593	97,165	
			35-39	HR	0.81	0.80	0.82				1916595	57,242	
			≥ 40	HR	0.81	0.79	0.82				364263	12,412	
Paola Gilsanz	KPNC	2019	Reproductive span, years					dementia	cohort	US	6,137	2577	7
			<34.4 (mean)	HR	1.2	1.08	1.32				1,702		
			≥34.4 (ref.)	HR	1.00	1.00	1.00				2,345		
			≤20	HR	1.55	1.03	2.32				52		
			21–30	HR	1.26	1.08	1.49				687		
			31–34	HR	1.26	1.09	1.47				963		
			35–36	HR	1.02	0.87	1.19				784		
			37–38	HR	1.16	1.00	1.34				822		
			39–44 (ref.)	HR	1.00	1.00	1.00				739		
Paola Gilsanz	KPNC	2019	Reproductive span, years					dementia	cohort	US	6,137	2577	7
			<35.5 (mean)	HR	1.18	1.06	1.31				1,298		
			≥35.5 (ref.)	HR	1.00	1.00	1.00				1,893		
			<34.4	HR	1.27	1.13	1.42				1,012		
			≥34.4 (ref.)	HR	1.00	1.00	1.00				2,179		
			≤20	HR	1.61	0.97	2.67				26		
			21–30	HR	1.41	1.17	1.70				359		

			31–34	HR	1.31	1.12	1.54				627		
			35–36	HR	1.04	0.89	1.23				656		
			37–38	HR	1.16	1.00	1.35				785		
			39–44 (ref.)	HR	1.00	1.00	1.00				738		
Mirjam I. Geerlings	The Rotterdam Study	2001	Reproductive period, years					dementia	cohort	Netherlands	3588	199	6.5
			≤34 (ref.)	RR	1.00	1.00	1.00				333		
			34-36	RR	1.56	1.00	2.43				347		
			37-39	RR	1.64	1.07	2.53				320		
			≥39	RR	1.78	1.12	2.84				364		
			Per y increase	RR	1.04	1.01	1.08						
Jenna Najar	PPSW	2020	Reproductive period, years	HR	1.06	1.03	1.2	dementia	cohort	Sweden	1364	291	7
			Q1: <32.6 years (ref.)	HR	1.00	1.00	1.00				333		
			Q2: 33-35.7 years	HR	1.51	1.05	2.16				347		
			Q3: 36-37.4 years	HR	1.69	1.17	2.44				320		
			Q4: ≥38.0 years	HR	2.17	1.51	3.11				364		
A. Paganini-Hill	The 90+ Study	2020	Reproductive years					dementia	cohort	US	424	209	5.5
			≤32 (ref.)	HR	1.00	1.00	1.00				130	63	
			33–38	HR	1.06	0.76	1.47				150	81	
			39+	HR	0.84	0.59	1.2				140	64	
Jessica Gong	UK Biobank	2022	Reproductive period, years					dementia	cohort	UK	273,240		6.5
			<33 (ref.)	HR	1.00	1.00	1.00					186	
			33 to 35	HR	0.98	0.84	1.15					178	
			36 to 37	HR	0.78	0.67	0.92					166	
			38 to 39	HR	0.67	0.57	0.79					160	
			40 to 42	HR	0.68	0.58	0.79					192	

			>42	HR	0.8	0.67	0.95					137	
Jung Eun Yoo	NCSP	2020	Duration of fertility, years					AD	cohort	Korea	4,696,633	162,901	7
			<30 (ref.)	HR	1.00	1.00	1.00						
			30-34	HR	0.93	0.92	0.94						
			35-39	HR	0.81	0.80	0.82						
			≥ 40	HR	0.81	0.79	0.83						
Mirjam I. Geerlings	The Rotterdam Study	2001	Reproductive period, years					AD	cohort	Netherlands	3588	186	6.5
			≤34 (ref.)	RR	1.00	1.00	1.00					155	
			34-36	RR	1.32	0.81	2.14						
			37-39	RR	1.41	0.88	2.25						
			≥39	RR	1.51	0.91	2.5						
			Per y increase	RR	1.03	1.00	1.07						
Jenna Najar	PPSW	2020	Reproductive period, years	HR	1.06	1.02	1.11	AD	cohort	Sweden	1364	146	7
			Q1: <32.6 (ref.)	HR	1.00	1.00	1.00				333		
			Q2: 33-35.7	HR	1.81	1.07	3.07				347		
			Q3: 36-37.4	HR	1.91	1.11	3.28				320		
			Q4: ≥38.0	HR	2.78	1.65	4.71				364		
Hyesue Jang	KLOSCAD, HELIAD	2018	Reproductive period	OR	0.97	0.86	1.09	AD	case-control	Korea	3549	118	5.5

f) Characteristics of studies with cognitive decline, dementia, and AD by age at first birth.

**Author**	**Project**	**Year**	**Description of risk factor**	**RR/OR/ HR**	**Adjusted risk**	**LCI**	**UCI**	**Outcome**	**design**	**Country**	**Sample**	**Case**	**Score**
Yoko Shimizu	JPHC Study	2019	Age at first birth, years					cognitive decline	cohort	Japan	670	196	7.5
			≤ 22 (ref.)	OR	1.00	1.00	1.00				44	19	
			23-25	OR	0.78	0.40	1.52				290	99	
			≥ 26	OR	0.69	0.35	1.35				284	94	
Xingyue Song	SCHS	2020	Age at first livebirth, years					cognitive decline	cohort	Singapore	8222	1332	7.5
			<20 (ref.)	OR	1.00	1.00	1.00				1180	249	
			21–25	OR	1.06	0.88	1.28				3001	534	
			26–30	OR	0.92	0.74	1.15				2465	338	
			>30	OR	0.85	0.64	1.13				945	125	
			Per 5-year increase	OR	0.94	0.86	1.02						
Joanne Ryan	ESPRIT	2009	Age at birth of first child, years					cognitive decline	cohort	France	996	NA	4.5
			＜20 (ref.)	OR	1.00	1.00	1.00						
			21—29	OR	0.75	0.48	1.17						
			＞30	OR	0.91	0.54	1.54						
A. Paganini-Hill	The 90+ Study	2020	Age at first child, years					dementia	cohort	US	424	209	5.5
			≤20 (ref.)	HR	1.00	1.00	1.00				11	20	
			21–24	HR	0.88	0.51	1.51				46	40	
			25–29	HR	1.06	0.63	1.77				55	65	
			30+	HR	0.63	0.35	1.15				34	26	
Martin J. Prince	10/66 population-	2018	Age at birth of first child	HR	0.99	0.98	1.01	dementia	cohort	Cuba, Dominican Republic,	8466	692	5.5

	based cohort study									Puerto Rico, Venezuela, Mexico, Peru, China			
Jessica Gong	UK Biobank	2022	Age at first live birth, years					dementia	cohort	UK	273,240		6.5
			<21	HR	1.43	1.26	1.62					305	
			21 to 22	HR	1.23	1.08	1.4					270	
			23 to 24	HR	1.26	1.12	1.42					302	
			25 to 26 (ref.)	HR	1.00	1.00	1.00					217	
			27 to 29	HR	1.16	1.02	1.32					251	
			>29	HR	1.11	0.96	1.29					213	
			Per additional year of age	HR	0.98	0.97	1.00						
Alison Gemmill	HRS	2022	Younger age at first birth	HR	1.11	0.96	1.27	dementia	cohort	US	8,875	2,030	5

g) Characteristics of studies with cognitive decline, dementia, and AD by parity.

Author	Project	Year	Description of risk factor	RR/OR/HR	Adjusted risk	LCI	UCI	Outcome	design	Country	Sample	Case	Score
Yoko Shimizu	JPHC Study	2019	Number of births					cognitive decline	cohort	Japan	670	196	7.5
			1 (ref.)	OR	1.00	1.00	1.00				29	9	
			2	OR	1.19	0.50	2.79				228	87	
			3	OR	0.83	0.35	1.94				282	87	
			≥ 4	OR	1.03	0.41	2.63				83	31	
Xingyue Song	SCHS	2020	Number of children					cognitive decline	cohort	Singapore	8222	1332	7.5
			0	OR	1.02	0.70	1.48				624	84	
			1–2 (ref.)	OR	1.00	1.00	1.00				2586	324	
			3–4	OR	1.04	0.88	1.25				3457	495	
			5	OR	1.27	1.04	1.55				1555	429	
			Per 1 increase	OR	1.05	1.01	1.09						
Joanne Ryan	ESPRIT	2009	Nulliparous	OR	0.93	0.56	1.52	cognitive decline	cohort	France	996	NA	4.5
Joanne Ryan	ESPRIT	2009	Number of children					cognitive decline	cohort	France	996	NA	4.5
			0 (ref.)	OR	1.00	1.00	1.00						
			1	OR	1.52	0.82	2.81						
			2 or 3	OR	1.23	0.72	2.71						
			>3	OR	1.10	0.58	2.08						
Natalie L. Rasgon	HARMONY	2005	Parity					cognitive decline	case-control	US	5844	1111	6
			0	OR	1.09	0.90	1.31				783	194	
			1–2 (ref.)	OR	1.00	1.00	1.00				2529	546	
			3–4	OR	1.14	0.97	1.34				1223	298	

			≥5	OR	1.59	1.18	2.13				198	73	
Fu-Dong Li	ZPHS	2015	Number of full-term pregnancies					cognitive decline	retrospective	CN	4,796	919	7
			0	OR	1.03	0.54	1.94						
			1–4 (ref.)	OR	1.00	1.00	1.00						
			≥5	OR	1.36	1.13	1.63						
Hyesue Jang	HELIAD	2018	No. of completed pregnancy with childbirth					cognitive decline	retrospective	Korea	812	NA	5.5
			0	OR	1.74	0.76	4.03						
			1 (ref.)	OR	1.00	1.00	1.00						
			≥2	OR	0.99	0.64	1.53						
Hyesue Jang	KLOSCAD	2018	No. of completed pregnancy with childbirth					cognitive decline	retrospective	Korea	2737	NA	5.5
			0	OR	1.74	0.76	4.01						
			1 (ref.)	OR	1.00	1.00	1.00						
			2–4	OR	0.96	0.62	1.49						
			≥5	OR	1.07	0.67	1.69						
Martin J. Prince	10/66 population-based cohort study	2018	Parity (per child)	HR	1.03	1.00	1.06	dementia	cohort	Cuba, Dominican Republic, Puerto Rico, Venezuela, Mexico, Peru, China	8466	692	5.5
			Nulliparity	HR	1.16	0.86	1.56						
Jung Eun Yoo	NCSP	2020	Parity					dementia	cohort	Korea	4,696,633	212,227	7
			Nulliparity (ref.)	HR	1.00	1.00	1.00				103,671	2670	
			1	HR	0.89	0.85	0.94				363,216	6146	

			≥ 2	HR	1.04	0.99	1.08				4,229,746	203411	
Jenna Najar	PPSW	2020	Pregnancies (number)	HR	0.97	0.87	1.07	dementia	cohort	Sweden	1364	291	7
A. Paganini-Hill	The 90+ Study	2020	Number of children					dementia	cohort	US	424	209	5.5
			0	HR	1.00	1.00	1.00				118	52	
			1 (ref.)	HR	0.98	0.64	1.52				62	34	
			2	HR	1.23	0.86	1.75				152	76	
			3+	HR	1.18	0.79	1.76				87	45	
Jong Bin Bae	COSMIC	2020	0 (nulliparity)	OR	0.92	0.67	1.27	dementia	cohort	Multiple Regions	1368	84	5.5
			1 (ref.)	OR	1.00	1.00	1.00				2096	103	
			2	OR	1.13	0.86	1.47				3923	181	
			3	OR	1.19	0.91	1.57				2819	161	
			4	OR	1.07	0.79	1.45				1652	103	
			≥ 5 (grand multiparity)	OR	1.47	1.10	1.94				2061	241	
			5 to 9	OR	1.44	1.08	1.90				1836	212	
			≥ 10	OR	1.86	1.11	3.09				225	29	
Jessica Gong	UK Biobank	2022	Number of live births					dementia	cohort	UK	273,240		6.5
			0	HR	1.18	1.04	1.33					284	
			1	HR	1.09	0.95	1.25					218	
			2 (ref.)	HR	1.00	1.00	1.00					773	
			3	HR	1.03	0.93	1.15					392	
			4 or more	HR	1.14	0.98	1.33					190	
Alison Gemmill	HRS	2022	Parity					dementia	cohort	US	8,875	2,030	5
			0 (ref.)	HR	1.00	1.00	1.00						
			1	HR	0.96	0.78	1.18						
			2	HR	1.12	0.95	1.33						

			3	HR	0.98	0.82	1.17						
			>=4	HR	1.09	0.92	1.28						
Jung Eun Yoo	NCSP	2020	Parity					AD	cohort	Korea	4,696,633	162,901	7
			Nulliparity(ref.)	HR	1.00	1.00	1.00						
			1	HR	0.91	0.86	0.96						
			≥ 2	HR	1.05	1.00	1.10						
Jenna Najar	PPSW	2020	Pregnancies (number)	HR	0.96	0.84	1.11	AD	cohort	Sweden	1364	146	7
Hyesue Jang	KLOSCAD	2018	No. of completed pregnancy with childbirth					AD	case-control	Korea	2,737	NA	5.5
			0	OR	3.09	0.47	20.37						
			1 (ref.)	OR	1.00	1.00	1.00						
			2–4	OR	1.54	0.42	5.64						
			≥5	OR	2.54	0.70	9.26						
Hyesue Jang	HELIAD	2018	No. of completed pregnancy with childbirth					AD	case-control	Korea	812	NA	5.5
			0	OR	5.02	0.38	66.07						
			1 (ref.)	OR	1.00	1.00	1.00						
			≥2	OR	2.58	0.28	23.73						

h) Characteristics of studies with cognitive decline, dementia, and AD by miscarriage and stillbirth.

Author	Project	Year	Description of risk factor	RR/OR/HR	Adjusted risk	LCI	UCI	Outcome	design	Country	Sample	Case	Score
Hyesue Jang	KLOSCAD	2018	No. of incomplete pregnancy without childbirth					cognitive decline	case-control	Korea	2,737	NA	5.5
			0 (ref.)	OR	1.00	1.00	1.00						
			1	OR	1.02	0.81	1.29						
			≥2	OR	1.02	0.83	1.26						
Hyesue Jang	HELIAD	2018	No. of incomplete pregnancy without childbirth					cognitive decline	case-control	Korea	812	NA	5.5
			0 (ref.)	OR	1.00	1.00	1.00						
			1	OR	1.02	0.81	1.29						
			≥2	OR	1.02	0.82	1.26						
Fu-Dong Li	ZPHS	2015	Number of incomplete pregnancies					cognitive decline	case-control	CN	4,796	919	7
			0	OR	1.22	1.00	1.45						
			1–2 (ref.)	OR	1.00	1.00	1.00						
			≥3	OR	1.34	0.86	2.11						
Saima Basit,	NA	2019	Stillbirth	HR	1.71	1.18	2.50	dementia	cohort	Denmark	1243957	28	6
			≥2 miscarriages	HR	1.06	0.84	1.36					74	
			1 miscarriage	HR	0.98	0.86	1.11					402	
			No pregnancy loss (ref.)	HR	1.00	1.00	1.00					1684	
Jessica Gong	UK Biobank	2022	Number of miscarriages					dementia	cohort	UK	273,240		6
			0 (ref.)	HR	1.00	1.00	1.00					1,220	
			1	HR	0.90	0.78	1.03					249	
			2 or more	HR	1.02	0.83	1.25					106	
			Miscarriage versus not	HR	0.93	0.82	1.06					355	
			Per miscarriage	HR	1.01	0.94	1.08					-	

Ellika Andolf	n.a.	2020	Recurrent miscarriages	HR	0.94	0.54	1.61	dementia	cohort	Sweden	1128709	6,488	5.5
Hyesue Jang	KLOSCAD	2018	No. of incomplete pregnancy without childbirth					AD	case-control	Korea	2,737	NA	5.5
			0 (ref.)	OR	1.00	1.00	1.00						
			1	OR	0.45	0.24	0.84						
			≥2	OR	0.68	0.39	1.16						
Hyesue Jang	HELIAD	2018	No. of incomplete pregnancy without childbirth					AD	case-control	Korea	812	NA	5.5
			0 (ref.)	OR	1.00	1.00	1.00						
			1	OR	0.27	0.05	1.39						
			≥2	OR	0.18	0.04	0.86						
Saima Basit	NA	2019	Stillbirth	HR	1.61	0.86	3.01	AD	cohort	Denmark	1243957	10	6
			≥2 miscarriages	HR	1.20	0.84	1.71					33	
			1 miscarriage	HR	0.95	0.77	1.16					163	
			No loss (ref.)	HR	1.00	1.00	1.00					720	
Jessica Gong	UK Biobank	2022	Number of stillbirths					AD	cohort	UK	273,240		6
			0 (ref.)	HR	1.00	1.00	1.00					1,503	
			1	HR	1.15	0.88	1.49					65	
			2 or more	HR	1.27	0.66	2.45					12	
			Stillbirth versus not	HR	1.16	0.91	1.49					77	
			Per stillbirth	HR	1.10	0.92	1.31					-	

.

i) Characteristics of studies with cognitive decline, dementia, and AD by breastfeeding.

**Author**	**Project**	**Year**	**Description of risk factor**	**RR/OR/HR**	**Adjusted risk**	**LCI**	**UCI**	**Outcome**	**design**	**Country**	**Sample**	**Case**	**Score**
fu-Dong Li	ZAHCS	2020	Mean breastfeeding duration, month					cognitive decline	cohort	CN	5487	1053	7
			<6	OR	1.50	1.20	1.88						
			6-12	OR	1.58	1.29	1.93						
			12 (ref.)	OR	1.00	1.00	1.00						
			12–18	OR	1.33	1.06	1.68						
			18	OR	2.08	1.64	2.65						
Hyesue Jang	HELIAD	2018	Breastfed	OR	0.93	0.66	1.33	cognitive decline	case-control	Korea	812	NA	5.5
Yoko Shimizu	JPHC Study	2019	Breastfeeding					cognitive decline	cohort	Japan	670	196	7.5
			Never (ref.)	OR	1.00	1.00	1.00				65	27	
			Ever	OR	0.67	0.38	1.16				555	186	
Jung Eun Yoo	NCSP	2020	Duration of breast feeding months					dementia	cohort	Korea	4,696,633	212,227	7
			Never (ref.)	HR	1.00	1.00	1.00				383,752	6,825	
			< 6	HR	0.92	0.88	0.95				379,887	5,279	
			6 —12	HR	1.04	1.01	1.07				838,259	23,046	
			≥ 12	HR	1.14	1.11	1.17				3,094,735	177,077	
Jenna Najar	PPSW	2020	Breastfeeding, months	HR	1.00	0.98	1.01	dementia	cohort	Sweden	1364	291	7
Jung Eun Yoo	NCSP	2020	Duration of breast feeding months					AD	cohort	Korea	4,696,633	162,901	7
			Never (ref.)	HR	1.00	1.00	1.00						
			< 6	HR	0.89	0.86	0.93						
			6—12	HR	1.02	0.98	1.05						
			≥ 12	HR	1.11	1.08	1.15						

Jenna Najar	PPSW	2020	Breastfeeding, months	HR	0.99	0.97	1.02	AD	cohort	Sweden	1364	146	7
Hyesue Jang	HELIAD	2018	Breastfed	OR	0.83	0.3	2.32	AD	case-control	Korea	812	NA	5.5

2. Characteristics of studies with cognitive decline, dementia, and AD by gynecologic surgery.

a) Characteristics of studies with cognitive decline, dementia, and AD by surgical menopause.

Author	Project	Year	Description of risk factor	RR/OR/ HR	Adjusted risk	LCI	UCI	Outcome	design	Country	Sample	Case	Score
Yoko Shimizu	JPHC Study	2019	Menopausal type					cognitive decline	cohort	Japan	670	196	7.5
			Natural menopause (ref.)	OR	1.00	1.00	1.00				507	186	
			Surgical menopause	OR	0.89	0.53	1.49				79	27	
Joanne Ryan	ESPRIT	2009	Surgical menopause	OR	0.95	0.63	1.45	cognitive decline	cohort	France	996	NA	4.5
J Ryan	TCS	2014	Type of menopause					cognitive decline	cohort	France	4868	NA	7
			Natural Surgical (ref.)	HR	1.00	1.00	1.00						
			Surgical menopause	HR	1.13	0.90	1.43						
J Ryan	TCS	2014	Type of menopause					dementia	cohort	France	4868	393	7
			Natural Surgical (ref.)	HR	1.00	1.00	1.00						
			Surgical menopause	HR	0.83	0.51	1.36						
A. Paganini-Hill	The 90+ Study	2020	Menopause					dementia	cohort	US	424	209	5.5
			Natural (ref.)	HR	1.00	1.00	1.00				271	132	
			Surgical	HR	1.14	0.85	1.51				151	77	
Mirjam I. Geerlings	Rotterdam Study	2001	Artificial menopause	RR	1.17	0.72	1.89	dementia	cohort	Netherlands	3588	199	6.5
Mirjam I. Geerlings	Rotterdam Study	2001	Artificial menopause	RR	0.95	0.56	1.63	AD	cohort	Netherlands	3588	155	6.5

b) Characteristics of studies with cognitive decline, dementia, and AD by oophorectomy, hysterectomy, and hysterectomy with bilateral oophorectomy.

Author	Project	Year	Description of risk factor	RR/OR/HR	Adjusted risk	LCI	UCI	Outcome	design	Country	Sample	Case	Score
W.A. Rocca	MCOSOA	2007	Unilateral oophorectomy					cognitive decline	cohort	US	2961		7
			Referent women (ref.)	HR	1.00	1.00	1.00					98	
			Unilateral oophorectomy	HR	1.64	1.2	2.23					76	
Thien Kieu Thi Phung	DCRS	2010	Unilateral oophorectomy					dementia	cohort	Denmark	2,313,388		5
			Yes	RR	0.99	0.92	1.07					766	
			No (ref.)	RR	1.00	1.00	1.00					94,473	
W.A. Rocca	MCOSOA	2007	Bilateral oophorectomy					cognitive decline	cohort	US	2961		7
			Referent women	HR	1.00	1.00	1.00					98	
			Bilateral oophorectomy	HR	1.33	0.98	1.81					74	
W.A. Rocca	MCOSOA	2007	Overall					cognitive decline	cohort	US	2961		7
			Referent women	OR	1.00	1.00	1.00					98	
			Any oophorectomy	OR	1.46	1.13	1.90					150	
C. S. Uldbjerg	Danish Nurse Cohort	2022	Bilateral oophorectomy	RR	1.18	0.89	1.56	dementia	cohort	Danish	24,851	1238	5
			Unilateral oophorectomy	RR	0.87	0.59	1.23						
Thien Kieu Thi Phung	DCRS	2010	Bilateral oophorectomy					dementia	cohort	Denmark	2,313,388		5
			Yes	RR	1.07	1.01	1.14					2,248	
			No (ref.)	RR	1.00	1.00	1.00					92,991	
A. Paganini-Hill	The 90+ Study	2020	Bilateral oophorectomy	HR	1.14	0.75	1.72	dementia	cohort	US	424	209	5.5
Bushra Imtiaz	NA	2014	Oophorectomy (No history of malignant neoplasms)					AD	case-control	Finland	19,043 / 19,043	NA	6.5
			No (ref.)	OR	1.00	1.00	1.00						
			Yes	OR	0.85	0.75	0.97						

Bushra Imtiaz	NA	2014	Oophorectomy (History of malignant neoplasms)					AD	case-control	Finland	19,043 / 19,043	NA	6.5
			No (ref.)	OR	1.00	1.00	1.00						
			Yes	OR	3.00	0.20	44.87						
Thien Kieu Thi Phung	DCRS	2010	Overall					dementia	cohort	Denmark	2,313,388	95,239	5
			Hysterectomy										
			Yes	RR	0.91	0.84	0.98					3,534	
			No (ref.)	RR	1.00	1.00	1.00					91,705	
Paola Gilsanz	KPNC	2019	Hysterectomy					dementia	cohort	US	6,137	2577	7
			No (ref.)	HR	1.00	1.00	1.00				12,981		
			Yes	HR	1.08	1.01	1.16				2,773		
A. Paganini-Hill	The 90+ Study	2020	Surgical without bilateral oophorectomy	HR	1.24	0.85	1.81	dementia	cohort	US	424	209	5.5
Thien Kieu Thi Phung	DCRS	2010	Indications for hysterectomy					dementia	cohort	Denmark	2,313,388		5
			Unknown indication	RR	1.04	0.88	1.22					174	
			Benign	RR	1.06	0.99	1.15					2,161	
			Malignant (ref.)	RR	1.00	1.00	1.00					1,199	
			No hysterectomy (ref.)	RR	1.00	1.00	1.00					91,705	
Jessica Gong	UK Biobank	2022	Hysterectomy versus not	HR	1.12	1.01	1.25	dementia	cohort	UK	273,240	537	6.5
Bushra Imtiaz	NA	2014	Hysterectomy (History of malignant neoplasms)					AD	case-control	Finland	19,043 / 19,043	NA	6.5
			No (ref.)	OR	1.00	1.00	1.00						
			Yes	OR	3.00	0.20	44.87						
Bushra Imtiaz	NA	2014	Hysterectomy (No history of malignant neoplasms)					AD	case-control	Finland	19,043 / 19,043	NA	6.5
			No (ref.)	OR	1.00	1.00	1.00						
			Yes	OR	0.89	0.81	0.97						
Bushra Imtiaz	NA	2014	Hysterectomy with bilateral oophorectomy (History of malignant neoplasms)					AD		Finland		NA	6.5

			No (ref.)	OR	1.00	1.00	1.00		case-control		19,043 / 19,043		
			Yes	OR	3.00	0.20	44.87						
Bushra Imtiaz	NA	2014	Hysterectomy with bilateral oophorectomy (No history of malignant neoplasms)					AD	case-control	Finland	19,043 / 19,043	NA	6.5
			No (ref.)	OR	1.00	1.00	1.00						
			Yes	OR	0.85	0.75	0.98						

3. Characteristics of studies with cognitive decline, dementia, and AD by pregnancy-related diseases.

Author	Project	Year	Description of risk factor	RR/OR/ HR	Adjusted risk	LCI	UCI	Outcome	design	Country	Sample	Case	Score
Ellika Andolf	NA	2020	Pregnancy induced hypertension	HR	1.10	0.93	1.29	ACD	cohort	Sweden	1128709	6881	5.5
Ellika Andolf	NA	2020	Pregnancy induced hypertension	HR	0.98	0.82	1.17	dementia	cohort	Sweden	1128709	6488	5.5
M Nelander	SALT	2017	Pregnancy induced hypertension	HR	1.19	0.79	1.73	dementia	cohort	Sweden	3232	197	6.5
Ellika Andolf	NA	2020	Pregnancy induced hypertension	HR	1.24	0.92	1.65	AD	cohort	Sweden	1128709	2161	5.5
Ellika Andolf	NA	2020	Preeclampsia	HR	0.98	0.87	1.10	ACD	cohort	Sweden	1128709	6,881	5.5
Ellika Andolf	NA	2020	Preeclampsia	HR	0.90	0.80	1.02	dementia	cohort	Sweden	1128709	6488	5.5
Ellika Andolf	MBR, TPR	2007	Preeclampsia	HR	0.50	0.19	1.34	dementia	cohort	Sweden	283 902	608	7.5
Saima Basit	DCRS	2018	History of pre-eclampsia,	HR	1.26	1.03	1.54	dementia	cohort	Denmark	1 178 005	1728	7
Saima Basit	DCRS	2018	History of pre-eclampsia,	HR	1.38	1.00	1.91	AD	cohort	Denmark	1 178 005	676	7
Ellika Andolf	NA	2020	Preeclampsia	HR	1.01	0.81	1.26	AD	cohort	Sweden	1128709	2161	5.5
Ellika Andolf	NA	2020	Abruptio placenta	HR	0.85	0.61	1.19	ACD	cohort	Sweden	1128709	6881	5.5
Ellika Andolf	NA	2020	Placental anomaly	HR	1.07	0.58	2.38	ACD	cohort	Sweden	1128709	6881	5.5
Ellika Andolf	NA	2020	Fetal growth restriction (NPR)	HR	0.79	0.52	1.19	ACD	cohort	Sweden	1128709	6881	5.5
Ellika Andolf	NA	2020	Excessive growth (NPR)	HR	0.53	0.17	1.64	ACD	cohort	Sweden	1128709	6881	5.5
Ellika Andolf	NA	2020	Preterm labor and birth	HR	1.12	0.99	1.27	ACD	cohort	Sweden	1128709	6881	5.5
Ellika Andolf	NA	2020	PPROM	HR	1.14	1.00	1.29	ACD	cohort	Sweden	1128709	6881	5.5
Ellika Andolf	NA	2020	Gestational diabetes	HR	0.78	0.48	1.28	ACD	cohort	Sweden	1128709	6881	5.5
Ellika Andolf	NA	2020	Intrauterine fetal death	HR	1.15	0.62	2.14	ACD	cohort	Sweden	1128709	6881	5.5
Ellika Andolf	NA	2020	Infertility	HR	0.80	0.68	0.96	ACD	cohort	Sweden	1128709	6881	5.5

4. Characteristics of studies with cognitive decline, dementia, and AD by sex hormones in female.

a) Characteristics of studies with cognitive decline, dementia, and AD by estradiol in female.

**Author**	**Project**	**Year**	**Description of risk factor**	**RR/OR/HR**	**Adjusted risk**	**LCI**	**UCI**	**Outcome**	**design**	**Country**	**Sample**	**Case**	**Score**
Kristine Yaffe	SOF	1998	Quartiles of estradiol, pg/mL					cognitive decline	cohort	US	532	NA	3.5
			1 (<5） (ref.)	OR	1.00	1.00	1.00						
			2 (5-7)	OR	0.50	0.10	1.50						
			4 (10-25)	OR	1.00	0.40	2.70						
Mirjam I. Geerlings	Rotterdam Study	2003	Total estradiol tertiles, pmol/L					dementia	cohort	Netherlands	508	114	7.5
			≥0.0 and ＜7.1 (ref.)	HR	1.00	1.00	1.00					22	
			≥7.1 and ＜20	HR	1.58	0.75	3.35					27	
			≥20 and ≤67	HR	1.99	0.89	4.45					27	
			Per SD (14pmol/L) increase	HR	1.45	1.08	1.94					76	
Laure Carcaillon	3C	2014	Total-E2(estradiol), pg/mL					dementia	cohort	France	5262	132	7
			Q1: E2 ≤3.49	HR	2.20	1.07	4.52					41	
			Q2: 3.49–5.30	HR	1.46	0.68	3.15					26	
			Q3: 5.30–8.00 (ref.)	HR	1.00	1.00	1.00					25	
			Q4: E2 >8.00	HR	2.43	1.15	5.20					40	
Giovanni Ravaglia	CSBA	2007	Serum total estradiol, pg/mL					dementia	cohort	Italy	433	71	6
			Low (undetectable) (ref.)	HR	1.00	1.00	1.00				240		
			High (≥10)	HR	1.75	1.06	2.89				193		
Mirjam I. Geerlings	Rotterdam Study	2003	Total estradiol tertiles, pmol/L					AD	cohort	Netherlands	508		7.5
			≥0.0 and ＜7.1 (ref.)	HR	1.00	1.00	1.00					17	
			≥7.1 and ＜20	HR	1.58	0.70	3.55					22	
			≥20 and ≤67	HR	1.41	0.57	3.50					16	

			Per SD (14 pmol/L) increase	HR	1.30	0.88	1.91					55	
Laure Carcaillon	3C	2014	Total-E2(estradiol), pg/mL					AD	cohort	France	5262	90	7
			Q1: E2 ≤3.49	HR	2.07	0.89	4.75					28	
			Q2: 3.49–5.30	HR	1.37	0.57	3.35					18	
			Q3: 5.30–8.00 (ref.)	HR	1.00	1.00	1.00					17	
			Q4: E2 >8.00	HR	2.38	0.99	5.75					27	
Giovanni Ravaglia	CSBA	2007	Serum total estradiol, pg/mL					AD	cohort	Italy	433	46	6
			Low (undetectable) (ref.)	HR	1.00	1.00	1.00						
			High (≥10 pg/mL)	HR	1.94	1.04	3.61						
Kristine Yaffe	SOF	2010	Bioavailable oestradiol, pmol/L					cognitive decline	cohort	US	292	37	6.5
			Low1·9 (0·5–2·9) (ref.)	OR	1.00	1.00	1.00				97	15	
			Mid 4·0 (2·9–5·6)	OR	1.20	0.60	2.70				100	17	
			High 11·0 (5·7–38·9)	OR	0.30	0.10	1.00				94	5	
K. Yaffe	ABC	2006	Bioavailable estradiol tertile					cognitive decline	cohort	US	333	NA	5.5
			1	OR	3.90	1.60	9.60						
			2	OR	1.50	0.60	3.80						
			3 (ref.)	OR	1.00	1.00	1.00						
Mirjam I. Geerlings	Rotterdam Study	2003	Bioavailable estradiol tertiles, pmol/L					dementia	cohort	Netherlands	508		7.5
			≥0.0 and ＜4.7 (ref.)	HR	1.00	1.00	1.00					17	
			≥4.7 and ＜14	HR	1.47	0.59	3.63					18	
			≥14 and ≤49	HR	1.86	0.72	4.81					21	
			Per SD (11pmol/L) increase	HR	1.49	1.01	2.2					56	
Laure Carcaillon	3C	2014	Bio-E2, pg/mL					dementia	cohort	France	5262		7
			Q1: Bio-E2 ≤2.29	HR	1.95	0.99	3.89					44	
			Q2: 2.29–3.60	HR	0.98	0.42	2.27					24	

			Q3: 3.60–5.60 (ref.)	HR	1.00	1.00	1.00					25	
			Q4: Bio-E2 >5.60	HR	2.45	1.19	5.05					39	
Kristine Yaffe	SOF	2010	Non-protein-bound oestradiol, pg/mL					cognitive decline	cohort	US	292	37	6.5
			Low (ref.) 0·2 (0·1–0·3)	OR	1.00	1.00	1.00				106	17	
			Mid 0·4 (0·3–0·5)	OR	1.00	0.40	2.10				92	15	
			High 0·9 (0·6–4·5)	OR	0.30	0.10	0.90				94	5	

b) Characteristics of studies with cognitive decline, dementia, and AD by testosterone in female.

Author	Project	Year	Description of risk factor	RR/OR/ HR	Adjusted risk	LCI	UCI	Outcome	design	Country	Sample	Case	Score
Kristine Yaffe	SOF	2010	Total testosterone, ng/mL					cognitive decline	cohort	US	292	37	6.5
			Low 0·4 (0–0·5) (ref.)	OR	1.00	1.00	1.00				100	11	
			Mid 0·7 (0·5–0·8)	OR	0.90	0.40	2.30				99	11	
			High 1·3 (0·9–3·0)	OR	1.50	0.60	3.50				93	15	
Laure Carcaillon	3C	2014	Total testosterone, ng/mL					dementia	cohort	France	5262		7
			Q1: total-T ≤0.21	HR	1.33	0.69	2.58					42	
			Q2: 0.21–0.32	HR	1.07	0.55	2.07					25	
			Q3: 0.32–0.45	HR	1.02	0.52	1.97					27	
			Q4: Total-T >0.45 (ref.)	HR	1.00	1.00	1.00					27	
Laure Carcaillon	3C	2014	Total testosterone, ng/mL					AD	cohort	France	5262		7
			Q1: total-T ≤0.21	HR	1.15	0.51	2.61					90	
			Q2: 0.21–0.32	HR	1.05	0.47	2.33					25	
			Q3: 0.32–0.45	HR	1.52	0.71	3.28					16	
			Q4: Total-T >0.45 (ref.)	HR	1.00	1.00	1.00					23	
Kristine Yaffe	SOF	2010	Non-protein-bound testosterone					cognitive decline	cohort	US	292	37	6.5
			Low 4·4 (0–6·3) (ref.)	OR	1.00						101	12	
			Mid 8·5 (6·6–10·8)	OR	1.00	0.40	2.50				93	11	
			High 17·3 (11·1–40·3)	OR	1.20	0.50	2.80				98	14	
Giovanni Ravaglia	CSBA	2007	Serum free testosterone					dementia	cohort	Italy	433	71	6
			As a two-level variable										
			Low (≤1.3 pg/mL) (ref.)	HR	1.00	1.00	1.00						
			High (≥ 1.4 pg/mL)	HR	1.20	0.69	1.88						

			As a three-level variable, pg/mL									
			Low (＜1.1) (ref.)	HR	1.00	1.00	1.00					
			Intermediate (1.1–1.7)	HR	1.15	0.59	2.21					
			High (＞1.7)	HR	1.18	0.55	2.53					
Giovanni Ravaglia	CSBA	2007	Serum free testosterone, pg/mL					AD	cohort	Italy	433	46	6
			As a two-level variable									
			Low (≤1.3) (ref.)	HR	1.00	1.00	1.00					
			High (≥1.4)	HR	1.17	0.62	2.20					
			As a three-level variable, pg/mL									
			Low (＜1.1) (ref.)	HR	1.00	1.00	1.00					
			Intermediate (1.1–1.7)	HR	1.83	0.79	4.21					
			High (＞1.7)	HR	1.36	0.52	3.54					

c) Characteristics of studies with cognitive decline, dementia, and AD by estrone and SHBG in female.

Author	Project	Year	Description of risk factor	RR/OR/HR	Adjusted risk	LCI	UCI	Outcome	design	Country	Sample	Case	Score
Kristine Yaffe	SOF	1998	Quartiles of Estrone, pg/mL					cognitive decline	cohort	US	532	NA	3.5
			1（0-15）(ref.)	OR	1.00	1.00	1.00				142		
			2（16 -22）	OR	0.80	0.20	2.00				130		
			4（29-76）	OR	1.20	0.50	3.00				138		
Majon Muller	Manhattan	2010	SHBG per S.D. (31.6nmol/L)	HR	1.30	1.10	1.70	dementia	cohort	US	514	96	6

5. Characteristics of studies with cognitive decline, dementia, and AD by male-specific diseases.

**Author**	**Project**	**Year**	**Description of risk factor**	**RR/OR/ HR**	**Adjusted risk**	**LCI**	**UCI**	**Outcome**	**design**	**Country**	**Sample**	**Case**	**Score**
Chun-Ming Yang	NHIRD	2015	ED	HR	1.68	1.31	2.16	AD	cohort	CN	4153	82	6.5
			Non-ED (ref.)	HR	1.00	1.00	1.00					246	
			ED	HR	1.63	1.02	2.62	Non-AD			20,765	23	
			Non-ED (ref.)	HR	1.00	1.00	1.00					71	
Mette Norgaard	DNPR	2021	BPH	HR	1.16	1.10	1.21	AD(1-10 y)	cohort	Denmark	1404202	2572:7390	**7**
			BPH	HR	1.10	0.99	1.21	AD(10-21y)				1031:2556	
			BPH	HR	1.21	1.17	1.25	dementia (1-10 y)				5943:16124	
			BPH	HR	1.18	1.10	1.27	dementia (10-21 y)				1964:4621	
Bum Sik Tae	NHIS	2018	No ADT (ref.)	HR	1.00	1.00	1.00	cognitive decline	cohort	Korea	37549	4,743	5.5
			ADT＜12 months	HR	0.99	0.92	1.07						
			ADT≥12 months	HR	1.54	1.43	1.66						
N. Araújo	NEON-PC	2022	ADT	OR	3.71	1.31	10.59	cognitive decline	cohort	Portugal	366	NA	5
Hector Alonso-Quiñones	MCSA	2021	ADT	HR	1.25	0.75	2.1	cognitive decline	cohort	US	235	NA	8
Li-Ting Kao	LHID2005	2017	Patients receiving ADT	HR	1.21	0.82	1.78	dementia	cohort	CN	1314	NA	7
			Not receiving ADT (ref.)	HR	1.00	1.00	1.00						
			Patients receiving ADT										
			With GnRH agonists	HR	1.39	0.80	2.40						
			Without GnRH agonists	HR	1.13	0.75	1.71						

			Not receiving ADT (ref.)	HR	1.00	1.00	1.00						
Kevin T. Nead	SUHS	2016	No ADT use (ref.)	HR	1.00	1.00	1.00	dementia	cohort	US	9272	314	7
			ADT use	HR	2.21	1.72	2.83						
			ADT use, mo<12	HR	1.95	1.31	2.89						
			ADT use, mo≥12	HR	2.36	1.64	3.38						
Myungsun Shim	NHIS	2020	GnRHa use	HR	0.91	0.76	1.10	dementia	cohort	Korea	15324	354	6
			GnRHa use (Duration month)										
			≤12	HR	0.99	0.92	1.79						
			13-24	HR	1.32	0.91	1.5						
			25-36	HR	1.15	0.88	1.31						
			≥37	HR	1.27	1.02	1.65						
David Robinson	PCBaSe	2019	No prostate cancer (ref.)	HR	1.00	1.00	1.00	dementia	cohort	Sweden	146,985	7432	6
			watchful waiting.	HR	0.99	0.89	1.1					359	
			antiandrogen	HR	0.94	0.84	1.05					330	
			GnRH agonists	HR	1.15	1.07	1.23					841	
			Orchidectomy	HR	1.6	1.32	1.93					110	
Farzin Khosrow-Khavar	CPRD	2016	Nonuse Use (ref.)	HR	1.00	1.00	1.00	dementia	cohort	UK	15,593	275	7
			Use	HR	1.02	0.87	1.19				15,310	524	
			ADT type										
			GnRH agonists alone	HR	1.06	0.87	1.28					197	
			Oral antiandrogens alone	HR	1.01	0.76	1.33					64	
			GnRH agonists and oral antiandrogens	HR	1.02	0.85	1.22					249	
			Other types or combinations	HR	0.69	0.40	1.19					14	
			Duration of ADT use, months										

			＜6	HR	1.01	0.62	1.65					17	
			6--12	HR	0.84	0.62	1.13					51	
			13-18	HR	1.09	0.82	1.44					69	
Ravishankar Jayadevappa	SEER	2018	ADT	HR	1.20	1.17	1.24	dementia	cohort	US	154 089	8618	7
			No ADT (ref.)	HR	1.00	1.00	1.00					14 511	
			1-4 ADT doses	HR	1.19	1.15	1.23						
			5-8 ADT doses	HR	1.24	1.19	1.29						
			>8 ADT doses	HR	1.21	1.15	1.28						
			No ADT (ref.)	HR	1.00	1.00	1.00						
Anna Krasnova	SEER	2019	ADT	HR	1.17	1.07	1.27	dementia	cohort	US	100,414	3988	5
			ADT duration(months)										
			1--6	HR	1.04	0.94	1.14						
			≥7	HR	1.25	1.11	1.40						
			Dose continuous	HR	1.12	1.06	1.19						
Seo Hyon Baik	MBSF	2017	ADT treated	HR	1.01	1.01	1.02	dementia	cohort	US	1,238,879	109,815	5
			Prostatectomy treated	HR	0.67	0.66	0.68						
Jihun Kang	KNHIS	2020	Control (ref.)	HR	1.00	1.00	1.00	dementia	cohort	Korea	260919	9566	7.5
			AS/WW	HR	1.00	0.91	1.11					389	
			Surgery	HR	0.89	0.82	0.97					644	
			Surgery + ADT	HR	1.07	0.95	1.21					278	
			RT	HR	0.99	0.65	1.51					22	
			RT + ADT	HR	1.13	0.89	1.42					72	
			ADT	HR	1.21	1.14	1.28					1413	
Jong Won Kim	HIRA	2021	ADT use					dementia	cohort	Korea		NA	4
			No (ref.)	HR	1.00	1.00	1.00				102,973		

			Yes	HR	1.07	1.01	1.13				29,727		
			Duration of ADT (y)										
			No (ref.)	HR	1.00	1.00	1.00						
			<1	HR	1.12	1.01	1.25						
			1- 2	HR	1.32	1.20	1.47						
			2- 3	HR	1.09	0.97	1.21						
Peter E. Lonergan	CaPSURE	2022	ADT	HR	1.59	1.03	2.44	dementia	cohort	US	13,570	317	7
Jui-Ming Liu	NHID	2022	ADT-naïve group	HR	1.00	1.00	1.00	dementia	cohort	CN	8743	121	6
			ADT group	HR	1.12	0.87	1.43				8743	134	
			GnRH agonist-based ADT										
			ADT-naïve group	HR	1.00	1.00	1.00				8461	121	
			ADT group	HR	0.78	0.59	1.05				8461	75	
			Oral antiandrogens only										
			ADT-naïve group	HR	1.00	1.00	1.00				7087	114	
			ADT group	HR	1.18	0.93	1.49				7087	171	
			<6 months	HR	0.88	0.59	1.32				3792	37	
			6–12 months	HR	1.27	0.85	1.91				2987	41	
			13–18 months	HR	1.08	0.62	1.88				1928	19	
			19–24 months	HR	1.19	0.70	2.05				1807	23	
			>24 months	HR	0.59	0.40	0.85				5287	42	
	THIN		ADT-naïve group	HR	1.00	1.00	1.00	dementia	cohort	UK	14,949	237	6
			ADT group	HR	1.02	0.85	1.23				14,949	220	
			GnRH agonist-based ADT										
			ADT-naïve group	HR	1.00	1.00	1.00				14,440	236	

			ADT group	HR	0.99	0.83	1.20				14,440	212	
			Oral antiandrogens only										
			ADT-naïve group (ref.)	HR	1.00	1.00	1.00				2921	44	
			ADT group	HR	1.15	0.77	1.74				2921	48	
			<6 months	HR	1.14	0.93	1.41				10,577	161	
			6–12 months	HR	1.39	1.09	1.79				7661	117	
			13–18 months	HR	1.25	0.9	1.74				4040	73	
			19–24 months	HR	1.21	0.78	1.89				2158	44	
			>24 months	HR	0.68	0.52	0.91				4769	108	
S. D. Chung	LHID2000	2016	ADT	HR	1.76	0.55	5.62	AD	cohort	CN	1335	NA	6
Kevin T. Nead	SUMS	2016	ADT use	HR	2.04	1.23	3.40	AD	cohort	US	16888	NA	5
David Robinson	PCBaSe	2019	No prostate cancer (ref.)	HR	1.00	1.00	1.00	AD	cohort	Sweden	146,985	3342	6
			watchful waiting.	HR	1.01	0.86	1.18					169	
			antiandrogen	HR	0.97	0.83	1.14					163	
			GnRH agonists	HR	1.02	0.91	1.14					325	
			Orchidectomy	HR	1.33	0.96	1.84					37	
Ravishankar Jayadevappa	SEER	2018	ADT	HR	1.14	1.1	1.18	AD	cohort	US	154 089	8137	7
			1-4 ADT doses	HR	1.19	1.15	1.24						
			5-8 ADT doses	HR	1.28	1.22	1.35						
			>8 ADT doses	HR	1.24	1.16	1.34						
			No ADT (ref.)	HR	1.00	1.00	1.00						
Seo Hyon Baik	MBSF	2017	ADT	HR	1.08	1.07	1.09	AD	cohort	US	1,238,879	223,765	5
Jihun Kang	KNHIS	2020	Control (ref.)	HR	1.00	1.00	1.00	AD	cohort	7.5	260919	7169	7.5
			AS/WW	HR	1.05	0.93	1.17					307	
			Surgery	HR	0.90	0.82	0.98					478	

			Surgery + ADT	HR	1.05	0.91	1.21					201	
			RT	HR	1.12	0.7	1.78					18	
			RT + ADT	HR	0.99	0.74	1.32					46	
			ADT	HR	1.21	1.13	1.29					1064	
Jong Won Kim	HIRA	2021	ADT use					AD	cohort	Korea		NA	4
			No (ref.)	HR	1.00	1.00	1.00				102,973		
			Yes	HR	1.09	1.02	1.16				29,727		
			Duration of ADT (y)										
			No (ref.)	HR	1.00	1.00	1.00						
			<1	HR	1.10	0.98	1.24						
			1 2	HR	1.34	1.20	1.51						
			2 3	HR	1.17	1.03	1.32						
			≥3	HR	0.98	0.90	1.06						

6. Characteristics of studies with cognitive decline, dementia, and AD by sex hormones in male.

a) Characteristics of studies with cognitive decline, dementia, and AD by estradiol in male.

**Author**	**Project**	**Year**	**Description of risk factor**	**RR/OR/ HR**	**Adjusted risk**	**LCI**	**UCI**	**Outcome**	**design**	**Country**	**Sample**	**Case**	**Score**
Majon Muller	Zoetermeer	2008	Total estradiol (pmol/L)1 S.D. increase	OR	1.60	1.00	2.50	cognitive decline	cohort	Netherlands	218	44	7
Laure Carcaillon	3C	2013	Total estradiol					dementia	cohort	France	503	104	6
			1-SD decrease	HR	0.90	0.67	1.05				501	104	
			1	HR	0.92	0.47	1.79				162	33	
			2	HR	0.72	0.35	1.47				166	32	
			3 (ref.)	HR	1.00	1.00	1.00				173	39	
Andrew H	HIMS	2018	Estradiol (per SD decrease)	HR	1.11	1.01	1.22	dementia	cohort	Australia	4069	499	6
			Q1	HR	1.44	1.10	1.90						
			Q2	HR	1.24	0.93	1.66						
			Q3	HR	1.33	1.00	1.76						
			Q4 (ref.)	HR	1.00	1.00	1.00						
Mirjam I. Geerlings	Rotterdam Study	2003	Total estradiol tertiles, pmol/L					dementia	cohort	Netherlands	438	71	7.5
			≥0.0 and ＜35 (ref.)	HR	1.00	1.00	1.00					24	
			≥35 and ＜53	HR	0.45	0.18	1.16					13	
			≥53 and ≤157	HR	1.16	0.52	2.58					16	
			Per SD (24pmol/L) increase	HR	1.10	0.69	1.76					53	
Giovanni Ravaglia	CSBA	2007	Serum total estradiol					dementia	cohort	Italy	376	39	6
			As a two-level variable, pg/mL										
			Low (≤ 22) (ref.)	HR	1.00	1.00	1.00						
			High (≥23)	HR	1.00	0.51	1.93						

			As a three-level variable, pg/mL										
			Low (＜14) (ref.)	HR	1.00	1.00	1.00						
			Intermediate (14–34)	HR	1.37	0.57	3.29						
			High (＞34)	HR	0.81	0.28	2.34						
Mirjam I. Geerlings	Rotterdam Study	2003	Total estradiol tertiles, pmol/L					AD	cohort	Netherlands	438		7.5
			≥0.0 and ＜35 (ref.)	HR	1.00	1.00	1.00					15	
			≥35 and ＜53	HR	0.68	0.25	1.88					11	
			≥53 and ≤157	HR	0.82	0.29	2.30					7	
			Per SD (24 pmol/L) increase	HR	0.8	0.47	1.37					33	
Laure Carcaillon	3C	2013	Total estradiol: Tertiles					AD	cohort	France	413+ 90 dementia	62	6
			1-SD decrease	HR	1.13	0.82	1.55						
			1	HR	1.47	0.65	3.30						
			2	HR	1.03	0.40	2.64						
			3 (ref.)	HR	1.00	1.00	1.00						
Giovanni Ravaglia	CSBA	2007	Serum total estradiol					AD	cohort	Italy	376	23	6
			As a two-level variable, pg/mL										
			Low (≤ 22) (ref.)	HR	1.00	1.00	1.00						
			High (≥23)	HR	0.91	0.38	2.18						
			As a three-level variable, pg/mL										
			Low (＜14) (ref.)	HR	1.00	1.00	1.00						
			Intermediate (14–34)	HR	0.94	0.32	2.79						
			High (＞34)	HR	0.59	0.15	2.37						
Mirjam I. Geerlings	Rotterdam Study	2003	Bioavailable estradiol tertiles, pmol/L					dementia	cohort	Netherlands	438		7.5
			≥0.0 and ＜27 (ref.)	HR	1.00	1.00	1.00					20	
			≥27 and ＜41	HR	0.65	0.23	1.87					11	

			≥41 and ≤130	HR	0.91	0.36	2.28					10	
			Per SD (18pmol/L) increase	HR	1.11	0.63	1.95					41	
Mirjam I. Geerlings	Rotterdam Study	2003	Bioavailable estradiol tertiles, pmol/L					AD	cohort	Netherlands	438		7.5
			≥0.0 and ＜27 (ref.)	HR	1.00	1.00	1.00					13	
			≥27 and ＜41	HR	0.85	0.25	2.90					9	
			≥41 and ≤130	HR	0.43	0.13	1.47					3	
			Per SD (18 pmol/L) increase	HR	0.74	0.37	1.48					25	
Mirjam I. Geerlings	HAA	2006	Bioavailable estradiol	HR	1.12	0.95	1.33	AD	cohort	Netherlands	2300	134	7
Majon Muller	Zoetermeer	2008	Free estradiol, pmol/L	OR	1.50	1.00	2.40	cognitive decline	cohort	Netherlands	218	44	7
Erin S. LeBlanc	MrOS	2010	Per SD change in free oestradiol	HR	1.15	0.99	1.35	cognitive decline	cohort	US	1001	NA	6

b) Characteristics of studies with cognitive decline, dementia, and AD by testosterone in male.

**Author**	**Project**	**Year**	**Description of risk factor**	**RR/OR/ HR**	**Adjusted risk**	**LCI**	**UCI**	**Outcome**	**design**	**Country**	**Sample**	**Case**	**Score**
Majon Muller	Zoetermeer	2008	Total testosterone nmol/L	OR	1.00	0.70	1.40	cognitive decline	cohort	Netherlands	218	44	7
Sri Suravarapu	NA	2006	Testosterone, ng/mL					dementia	cohort	US	128	21	5
			<410	HR	2.09	0.76	5.75						
			≥410 (ref.)	HR	1.00	1.00	1.00						
			Continuous (100 ng/mL increase)	HR	0.95	0.72	1.26						
Laure Carcaillon	3C	2013	Total testosterone: tertiles					dementia	cohort	France	503		6
			1-SD decrease	HR	1.16	0.87	1.54				503	105	
			1	HR	2.33	1.11	4.90				171	44	
			2 (ref.)	HR	1.00	1.00	1.00				156	23	
			3	HR	1.91	0.83	4.38				176	38	
Andrew H	HIMS	2018	Total testosterone (per SD decrease)	HR	1.14	1.03	1.26	dementia	cohort	Australia	4069	499	6
			Q1	HR	1.39	1.04	1.85						
			Q2	HR	1.31	1.00	1.73						
			Q3	HR	1.23	0.93	1.61						
			Q4 (ref.)	HR	1.00	1.00	1.00						
Margot J Overman	UKB	2022	Total testosterone, tertiles					dementia	cohort	UK			6.5
			Q1 (lowest T)	HR	1.43	1.13	1.81				31901	208	
			Q2	HR	1.18	0.94	1.48				31871	155	
			Q3	HR	1.00	0.78	1.28				31894	134	

			Q4	HR	1.03	0.90	1.18				31863	171	
			Q5	HR	1.00	1.00	1.00				31882	158	
Laure Carcaillon	3C	2013	Total testosterone, tertiles					AD	cohort	France	503	62	6
			1-SD decrease	HR	1.30	0.95	1.76						
			1	HR	3.34	1.20	9.29						
			2 (ref.)	HR	1.00	1.00	1.00						
			3	HR	2.43	0.75	7.9						
S.D. Moffat	BLSA	2004	Total testosterone	HR	1.02	0.98	1.06	AD	cohort	US	574	54	6.5
Margot J Overman	UKB	2022	Total testosterone, tertiles					AD	cohort	UK			6.5
			Q1 (lowest T)	HR	1.80	1.21	2.66				31901	67	
			Q2	HR	1.51	1.04	2.21				31871	63	
			Q3	HR	1.08	0.72	1.64				31894	52	
			Q4	HR	1.07	0.85	1.35				31863	54	
			Q5 (ref.)	HR	1.00	1.00	1.00				31882	52	
Sri Suravarapu	NA	2006	Bioavailable testosterone, ng/mL					dementia	cohort	US	128	21	5
			<12	HR	1.22	0.38	3.87						
			≥12 (ref.)	HR	1.00	1.00	1.00						
			Continuous (5 ng/mL increase)	HR	1.11	0.63	1.97						
Laure Carcaillon	3C	2013	Bioavailable testosterone, tertiles					dementia	cohort	France	503		6
			1-SD decrease	HR	1.29	1.03	1.62				398	105	
			1	HR	2.20	1.02	4.73				130	44	
			2	HR	1.44	0.69	3.03				135	35	
			3 (ref.)	HR	1.00	1.00	1.00				133	26	

Mirjam I. Geerlings	HAA	2006	Bioavailable testosterone	HR	1.19	1.02	1.38	AD	cohort	Netherlands	2300	134	7
Laure Carcaillon	3C	2013	Bioavailable testosterone: tertiles					AD	cohort	France	503	62	6
			1-SD decrease	HR	1.46	1.14	1.87						
			1	HR	2.9	1.12	7.55						
			2	HR	1.91	0.69	5.32						
			3 (ref.)	HR	1.00	1.00	1.00						
Majon Muller	Zoetermeer	2008	Free testosterone, nmol/L	OR	0.90	0.60	1.30	cognitive decline	cohort	Netherlands	218	44	7.5
Erin S. LeBlanc	MrOS	2010	Per SD change in free testosterone	HR	1.07	0.87	1.31	cognitive decline	cohort	US	1001	NA	6
Giovanni Ravaglia	CSBA	2007	Serum free testosterone, pg/mL					dementia	cohort	Italy	376	39	6
			Low (≤ 8.1) (ref.)	HR	1.00	1.00	1.00						
			High (≤ 8.2)	HR	1.40	0.72	2.73						
Andrew H	HIMS	2018	Free testosterone (per SD decrease)	HR	1.18	1.06	1.31	dementia	cohort	Australia	4069	499	6
			Q1	HR	1.43	1.08	1.90						
			Q2	HR	1.32	1.00	1.74						
			Q3	HR	1.05	0.79	1.20						
			Q4 (ref.)	HR	1.00	1.00	1.00						
S.D. Moffat	BLSA	2004	Free testosterone index(nMol/nMol)	HR	1.06	0.88	1.29	dementia	cohort	US	574	68	6.5
S.D. Moffat	BLSA	2004	Free testosterone index(nMol/nMol)	HR	0.74	0.57	0.96	AD	cohort	US	574	54	6.5
	CSBA	2007	Serum free testosterone, pg/mL					AD	cohort	Italy	376	23	6

Giovanni Ravaglia			Low (≤ 8.1) (ref.)	HR	1.00	1.00	1.00						
			High (≤ 8.2)	HR	1.13	0.47	2.71						

c) Characteristics of studies with cognitive decline, dementia, and AD by estrone, SHBG, LH, dihydrotestosterone in male.

**Author**	**Project**	**Year**	**Description of risk factor**	**RR/OR/ HR**	**Adjusted risk**	**LCI**	**UCI**	**Outcome**	**design**	**Country**	**Sample**	**Case**	**Score**
Majon Muller	Zoetermeer	2008	Estrone, pmol/L	OR	1.20	0.80	1.80	cognitive decline	cohort	Netherlands	218	44	7
Erin S. LeBlanc	MrOS	2010	per SD change in SHBG	HR	1.25	1.06	1.49	cognitive decline	cohort	US	1001	NA	6
Andrew H	HIMS	2018	SHBG (per SD decrease)	HR	1.08	0.98	1.18	dementia	cohort	Australia	4069	499	6
			Q1(ref.)	HR	1.00	1.00	1.00						
			Q2	HR	1.07	0.79	1.44						
			Q3	HR	1.27	0.95	1.68						
			Q4	HR	1.27	0.95	1.71						
Majon Muller	Manhattan	2010	SHBG per S.D.(22.9nmol/L)	HR	1.20	0.90	1.80	dementia	cohort	US	217	50	6
Majon Muller	Manhattan	2010	SHBG per S.D.(22.9nmol/L)	HR	1.30	1.00	2.00	AD	cohort	US	217	33	6
Andrew H	HIMS	2018	Luteinizing hormone (per SD decrease)	HR	1.10	1.01	1.20	dementia	cohort	Australia	4069	499	6
			Q1 (ref.)	HR	1.00	1.00	1.00						
			Q2	HR	1.01	0.76	1.32						
			Q3	HR	0.96	0.73	1.26						
			Q4	HR	1.08	0.82	1.41						
Margot J Overman	UKB	2022	SHBG					dementia	cohort	UK			6.5
			Q1	HR	0.66	0.51	0.85				29,516	125	
			Q2	HR	0.70	0.55	0.89				29,523	124	
			Q3	HR	0.67	0.52	0.85				29,508	120	
			Q4	HR	0.76	0.66	0.88				29,513	175	

			Q5 (ref.)	HR	1.00	1.00	1.00				29,500	224	
Margot J Overman	UKB	2022	SHBG					AD	cohort	UK			6.5
			Q1	HR	0.53	0.34	0.84				29,516	38	
			Q2	HR	0.72	0.49	1.07				29,523	43	
			Q3	HR	0.66	0.44	0.99				29,508	46	
			Q4	HR	0.75	0.59	0.95				29,513	64	
			Q5 (ref.)	HR	1.00	1.00	1.00				29,500	79	
Andrew H	HIMS	2018	Dihydrotestosterone (per SD decrease)	HR	1.09	0.97	1.20	dementia	cohort	Australia	4069	499	6
			Q1	HR	1.20	0.90	1.59						
			Q2	HR	1.22	0.93	1.59						
			Q3	HR	1.07	0.81	1.40						
			Q4 (ref.)	HR	1.00	1.00	1.00						
Majon Muller	Zoetermeer	2008	Estrone (pmol/L)	OR	1.20	0.80	1.80	cognitive decline	cohort	Netherlands	218	44	7

Abbravations:ACD: all cause dementia, AD: Alzheimer’s disease, ADT: androgen deprivation therapy, BPH: benign prostate hyperplasia, DHEA: Dehydroepiandrosterone, E: estradiol, GnRHa: gonadotropin-releasing hormone agonist, HR, hazard ratios, HT: hormone therapy, LH-RH: luteinizing hormone releasing hormone, NPR: fetal growth restriction, OR: odd ratio, PPROM: premature rupture of membranes, RT: radiotherapy, SD: standard deviation, SHBG: sex hormone binding globulin, SLE: systemic lupus erythematosus, T: testosterone, WW: watchful waiting.

Appendix H Results of dose-response analysis between certain factors and relative risks of dementia or cognitive decline.

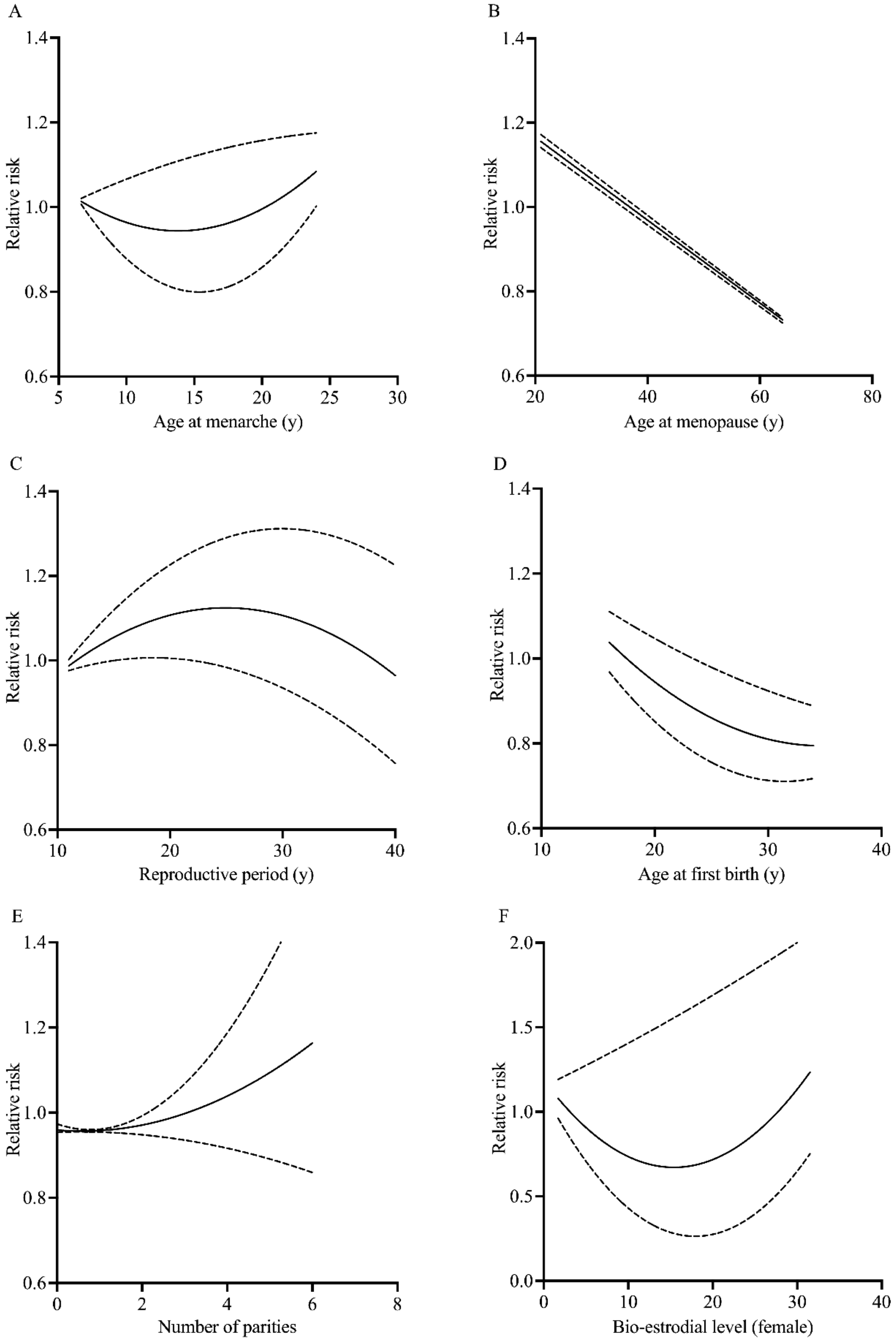

G

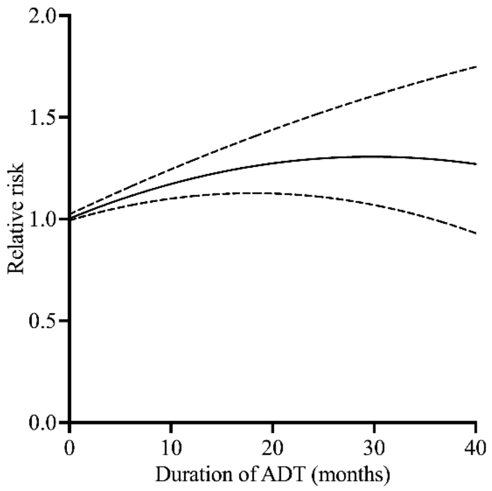
2.0
1.5
1.0
0.5
0.0
Relative risk
0
10
20
30
40
Duration of ADT (months)

Appendix I Results of subgroup analysis on ADT.

1. Subgroup analysis between races.

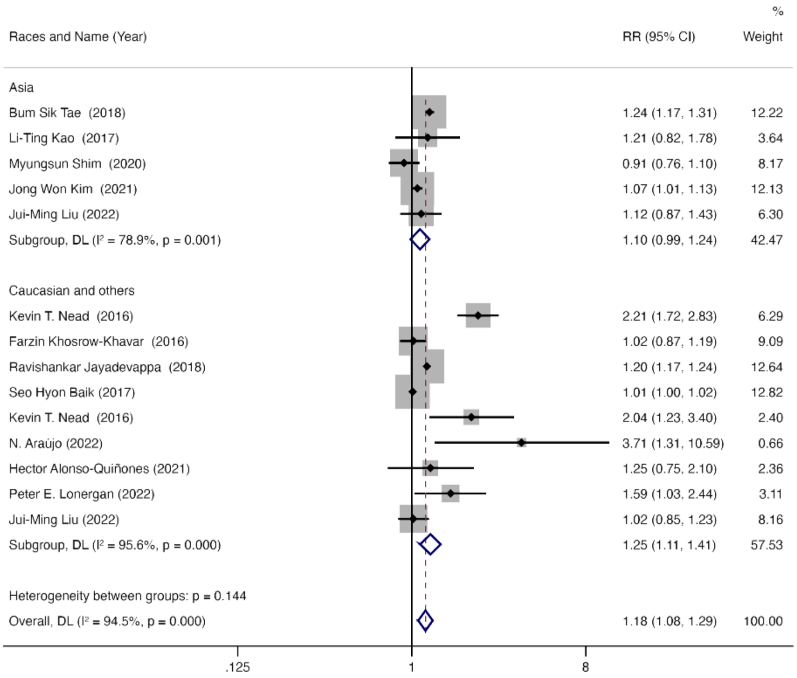

2. Subgroup analysis between mean follow-up years.

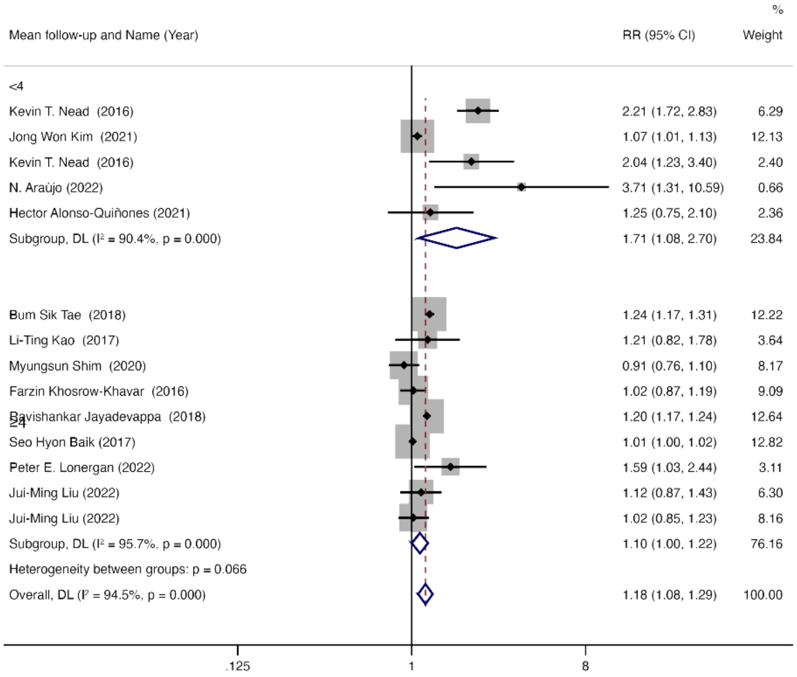

3. Subgroup analysis between sample sizes.

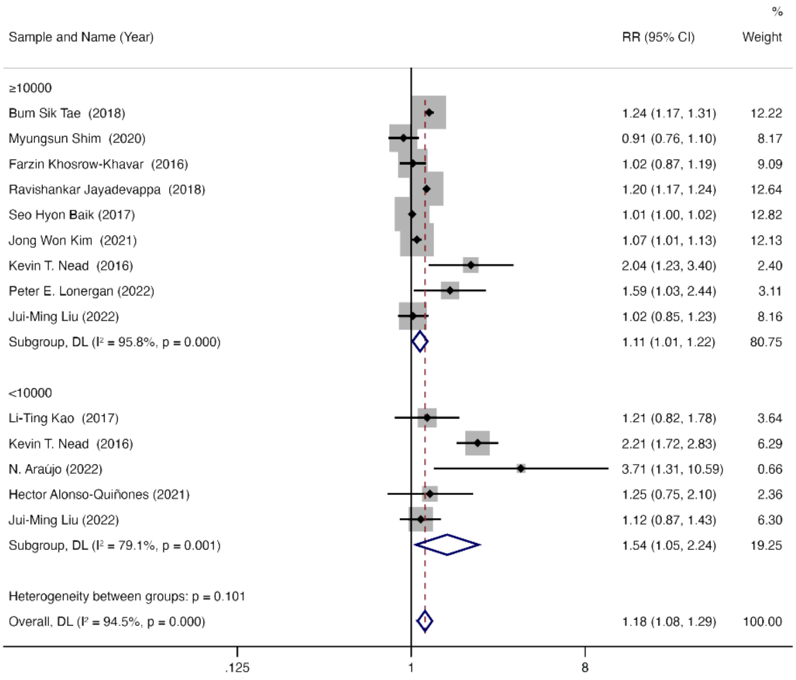

4. Subgroup analysis between NOS scores.

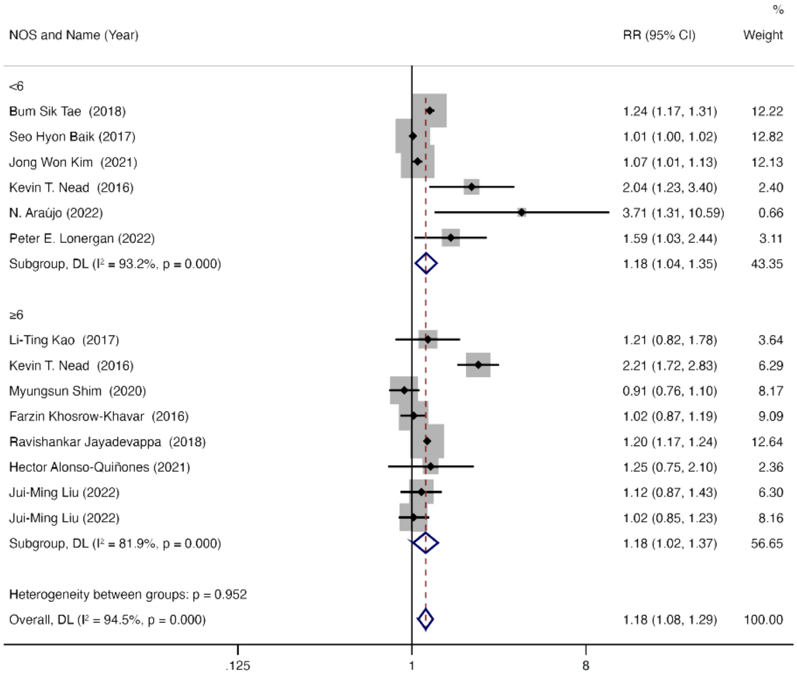

Appendix J Detailed GRADE score on credibility of the meta-analysis on dementia or cognitive decline.

Risk factors	No.	Sample	RR (95%CI)	Effect size	ROB	Inconsist.	Imprecis.	Indirect.	Pub. bias	GEADE certainty of evidence
Early menarche	8	4,721,518	1.04 (0.94 - 1.11)		🟡	🟢	🟢	🟡	🟡	⊕○○○ Very low
Late menarche	4	4,711,662	1.15 (1.14 - 1.17)		🟡	🟢	🟢	🟡	🟡	⊕○○○ Very low
Age at menarche (per year)	5	22,597	1.01 (0.98 - 1.04)		🔴	🟢	🟢	🟡	🟡	⊕○○○ Very low
Early menopause	7	4,722,798	1.22 (1.11 - 1.34)		🟡	🔴	🟡	🟡	🟡	⊕○○○ Very low
Late menopause	5	4,984,363	0.93 (0.91 - 0.96)		🟡	🟢	🟢	🟡	🟡	⊕○○○ Very low
Age at menopause (per year)	7	24,883	0.99 (0.97 - 1.02)		🔴	🔴	🟢	🟡	🟡	⊕○○○ Very low
Short reproductive period	8	307,531	1.14 (1.05 - 1.24)		🟡	🔴	🟢	🟡	🟡	⊕○○○ Very low
Long reproductive period	7	293,352	0.91 (0.83 - 0.99)		🟡	🟢	🟢	🟡	🟡	⊕○○○ Very low
Reproductive period (per year)	4	20,155	0.99 (0.97 - 1.02)		🔴	🔴	🟢	🟡	🟡	⊕○○○ Very low
Early childbearing	5	291,757	1.07 (0.96 - 1.19)		🔴	🟢	🟢	🟡	🟡	⊕○○○ Very low
Late childbearing	4	282,882	1.03 (0.88 - 1.22)		🟡	🟢	🟢	🟡	🟡	⊕○○○ Very low

Nulliparous	10	4,752,597	1.11 (1.06 - 1.16)							⊕◯◯◯ Very low
Grand multiparity	5	37,203	1.31 (1.18 - 1.47)							⊕◯◯◯ Very low
Number of parities	3	18,052	1.03 (1.01 - 1.05)							⊕◯◯◯ Very low
1 miscarriage	3	1,520,746	0.95 (0.87 - 1.03)							⊕◯◯◯ Very low
≥2 miscarriages	5	2,654,251	1.01 (0.91 - 1.11)							⊕◯◯◯ Very low
Breastfeeding	3	4,698,115	1.10 (0.96 - 1.26)							⊕◯◯◯ Very low
Breastfeeding duration	3	4,703,484	1.04 (0.86 - 1.25)							⊕◯◯◯ Very low
Surgical menopause	5	10,546	1.07 (0.93 - 1.23)							⊕◯◯◯ Very low
Bilateral oophorectomy	5	2,379,710	1.08 (1.02 - 1.15)							⊕◯◯◯ Very low
Hysterectomy	5	2,631,275	1.01 (0.91 - 1.12)							⊕◯◯◯ Very low
Any oophorectomy	3	314,287	1.08 (0.82 - 1.41)							⊕◯◯◯ Very low
Unilateral oophorectomy	3	2,341,200	1.12 (0.81 - 1.55)							⊕◯◯◯ Very low
Preeclampsia	3	1,128,709	0.85 (0.61 - 1.19)							⊕◯◯◯ Very low

Total estradiol in female	5	6,704	1.26 (0.69 - 2.30)							⊕◯◯◯ Very low
Bioavailable estradiol in female	4	6,395	0.70 (0.27 - 1.78)							⊕◯◯◯ Very low
ADT*	13	2,727,291	1.18 (1.08 - 1.29)							⊕◯◯◯ Very low
GnRH agonist*	4	70,442	0.97 (0.86 - 1.09)							⊕◯◯◯ Very low
Total estradiol in male	5	5,604	0.98 (0.71 - 1.37)							⊕◯◯◯ Very low
Total testosterone in male	6	164,913	0.88 (0.74 - 1.04)							⊕◯◯◯ Very low
Bioavailable testosterone in male	4	7,000	0.93 (0.67 - 1.29)							⊕◯◯◯ Very low
Free testosterone in male	5	6,238	0.97 (0.81 - 1.17)							⊕◯◯◯ Very low
SHBG in male	5	165,272	1.22 (1.06 - 1.39)							⊕◯◯◯ Very low

= high concerns = some concerns = no concerns

= small (0.75＜RR＜1.25) = general (1.25≤RR＜2) or (0.5＜RR≤0.75) = large (2≤RR＜5) or (0.2＜RR≤0.5)

Note: High certainty: We are very confident that the true effect lies close to that of the estimate of the effect. Moderate certainty: We are moderately confident in the effect estimate--the true effect is likely to be close to the estimate of the effect, but there is a possibility that it is substantially different. Low certainty: Our confidence in the effect estimate is limited- the true effect may be substantially different from the estimate of the effect.

Abbreviations: RR, adjusted relative risk; No, number of studies in pooled meta-analysis for each outcome; ROB, risk of bias; Inconsist, inconsistency; Imprecis, imprecision; Indirect, indirectness; Pub. Bias, publication bias.

Appendix K Results of the systemic reviews.

1. Results of the systemic reviews on female-specific reproductive factors.

Factors	Author	Year	Fits with the results of meta-analysis or not	NOS
Increased total-E2	Elizabeth Barrett-Connor	1999	No	5.5
	Joanne Ryan	2012	No	5.5
	Gail A. Laughlin	2010	No	5.5
	Kristine Yaffe	1998	No	3.5
	Mirjam I. Geerlings	2002	Yes	7.5
	Laure Carcaillon	2014	No	7
	Giovanni Ravaglia	2007	Yes	6
Increased bio-E2	Elizabeth Barrett-Connor	1999	Yes	5.5
	Gail A. Laughlin	2010	No	5.5
	Kristine Yaffe	2010	Yes	6.5
	K. Yaffe	2006	Yes	5.5
	Mirjam I. Geerlings	2002	No	7.5
	Laure Carcaillon	2014	Yes	7
Increased estrone	Elizabeth Barrett-Connor	1999	Yes	5.5
	Joanne Ryan	2012	Yes	5.5
	Kristine Yaffe	1998	Yes	3.5
Increased total-T	Elizabeth Barrett-Connor	1999	Yes	5.5
	Joanne Ryan	2012	Yes	5.5
	Kristine Yaffe	2010	Yes	6.5
	Laure Carcaillon	2014	Yes	7
Increased free E2	Joanne Ryan	2012	Yes	5.5
	Kristine Yaffe	2010	Yes	6.5
(Pre)eclampsia	Ineke R. Postma	2016	Yes	4
	Ellika Andolf	2020	Yes	5.5
	Ellika G Andolf	2007	Yes	7.5
	Saima Basit	2018	No	7
	Lina Bergman	2021	Yes	5
Nulliparious	Sanna L. Read	2017	Yes	6
	Emily W. Harville	2019	Yes	4.5
	Xingyue Song	2020	No	7.5
	Joanne Ryan	2009	No	4.5
	Natalie L. Rasgon	2005	No	6
	Fu-Dong Li	2015	No	6
	Hyesue Jang	2018	Yes	5.5
	Martin J. Prince	2018	No	5.5
	A. Paganini-Hill	2020	No	5.5
	Jong Bin Bae	2020	No	5.5
	Jung Eun Yoo	2020	Yes	7
	Joseph L. Saenz	2021	Yes	5

Early age at first birth	Emily W. Harville	2019	Yes	4.5
	Xingyue Song	2020	Yes	7.5
	Joanne Ryan	2009	Yes	4.5
	A. Paganini-Hill	2020	Yes	5.5
	Jessica Gong	2022	No	6.5
	Alison Gemmill	2022	Yes	5.5
Fetal growth restriction	Emily W. Harville	2019	Yes	4.5
	Ellika Andolf	2020	Yes	5.5
Preterm birth	Emily W. Harville	2019	Yes	4.5
	Ellika Andolf	2020	Yes	5.5
Gestational diabetes	Emily W. Harville	2019	Yes	4.5
	Ellika Andolf	2020	Yes	5.5
Pregnancy induced hypertension	Emily W. Harville	2019	Yes	4.5
	Carolien N.H. Abheiden	2015	Yes	4.5
	Michelle M. Mielke	2016	No	5
	Ellika Andolf	2020	Yes	5.5
	M Nelander	2017	Yes	6.5
Miscarriage	Emily W. Harville	2019	Yes	4.5
	Sujarwoto Sujarwoto	2019	Yes	6
	Hyesue Jang	2018	Yes	5.5
	Saima Basit,	2019	Yes	6
	Jessica Gong	2022	Yes	6.5
Breastfeeding (ever)	Emily W. Harville	2019	No	4.5
	Hyeue Jang	2018	Yes	5.5
	Yoko Shimizu	2019	Yes	7
	Jung Eun Yoo	2020	Yes	7
Reproductive period (per year)	Hung-Tse Chou	2021	Yes	5
	L.-F. Low	2005	Yes	6
	Xingyue Song	2020	Yes	7.5
	Fu-Dong Li	2015	Yes	6
	Hyesue Jang	2018	Yes	5.5
	Mirjam I. Geerlings	2001	No	6.5
Irregular menstrual cycle	Hung-Tse Chou	2021	Yes	5
	Yoko Shimizu	2019	Yes	7.5
	Fu-Dong Li	2015	Yes	7
Breastfeeding (months)	Hung-Tse Chou	2021	Yes	5
	Michelle Heys	2010	No	5
	Fu-Dong Li	2015	No	7
	Jung Eun Yoo	2020	No	7
	Jenna Najar	2020	Yes	7
Surgical menopause	Keiko Kurita	2016	Yes	6
	Yoko Shimizu	2019	Yes	7.5
	Joanne Ryan	2009	Yes	4.5

	J Ryan	2014	Yes	7
	A. Paganini-Hill	2020	Yes	5.5
	Mirjam I. Geerlings	2001	Yes	6.5
Per 1 increase in number of parities	Hung-Tse Chou	2021	Yes	5
	Michelle Heys	2010	Yes	5
	Sujarwoto Sujarwoto	2019	No	6
	Chiara Zucchella	2012	No	6
	Molly Fox	2018	No	7
	Xingyue Song	2020	Yes	7.5
	Martin J. Prince	2018	Yes	5.5
	Jenna Najar	2020	No	7
Early menopause	Sujarwoto Sujarwoto	2019	Yes	6
	Chiara Zucchella	2012	Yes	6
	Yoko Shimizu	2019	No	7.5
	Xingyue Song	2020	Yes	7.5
	J Ryan	2014	No	7
	Natalie L. Rasgon	2005	Yes	6
	Jung Eun Yoo	2020	Yes	7
	Paola Gilsanz	2019	Yes	7
	A. Paganini-Hill	2020	No	5.5
Early menarche	Sujarwoto Sujarwoto	2019	Yes	6
	Yoko Shimizu	2019	Yes	7.5
	Xingyue Song	2020	Yes	7.5
	Natalie L. Rasgon	2005	Yes	6
	Jung Eun Yoo	2020	No	7
	Paola Gilsanz	2019	Yes	7
	Mirjam I. Geerlings	2001	Yes	6.5
	A. Paganini-Hill	2020	Yes	5.5
	Jessica Gong	2022	No	6.5
Stillbirth	Sujarwoto Sujarwoto	2019	Yes	6
	Saima Basit,	2019	Yes	5.5
	Jessica Gong	2022	Yes	6
Late menopause	Robert N. McLay	2003	No	5.5
	Xingyue Song	2020	No	7.5
	Natalie L. Rasgon	2005	No	6
	Jung Eun Yoo	2020	Yes	7
	A. Paganini-Hill	2020	No	5.5
Long reproductive period	Michelle Heys	2010	Yes	5
	Chiara Zucchella	2012	Yes	6
	Yoko Shimizu	2019	No	7.5
	Xingyue Song	2020	No	7.5
	Natalie L. Rasgon	2005	No	6
	Mirjam I. Geerlings	2001	No	6.5

	Jenna Najar	2020	No	7
	A. Paganini-Hill	2020	No	5.5
	Jessica Gong	2022	No	6.5
Late menarche	Hung-Tse Chou	2021	Yes	5
	Chiara Zucchella	2012	Yes	6
	Yoko Shimizu	2019	No	7.5
	Xingyue Song	2020	No	7.5
	Jung Eun Yoo	2020	Yes	7
	Paola Gilsanz	2019	Yes	7

2. Results of the systemic reviews on male-specific reproductive factors.

Factors	Author	Year	Fits with the results of meta-analysis or not	NOS
ADT	Vanessa Sánchez-Martínez	2021	No	3.5
	Kohei Okamoto	2015	Yes	3.5
	Bulent Gunlusoy, Assoc	2016	No	5
	Juan Morote	2017	Yes	3
	Onder Cinar	2021	Yes	4.5
	Yasin Ceylan	2019	No	5
	Shabbir M.H. Alibhai	2016	Yes	6
	Supriya Gupta Mohile	2010	Yes	3.5
	Bum Sik Tae	2018	No	5.5
	Li-Ting Kao	2017	Yes	7
	Kevin T. Nead	2016	No	7
	Myungsun Shim	2020	Yes	6
	Farzin Khosrow-Khavar	2016	Yes	7
	Ravishankar Jayadevappa	2018	No	7
	Seo Hyon Baik	2017	No	5
	Jong Won Kim	2021	No	4
	Kevin T. Nead	2016	No	5
	N. Araújo	2022	No	5
	Hector Alonso-Quiñones	2021	Yes	8
	Peter E. Lonergan	2022	No	4
	Jui-Ming Liu	2022	Yes	6
	Onder Cinar	2021	No	8
	Karl H. Tully	2019	Yes	5
Increased total-T	Elizabeth Barrett-Connor	1999	Yes	5
	Lessov-Schlaggar, C. N.	2005	Yes	5
	Majon Muller	2008	Yes	7
	Sri Suravarapu	2006	Yes	5
	Laure Carcaillon	2013	Yes	6
	Andrew H	2018	No	6
	S.D. Moffat	2004	Yes	6.5
	Margot J Overman	2022	Yes	6.5
Increased bio-T	Elizabeth Barrett-Connor	1999	Yes	5
	Sri Suravarapu	2006	Yes	5
	Laure Carcaillon	2013	No	6
	Andrew H	2018	Yes	6
	Mirjam I. Geerlings	2006	No	7
Increased total-E2	Elizabeth Barrett-Connor	1999	Yes	5
	Lessov-Schlaggar, C. N.	2005	No	5
	Majon Muller	2008	No	7
	Laure Carcaillon	2013	Yes	6

	Andrew H	2018	No	6
	Mirjam I. Geerlings	2002	Yes	7.5
	Giovanni Ravaglia	2007	Yes	6
Increased bio-E2	Elizabeth Barrett-Connor	1999	Yes	5
	Mirjam I. Geerlings	2002	Yes	7.5
	Mirjam I. Geerlings	2006	Yes	7
Increased SHBG	Lessov-Schlaggar, C. N.	2005	No	5
	Erin S. LeBlanc	2010	Yes	6
	Andrew H	2018	No	6
	Majon Muller	2010	No	6
	S.D. Moffat	2004	No	6.5
	Margot J Overman	2022	Yes	6.5

Appendix L Results of index S divergence.

1. Results of index S divergence in female-specific reproductive factors.

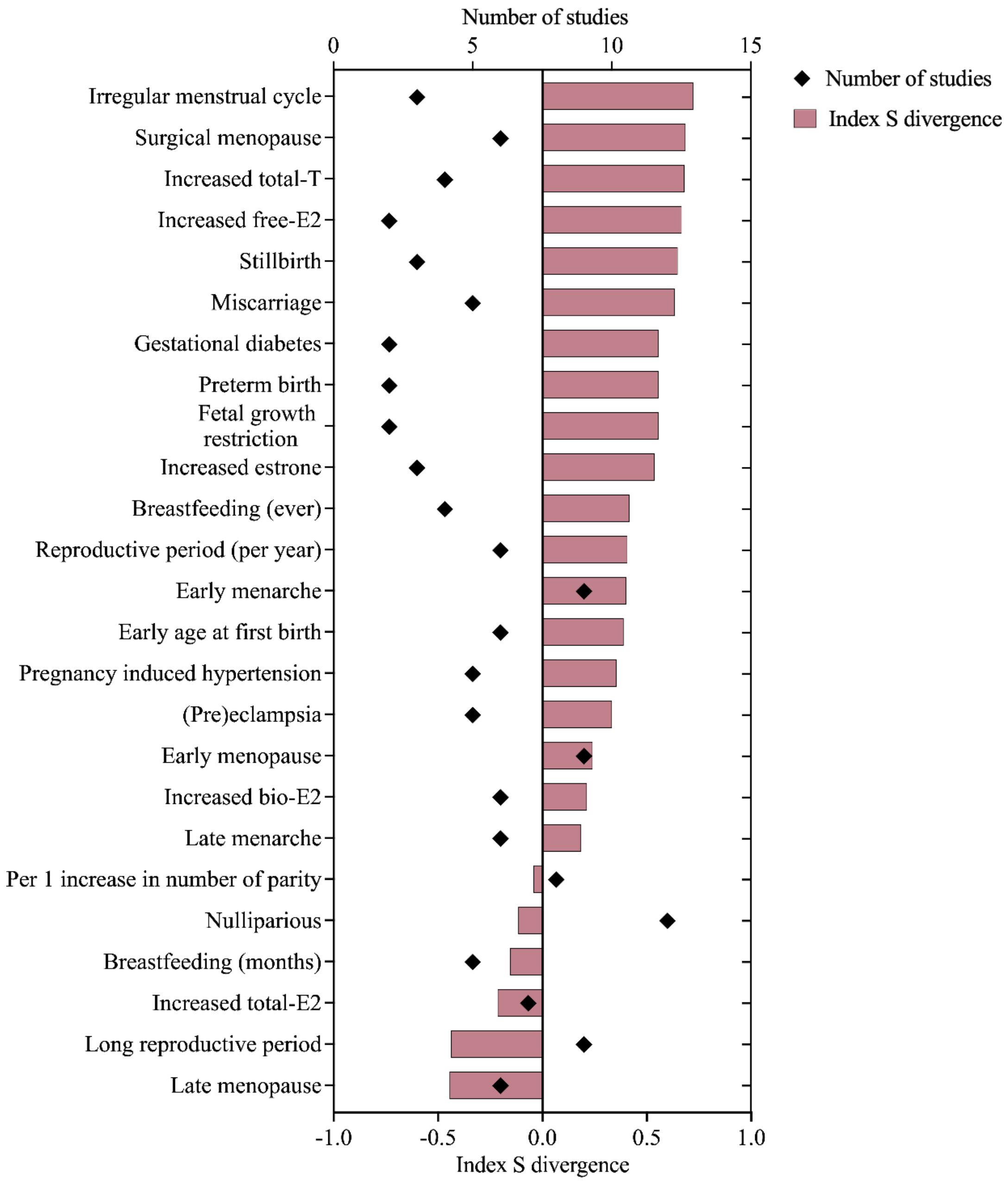

2. Results of index S divergence in male-specific reproductive factors.

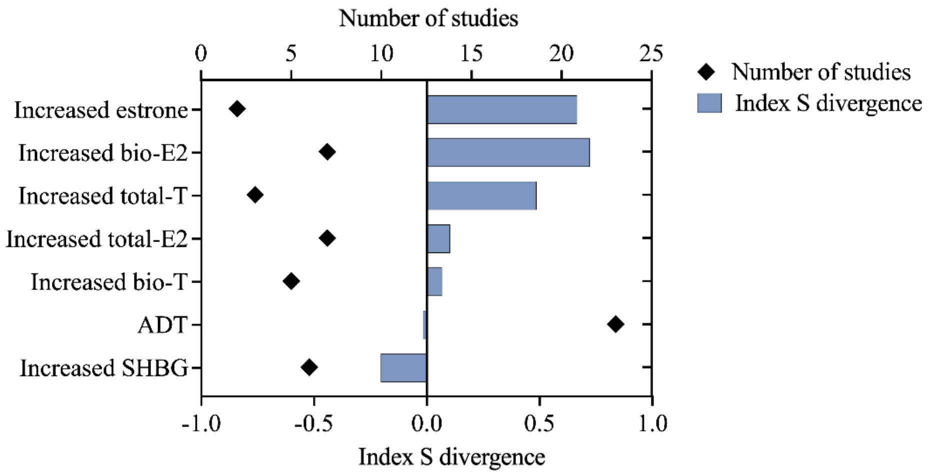

Supplement: Supplementary file 1 — Additional file 1: Appendix A. Newcastle–Ottawa quality assessment scale-cohort studies. Appendix B. Newcastle–Ottawa quality assessment scale-case control studies. Appendix C. Quality assessment according to the Newcastle–Ottawa Scale of cohort studies and case–control studies. Appendix D. Grading approaches used to assess the credibility of meta-analysis. Appendix E. Characteristics of included studies. Appendix F. Measurement of the outcomes on included studies. Appendix G. Characteristics of studies with cognitive decline, dementia, and AD. Appendix H. Results of dose–response analysis between certain factors and relative risks of dementia or cognitive decline. Appendix I. Results of subgroup analysis on ADT. Appendix J. Detailed GRADE score on credibility of the meta-analysis on dementia or cognitive decline. Appendix K. Results of systemic review. Appendix L. Results of index S divergence. [file 12916_2023_3159_MOESM1_ESM.pdf]
